# AutoCOPD–A novel and practical machine learning model for COPD detection using whole-lung inspiratory quantitative CT measurements: a retrospective, multicenter study

**DOI:** 10.1016/j.eclinm.2025.103166

**Published:** 2025-04-03

**Authors:** Fanjie Lin, Zili Zhang, Jian Wang, Cuixia Liang, Jiaxuan Xu, Xiansheng Zeng, Qingpeng Zeng, Huai Chen, Jiayu Zhuang, Yu Ma, Qiao Ma, Raymond Shi, Jingyi Xu, Yuanyuan Li, Liang Yuan, Xinguang Wei, Lulu Wu, Renjun Huang, Tianchi Xiao, Wenhua Liang, Jinping Zheng, Jianxing He, Yun Liu, Zhenyu Liang, Nanshan Zhong, Wenju Lu

**Affiliations:** a State Key Laboratory of Respiratory Disease, Guangdong Key Laboratory of Vascular Disease, National Clinical Research Center for Respiratory Disease, National Center for Respiratory Medicine, Guangzhou Institute of Respiratory Health, The First Affiliated Hospital of Guangzhou Medical University, Guangzhou, Guangdong, PR China; b Department of Thoracic Surgery and Oncology, State Key Laboratory of Respiratory Disease, Guangdong Key Laboratory of Vascular Disease, National Clinical Research Center for Respiratory Disease, National Center for Respiratory Medicine, Guangzhou Institute of Respiratory Health, The First Affiliated Hospital of Guangzhou Medical University, Guangzhou, Guangdong, PR China; c Guangzhou National Lab, Guangzhou, Guangdong, PR China; d Department of Respiratory and Critical Care Medicine, Xiangyang Key Laboratory of Respiratory Health Research, Xiangyang Central Hospital, Affiliated Hospital of Hubei University of Arts and Science, Xiangyang, Hubei, PR China; e Department of Respiratory and Critical Care Medicine, The Second Affiliated Hospital of Xi'an Jiaotong University, Xi'an, Shaanxi, PR China; f Neusoft Medical Systems Co., Ltd. Shenyang, Liaoning, PR China; g Department of Radiology, The Second Affiliated Hospital of Guangzhou Medical University, Guangzhou, Guangdong, PR China

**Keywords:** COPD, Quantitative computed tomography, Machine learning, Detection

## Abstract

**Background:**

The rate of diagnosis for chronic obstructive pulmonary disease (COPD) is low worldwide. Quantitative computed tomography (QCT) parameters add value to quantify alterations in airway and lung parenchyma for COPD. This study aimed to assess the performance of QCT features in COPD detection using a whole-lung inspiratory CT model.

**Methods:**

This multicenter retrospective study was performed on 4106 participants. The derivation cohort containing 1950 participants who enrolled in Guangzhou communities from August 2017 to December 2019, was separated for training and internal validation cohorts, and three external validation cohorts containing 1703 participants were recruited from the public hospitals (Cohort 1: the First Affiliated Hospital of Guangzhou Medical University; Cohort 2: Xiangyang central hospital; Cohort 3: the Second Affiliated Hospital of Xi'an Jiaotong University) in China between April 2017 and May 2024. Questionnaire information, CT reports, and QCT features derived from inspiratory CT were extracted for model development. A novel multimodal framework using eXtreme gradient boosting and hybrid feature selection was established for COPD detection. National Lung Screening Trial (NLST) cohort (n = 453) was applied to validate the multiracial extrapolation and robustness on low-dose CT scans.

**Findings:**

The QCT model (referred to as AutoCOPD) with ten features achieved the highest AUC of 0·860 (95% CI: 0·823–0·898) in the internal validation cohort, and showed excellent discrimination when externally validated [Cohort 1: AUC = 0·915 (95% CI: 0·898–0·931); Cohort 2: AUC = 0·903 (95% CI: 0·864–0·943); Cohort 3: AUC = 0·914 (95% CI: 0·882–0·947); NLST: AUC = 0·881 (95% CI: 0·846–0·915)]. Decision curve analysis demonstrated that AutoCOPD was valuable across a range of COPD risk thresholds between 0·12 and 0·66 compared with intervention in all patients with COPD or no intervention.

**Interpretation:**

Heterogeneous COPD can be well identified using AutoCOPD (https://lwj-lab.shinyapps.io/autocopd/) constructed by a subset of only ten QCT features. It may be generalizable across clinical settings and serve as a feasible tool for early detecting patients with mild or asymptomatic COPD to reduce delayed diagnosis in routine practice.

**Funding:**

The 10.13039/501100001809National Natural Science Foundation of China, Guangzhou Laboratory, 10.13039/501100003453Natural Science Foundation of Guangdong Province, Guangzhou Municipal Science and Technology grant, 10.13039/100013262State Key Laboratory of Respiratory Disease.

**Research in context**

**Evidence before this study**

We searched PubMed for relevant work published up to October 31, 2024, with no language restrictions, using the terms (Pulmonary Disease, Chronic Obstructive [MeSH Terms] OR Pulmonary Disease, Chronic Obstructive [Title/Abstract]) AND (Radiomics [MeSH Terms] OR Radiomics [Title/Abstract]) AND (Machine Learning [MeSH Terms] OR Machine Learning [Title/Abstract] OR Deep Learning [Title/Abstract] OR Artificial Intelligence [MeSH Terms] OR Artificial Intelligence [Title/Abstract] OR algorithm [Title/Abstract] OR CT [Title/Abstract]). This search yielded 27 studies, 11 of which were articles related to chronic obstructive pulmonary disease (COPD) diagnosis. The majority of these studies evaluating COPD detection were small, and did not present external validation or fairness analyses. Reported evaluation metrics of these algorithms varied. Three studies recruited large datasets and proposed reliable prediction models, two of which tailored specifically for the Chinese population. However, one study applied a complex algorithm and did not provide an easy-to-use application. The other study only employed a dataset of computed tomography (CT) scans from clinical COPD without undiagnosed individuals, and evaluated the complicated CT radiomic features.

**Added value of this study**

In this study, we proposed a predictive model (referred to as AutoCOPD), which uses common quantitative CT features extracted from whole-lung inspiratory CT scans to identify COPD in a large multicenter dataset consisting of highly heterogeneous and multi-ethnic participants, and validated its robustness in low- and standard-dose CT images from distinct CT scanners. Our model was implemented on a free and user-friendly online application (https://lwj-lab.shinyapps.io/autocopd/) to aid physicians.

**Implications of all the available evidence**

The proposed model, if further validated, has the potential to reduce delayed diagnosis in clinical practice. The model has been translated into an online risk application which includes rich functions and is highly compatible with the pattern of clinical workflows. Further trials shall be implemented for prospectively evaluating the model.

## Introduction

The underdiagnosis of chronic obstructive pulmonary disease (COPD) is a global issue, especially in developing countries.^1^ A large epidemiologic study in China revealed that approximately 60·2% of patients with COPD did not have self-report typical symptoms, and only 12% of patients with COPD had been tested by pulmonary function test (PFT) before the survey,^2^ demonstrating the insidious early symptoms of COPD and lack of public awareness. While PFT has been the benchmark test for diagnosing COPD,^1^^,^^3^ it is underutilized in primary care settings.^4^ The common reasons include insufficient expertise in performing PFT and low confidence in interpretation.^4^ In contrast, the application of chest computed tomography (CT) is widespread, enabling opportunistic screening for COPD using these scans.^1^^,^^5^

CT has the advantages of non-invasive and high-resolution imaging, allowing for the evaluation of alterations in lung parenchyma, interstitium and airways that occur in COPD.^6^, ^7^, ^8^ However, visual assessment was influenced by the diverse standards among radiologists, and may lead to an underestimation of the condition due to subtle lesions beyond visual perception.^9^^,^^10^ With the rapid advances in machine learning (ML) and image processing algorithms, radiomic features have shown the particular advantages for detecting COPD. Amudala et al.^11^ trained two gradient boosting ML models for identifying COPD on inspiratory low- and standard-dose CT scans, achieving area under the receiver operating characteristic (ROC) curves (AUCs) of 0·87 and 0·9, respectively. Zhou et al.^12^ constructed an intuitive nomogram based on whole-lung radiomic features and achieved an AUC of 0·846 in detecting COPD. It is worth mentioning that the first research enrolled current or former smokers to train models, which may not be applicable to general population. Moreover, the nomogram was developed on clinical COPD with more obvious airflow limitation and changes in CT imaging than those with underlying COPD. These metrics may also be affected by imaging processing algorithms and effects,^13^ making them difficult to explicate and apply in routine practice. Therefore, it is necessary to provide a suitable and unbiased strategy for detecting COPD.

Given the real-world scenario, the objective of this study was to identify COPD from low- and standard-dose CT images using quantitative CT (QCT) parameters with diagnostic value^14^ extracted from whole-lung inspiratory CT scans of highly heterogeneous populations. Easily accessible questionnaire information and CT reports were used to determine whether their detection capabilities were on par with QCT or could be beneficial to the performance of QCT. Seven schemes were trained on the eXtreme gradient boosting (XGBoost) with a combination of ten-fold cross-validation and hybrid feature selection, and the optimal model was selected for evaluation and application.

## Methods

### Study population

The Guangzhou Institute of Respiratory Disease (GIRD) COPD Biobank,^15^ a prospective cohort design was adopted in this study (ChiCTR–CCC–12002950), which comprised various specimens and clinical data for research purposes. All participants from China were retrospectively recruited from four centers from April 2017 to May 2024, containing four subdistricts in Guangzhou, the First Affiliated Hospital of Guangzhou Medical University, Xiangyang central hospital, and the Second Affiliated Hospital of Xi'an Jiaotong University. The inclusion criteria were as follows: 1) aged 35–80 years; 2) completed PFT at least one-time; 3) underwent full-inspiratory CT scans with holding their breath in the supine position; 4) performed both CT and PFT in the same center; 5) participants from communities underwent respiratory epidemiological questionnaire. The exclusion criteria were as follows: 1) incomplete CT images; 2) substantial image artifacts; 3) with history of pulmonary resection; 4) miss rate of questionnaire information over 50%. Finally, a total of 3653 participants from China were included. Among them, 1950 participants (650 patients with COPD and 1300 controls) from communities were assigned to the derivation cohort and randomly divided into training cohort (n = 1560) and internal validation cohort (n = 390) at a ratio of 8:2. They were enrolled with frequency matching by sex and age. According to the label, eligible subjects were first stratified by sex (male and female) between cases and controls. For age, we divided it into specific intervals, containing 35–49 years, 50–59 years, 60–69 years and 70 years or older. Within each sex-age stratum, we set the frequency of cases and controls at a ratio of 1:2, and randomly sampled from the controls to ensure fairness within each stratum. 1186 patients from the First Affiliated Hospital of Guangzhou Medical University, 225 patients from Xiangyang central hospital and 292 patients from the Second Affiliated Hospital of Xi'an Jiaotong University were assigned to the external validation cohort 1, 2, 3, respectively. A random subset of the National Lung Screening Trial (NLST^16^) cohort (n = 453) was used as the external validation cohort 4 to validate the multiracial extrapolation and robustness on low-dose CT scans. The study design is shown in Figure S1. At enrollment, inspiratory low- and standard-dose CT images were collected using a range of acquisition protocols and scanners (Table S1). PFT, questionnaire and clinical data were synchronously recorded.

### Ethics

This study was approved by the Institutional Review Board of Guangzhou Medical University, Ethics Committee of the First Affiliated Hospital (approval number: ES–2024–K060–01) and the National Cancer Institute (NCI) Institutional Review Board (approval number: NLST–1167). Written informed consent was waived due to the retrospective nature.

### Definition of diagnosis

COPD diagnosis in the derivation cohort was defined by prebronchodilator forced expiratory volume in 1 s (FEV1)/forced vital capacity (FVC) <0·7, while patients from hospitals were defined by postbronchodilator FEV1/FVC <0·7. COPD cases in the NLST cohort were pre-defined from the released data. The severity of COPD was graded according to the Global Initiative for Chronic Obstructive Lung Disease guideline.^1^ Small airway dysfunction (SAD) was diagnosed when at least two of prebronchodilator (derivation cohort) or postbronchodilator (external validation cohorts) forced expiratory flow (FEF) 50%, FEF75% and maximal mid-expiratory flow (MMEF) were less than 65% of predicted values.^17^

### PFT

After checking for contraindications to the test, participants underwent standardized prebronchodilator and postbronchodilator spirometry, using the spirometer PF680 (e–Link Care Meditech Co., Ltd., Zhejiang, China^18^) and Master Screen Pneumo spirometer (Vyaire Medical, Inc., Bavaria, Germany). All measurements were performed in conformity with Global Lung Function Initiative reference equations^19^ and American Thoracic Society/European Respiratory Society guidelines.^20^ Concretely, participants received instructions by the respiratory physiologists before performing the prebronchodilator spirometry. They were instructed to sit uprightly with their noses plugged, and form a tight seal around the mouthpiece to inhale rapidly for full inspiration from functional residual capacity. This was followed immediately by a forced exhalation to residual volume. The volume–time curve was inspected during the measurement to ensure no interruption to flow (coughing or glottic closure). At least three acceptable attempts were recorded if the values of FEV1 and FVC were within 0·15 L of each other. For postbronchodilator spirometry, participants were performed approximately 20 min after administering 400 μg of Ventolin via metered dose inhaler. In a few participants who were unable to provide three acceptable maneuvers, data of these cases were included at the investigators' discretion. In this study, the highest values of FEV1, FVC, FEF50%, FEF75%, and MMEF from any of the acceptable curves were recorded as the final values.

### Questionnaire information

Each participant from the derivation cohort completed a paper-based questionnaire, expertly guided by well-trained investigators. The questionnaire used in this study was modified based on previous COPD epidemiological studies in China,^21^, ^22^, ^23^ including 40 questions on demographic, anthropometric characteristics, individual and family disease histories, and other comorbidities. The definitions of these questions are described in detail in the Table S2.

### Clinical data collection

In the derivation cohort, blood samples were obtained from all participants before breakfast, and were immediately processed according to the GIRD COPD study protocol.^15^ Briefly, blood was collected into vacuum blood collection tubes with EDTA–K2 (SANLI Medical Co., Ltd., Hunan, China; #RCXG–005), then was subjected to the routine blood test for collecting peripheral blood eosinophil (EOS) count. Demographic information, spirometry and laboratory data of three external validation cohorts in China were extracted from electronic medical records.

### Structuring of CT report

Each report was written independently by chest-imaging trained radiologists at their respective institutions and then reviewed and modified by a senior radiologist during work. Some words might appear more than once as radiologists use templates when writing reports. We selected representative and high-frequency appearances described in the “Diagnosis” section of all reports, merged semantically similar labels, and recorded their anatomical locations according to the whole lung and each lobe. Ultimately, 65 variables were used in this study, whose definitions and recording format are shown in Table S2.

### CT image analysis

CT images were analyzed using the *NeuLungCare*–*QA* software (version 1·0, Neusoft Medical Systems Co., Ltd. Shenyang, Liaoning, China) which was commercially certified and validated in a previous study^14^ for quantifying CT emphysema and airways lesions. A total of 47 QCT features were extracted in this study (Table S3). Briefly, CT emphysema was quantified on full-inspiration images using low-attenuating area below −950 Hounsfield Units (HU) (LAA-950) and −910 HU (LAA-910) for the whole lung and each lobe separately (Figure S2).

CT airway measurements were generated from the 0th to the 4th generation bronchi (Figure S2). Airway wall area percentage (%WA) was measured by dividing total tracheal or bronchial cross-sectional area into wall area. For airway dimension measurements, the airway length was defined as the distance from the beginning to the end of the branching points by placing a smoothed centerline through the lumen. For each airway, the wall thickness (WT) and lumen diameter (LD) were calculated at intervals of 2 mm. For the trachea, the maximal, minimal, and mean values of WT and LD were generated from five measurement results; for the 1st–4th generation bronchi, the maximal, minimal, and mean values of WT and LD were generated by combining the corresponding measurement results of bronchi of the same generation (measurement times depending on their length).

### Model development and evaluation

#### Data preprocessing

Data preprocessing steps are as follows: 1) performed a logical consistency check and removed outliers; 2) encoded the binary category variables into integers 1 and 2 (Table S2); 3) encoded the multi-category variables into consecutive integers starting from 1 according to the intrinsic order (Table S2); 4) imputed the missing data in the training cohort and internal validation cohort separately to avoid data leakage.

#### Model construction and validation

An advanced algorithm for handling missing data and assembling nonlinear model, XGBoost^24^^,^^25^ was used to detect COPD. To determine the optimal scheme, seven models were trained based on questionnaire, QCT, CT report, and their combinations: questionnaire, QCT, CT report, questionnaire and QCT, questionnaire and CT report, QCT and CT report, as well as questionnaire, QCT and CT report. Aiming to decrease the probability of overfitting and develop a practical prediction model, a hybrid feature selection method was adopted. Firstly, variables with zero and near-zero variance were excluded due to the risk of non-informative predictors. Secondly, original XGBoost models were fitted using all available variables in different unimodal schemes (questionnaire, QCT, and CT report), and then we separately extracted the top ten variables interpreted using the SHapley Additive exPlanation (SHAP) values^26^ to train the final XGBoost models. The original multimodal models were composed of the feature combinations that constitute the final unimodal models, whereas the final multimodal models consisted of the top ten selected features from these combinations. The models' hyperparameters were tuned by Bayesian optimization based on a ten-fold cross-validation procedure. Additionally, the models’ performance was measured using the logarithmic loss function and the models with the highest AUC were selected.

### Model evaluation

Model validation was originally conducted on the internal validation cohort. AUC, sensitivity, specificity, accuracy, negative predictive value (NPV), positive predictive value (PPV), and F1 score were estimated. The corresponding 95% confidence intervals (CIs) were computed with 2000 stratified bootstrap replicates and the significance of the AUCs among all models was computed using the DeLong's method.^27^ The optimal model was selected based on the overall discriminatory ability.

Furthermore, the optimal model was evaluated on the external validation cohorts using the above metrics. Calibration was assessed graphically using lowess curve. The overall calibration performance of the model was evaluated using Brier score^28^ and Hosmer–Lemeshow (HL) test.^29^ The potential clinical utility of the model was estimated using decision curve analysis (DCA).

### Subgroup analysis

To comprehensively explore the applicability of the optimal model, the model's performance was evaluated by stratifying the participants according to sex, age, body mass index (BMI), smoking status, educational level, CT apparatus and slice thickness, chronic respiratory comorbidities, allergic disease, the classification of SAD, peripheral blood EOS counts, and representative appearances of CT imaging.

### Comparison of model and representative screening tool

COPD Screening Questionnaire (COPD-SQ) is a well-validated tool in first-level screening for COPD in China.^23^ It incorporates seven questions in terms of age, smoking pack-years, BMI, cough, tachypnea, exposure of biomass smoke and family history of respiratory diseases (COPD, asthma, chronic bronchitis and emphysema). When the COPD-SQ score is greater than or equal to 16, it implies that the subject may suffer from COPD. As a benchmarking tool, we assessed it and the optimal model with different thresholds in the internal validation cohort to verify the screening performance of the model.

### Feature importance

The SHAP values were applied for quantifying the importance of features on the models’ prediction. Each SHAP value measures how much each feature contributes, either positively or negatively, to a single COPD risk assigned by the model.

### Implementation

A free and user-friendly web application was developed to enhance the utility of the optimal model in clinical practice.

### Statistics

All statistical analyses were performed using the *R* (version 4·4·0) and *RStudio* (version 2023·12·0). The primary packages were listed in Table S4. Data with normal distribution were presented as mean ± standard deviation (SD), while with non-normal distribution were presented as the median (M) and interquartile range (IQR). Shapiro–Wilk test was used to test the normality of independent variables. Categorical variables were presented as numbers (%). The Student's unpaired t-test or Kruskal–Wallis test was used for quantitative variables, and chi-square test or Fisher exact test was used for categorical variables. *P* value < 0·05 was considered statistically significant. The sample size was calculated beforehand using the online software (https://mvansmeden.shinyapps.io/BeyondEPV/) by Riley RD et al.^30^ (Parameter: number of candidate predictors = 10; events fraction = 0·34; criterion value rMPSE = 0·05), resulting in at least 870 participants and 29·7 events per variable. The actual number of training cohort exceeded the minimum requirement to allow for good model fitting. The primary outcome was COPD detection, with further analyses for different designated subgroups. Missing data were considered to be missing at random (MAR) and not missing at random (NMAR) which imputed using the missForest (version 1·5) algorithm.^31^

### Role of the funding source

The funders had no role in the study design, data collection, data analysis, data interpretation, or writing of report.

## Results

### Characteristics of cohorts

In total, 4106 participants (1880 patients with COPD and 2226 controls) were included in this study. The average age was 62·9 years ± 8·1 (SD); 2984 were male and 1122 were female. The training cohort included 520 patients with COPD and 1040 controls, and the internal validation cohort included 130 patients with COPD and 260 controls. The distribution of the participants in four external validation cohorts was as follows: 801 patients with COPD and 385 controls from cohort 1; 117 patients with COPD and 108 controls from cohort 2; 200 patients with COPD and 92 controls from cohort 3; 112 patients with COPD and 341 controls from cohort 4. Significant differences were observed in BMI, educational level, smoking status, chronic respiratory comorbidities, allergic diseases and LAA-950 between the COPD and control groups (*P* < 0·05) in the derivation cohort (Table 1). The demographics of the training and internal validation cohorts appeared to be similar. Detailed information is provided in Tables S5–S6.

**Table 1:** Demographic and clinical characteristics in the overall derivation cohort and split cohorts.

Characteristics	Derivation cohort		*P* value	Training cohort		*P* value	Internal validation cohort		*P* value
	Control (n = 1300)	COPD (n = 650)		Control (n = 1040)	COPD (n = 520)		Control (n = 260)	COPD (n = 130)	
Age, yrs, M (IQR)	63·0 (58·0–67·0)	63·5 (59·0–68·0)	0·443	63·0 (58·8–67·0)	64·0 (59·0–68·0)	0·199	64·0 (58·0–69·0)	63·0 (58·0–68·8)	0·464
Age group, n (%)									
35–49	48 (3·70)	24 (3·70)	1	37 (3·60)	19 (3·70)	0·993	11 (4·20)	5 (3·80)	0·951
50–59	322 (24·8)	161 (24·8)		259 (24·9)	126 (24·2)		63 (24·2)	35 (26·9)	
60–69	716 (55·1)	358 (55·1)		582 (56·0)	293 (56·3)		134 (51·5)	65 (50·0)	
≥70	214 (16·5)	107 (16·5)		162 (15·6)	82 (15·8)		52 (20·0)	25 (19·2)	
Sex, n (%)									
Male	944 (72·6)	472 (72·6)	1	756 (72·7)	382 (73·5)	0·793	188 (72·3)	90 (69·2)	0·607
Female	356 (27·4)	178 (27·4)		284 (27·3)	138 (26·5)		72 (27·7)	40 (30·8)	
BMI, n (%)									
<18·5	49 (3·80)	42 (6·50)	<0·001	37 (3·60)	29 (5·60)	<0·001	12 (4·60)	13 (10·0)	0·093
18·5–23·9	711 (54·7)	408 (62·8)		558 (53·7)	328 (63·1)		153 (58·8)	80 (61·5)	
24–27·9	448 (34·5)	178 (27·4)		367 (35·3)	144 (27·7)		81 (31·2)	34 (26·2)	
≥28	92 (7·10)	22 (3·40)		78 (7·50)	19 (3·70)		14 (5·40)	3 (2·30)	
Smoking, n (%)									
Never	639 (49·2)	252 (38·8)	<0·001	494 (47·5)	197 (37·9)	<0·001	145 (55·8)	55 (42·3)	0·016
Former or current	661 (50·8)	398 (61·2)		546 (52·5)	323 (62·1)		115 (44·2)	75 (57·7)	
Smoking pack-years, n (%)									
0	639 (49·2)	252 (38·8)	<0·001	494 (47·5)	197 (37·9)	0·003	145 (55·8)	55 (42·3)	0·086
1–14	134 (10·3)	71 (10·9)		106 (10·2)	55 (10·6)		28 (10·8)	16 (12·3)	
15–30	191 (14·7)	115 (17·7)		157 (15·1)	93 (17·9)		34 (13·1)	22 (16·9)	
≥30	336 (25·8)	212 (32·6)		283 (27·2)	175 (33·7)		53 (20·4)	37 (28·5)	
Education, n (%)									
Primary school or lower	94 (7·20)	63 (9·70)	0·011	74 (7·10)	51 (9·80)	0·019	20 (7·70)	12 (9·20)	0·513
Middle school	392 (30·2)	230 (35·4)		302 (29·0)	180 (34·6)		90 (34·6)	50 (38·5)	
High school	528 (40·6)	243 (37·4)		431 (41·4)	192 (36·9)		97 (37·3)	51 (39·2)	
Associate's degree	202 (15·5)	75 (11·5)		165 (15·9)	63 (12·1)		37 (14·2)	12 (9·20)	
Bachelor's degree or higher	84 (6·50)	39 (6·00)		68 (6·50)	34 (6·50)		16 (6·20)	5 (3·80)	
GOLD stage, n (%)									
1	NA	264 (40·6)	NA	NA	212 (40·8)	NA	NA	52 (40·0)	NA
2	NA	297 (45·7)	NA	NA	239 (46·0)	NA	NA	58 (44·6)	NA
3	NA	75 (11·5)	NA	NA	60 (11·5)	NA	NA	15 (11·5)	NA
4	NA	14 (2·20)	NA	NA	9 (1·70)	NA	NA	5 (3·84)	NA
Underlying diseases (yes), n (%)									
Chronic respiratory disease	193 (14·8)	212 (32·6)	<0·001	154 (14·8)	165 (31·7)	<0·001	39 (15·0)	47 (36·2)	<0·001
Hypertension	419 (32·2)	193 (29·7)	0·277	332 (31·9)	150 (28·8)	0·237	87 (33·5)	43 (33·1)	0·237
Diabetes	140 (10·8)	59 (9·10)	0·278	108 (10·4)	50 (9·60)	0·700	32 (12·3)	9 (6·90)	0·145
Heart disease	169 (13·0)	86 (13·2)	0·943	138 (13·3)	70 (13·5)	0·979	31 (11·9)	16 (12·3)	1
Stroke	31 (2·40)	15 (2·30)	1	24 (2·30)	13 (2·50)	0·953	7 (2·70)	2 (1·50)	0·724
Allergic disease	260 (20·0)	167 (25·7)	0·005	208 (20·0)	131 (25·2)	0·023	52 (20·0)	36 (27·7)	0·113
LAA-950, %, M (IQR)									
Lung	3·00 (1·00–7·00)	11·0 (7·00–16·0)	<0·001	3·00 (1·00–7·00)	11·0 (6·00–15·3)	<0·001	3·00 (1·00–7·00)	11·0 (7·00–17·0)	<0·001
Left upper lobe	4·00 (1·00–8·00)	12·0 (7·00–18·0)	<0·001	4·00 (1·00–8·00)	12·0 (7·00–18·0)	<0·001	3·50 (1·00–8·00)	11·5 (8·00–19·0)	<0·001
Left lower lobe	2·00 (1·00–5·00)	10·0 (5·00–15·0)	<0·001	2·00 (1·00–5·00)	9·0 (5·00–15·0)	<0·001	2·00 (1·00–6·00)	10·0 (5·00–15·0)	<0·001
Right upper lobe	3·00 (0–7·00)	10·0 (6·00–17·0)	<0·001	3·00 (0–7·00)	10·0 (6·00–16·0)	<0·001	3·00 (0–7·00)	11·0 (6·00–18·0)	<0·001
Right middle lobe	4·00 (1·00–8·00)	12·0 (7·00–18·0)	<0·001	4·00 (1·00–8·00)	13·0 (7·00–18·0)	<0·001	4·00 (1·00–8·00)	12·0 (7·00–19·0)	<0·001
Right lower lobe	2·00 (0–5·00)	9·00 (4·00–14·0)	<0·001	2·00 (0–5·00)	9·00 (4·00–14·0)	<0·001	2·00 (0–5·00)	9·00 (5·00–14·0)	<0·001

Abbreviations: COPD, chronic obstructive pulmonary disease; Yrs, years; M (IQR), median, interquartile range; BMI, body mass index; GOLD, Global Initiative for Chronic Obstructive Lung Disease; LAA-950, low-attenuating area below −950 Hounsfield Units; NA, not applicable.

### Selection of model features

A total of 152 features (40 questionnaire features, 47 QCT features and 65 CT report features) were extracted. After variance analysis, 135 variables were finally retained. The features involved in the seven schemes are shown in Table S7.

### Performance of models

**Fig. 1:**
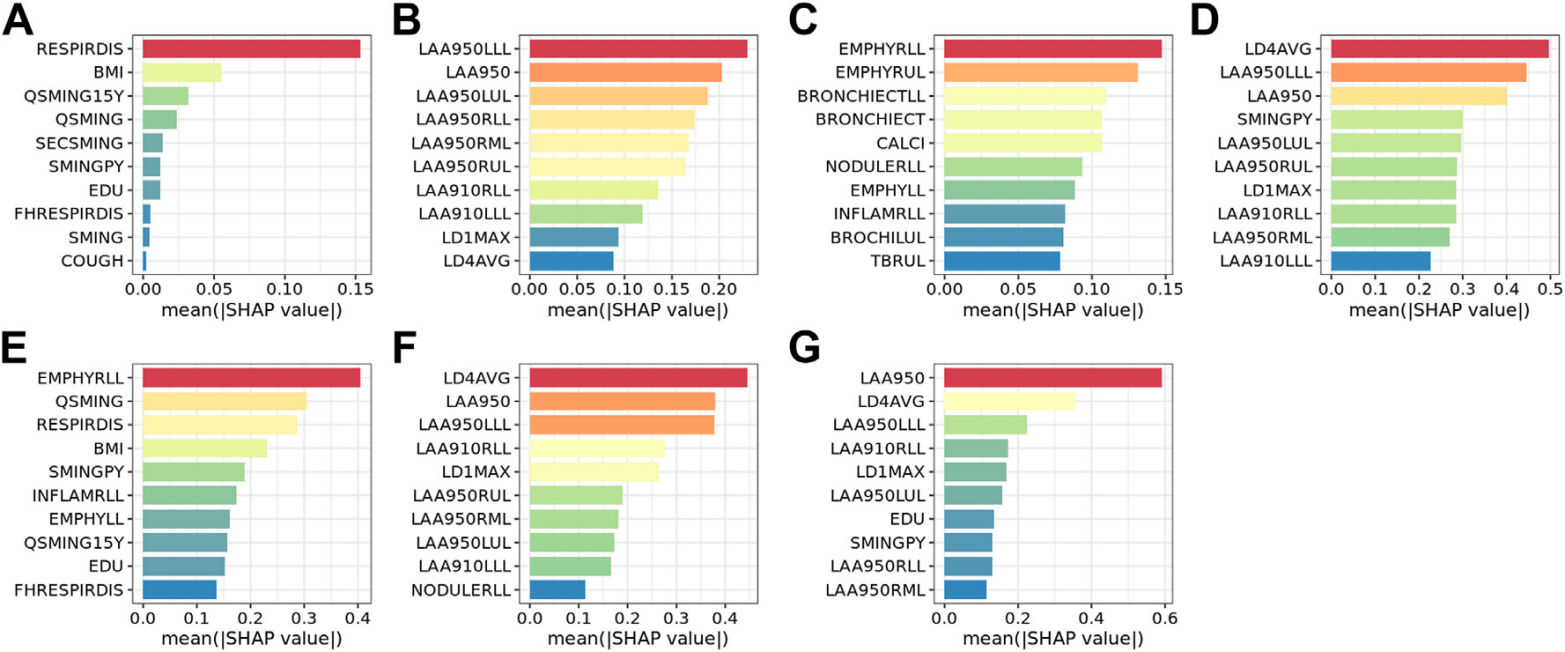
**SHAP summary bar plots. (A**–**G)** Top ten features of seven schemes, namely questionnaire **(A)**, QCT **(B)**, CT report **(C)**, questionnaire and QCT **(D)**, questionnaire and CT report **(E)**, QCT and CT report **(F)**, as well as questionnaire, QCT and CT report **(G)**. The feature importance was assessed using mean absolute SHAP values. Based on the candidate features from the unimodal models, the multimodal models were composed of the combinations of these features. The location of the bar on the x-axis represents the feature' s SHAP value, while its color represents the actual value (red representing higher values). A feature’ s SHAP value represents the contribution of the specific feature to the performance of model. The order of features is ranked by their mean absolute SHAP values. Abbreviations: SHAP, SHapley Additive exPlanation; CT, computed tomography; QCT, quantitative computed tomography.

**Fig. 2:**
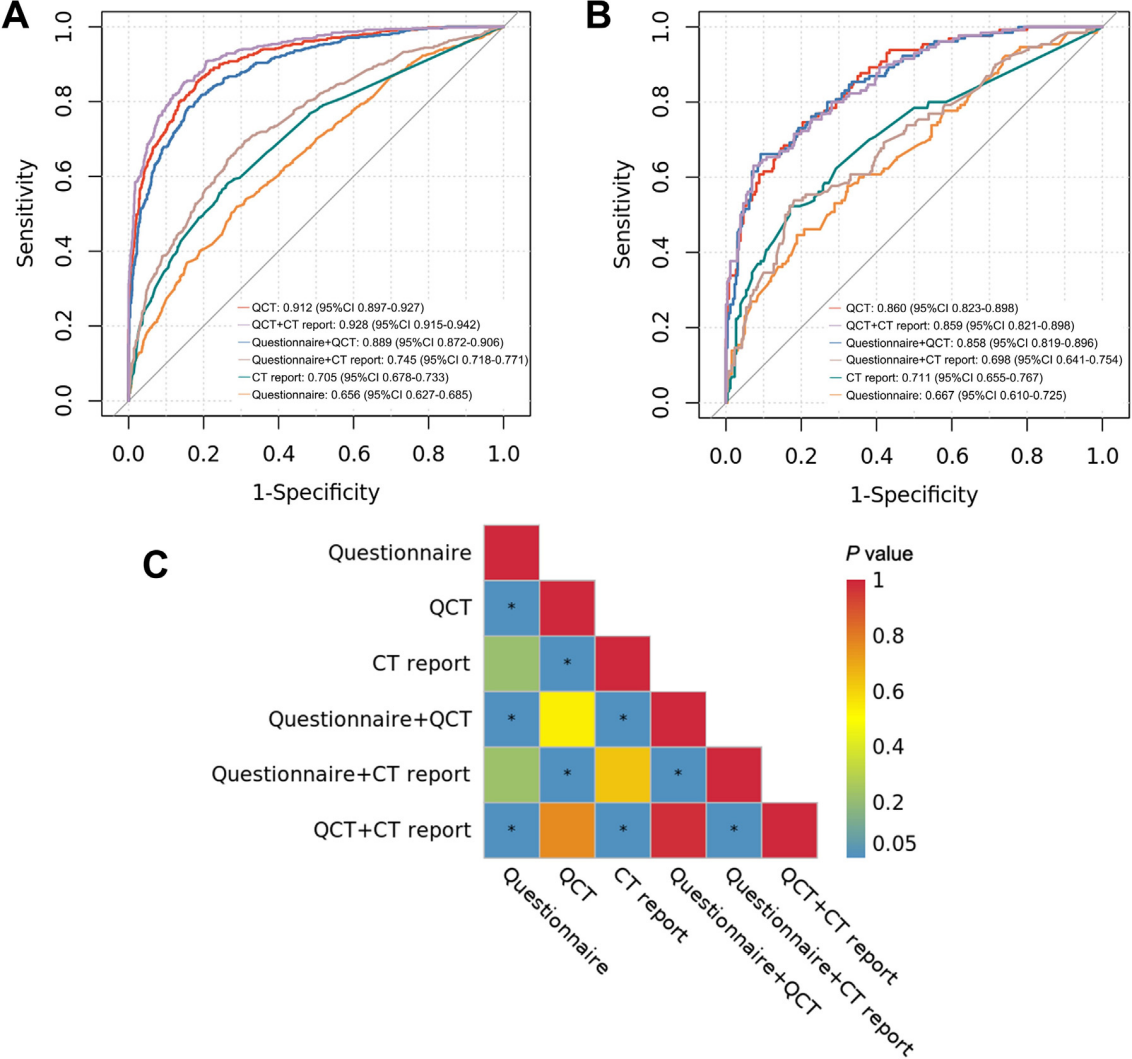
**Model evaluation of six schemes. (A** and **B)** ROC curves of unimodal and multimodal models in predicting COPD in the training **(A)** and internal validation cohorts **(B)**. **(C)** Heatmap for the significance of the AUCs computed using the DeLong's method. The color represents the actual *P* value, with red indicating higher value and blue indicating lower value. “∗” represents the *P* value < 0.05. Abbreviations: ROC, receiver operating characteristic; AUC, area under the receiver operating characteristic curve; COPD, chronic obstructive pulmonary disease.

By SHAP analysis, ten most informative variables in each original model are shown in Fig. 1. Full-feature model was abandoned due to the inclusion of only QCT and questionnaire features. The performance of final models in six other schemes is shown in Table 2 and Fig. 2. Overall, none of the models were overfitted. In the internal validation cohort, questionnaire [AUC = 0·667 (95% CI: 0·610–0·725)] and CT report [AUC = 0·711 (95% CI: 0·655–0·767)] models, even for their combination [AUC = 0·698 (95% CI: 0·641–0·754)], demonstrated poor robustness. On the contrary, QCT scheme maintained robust classification capability [AUC = 0·860 (95% CI: 0·823–0·898)]. Moreover, the inclusion of QCT could not improve the capability for detecting COPD of questionnaire [AUC = 0·858 (95% CI: 0·819–0·896)] and CT report [AUC = 0·859 (95% CI: 0·821–0·898)]. DeLong's test also showed that there was no significant difference in the AUCs among the QCT model and all combined models (*P* > 0·05; Fig. 2). Considering the convenience of clinical application, the best scheme was QCT model, hereafter referred to as AutoCOPD. Other evaluation indexes of AutoCOPD also performed well (Table 2). For hyperparameters of AutoCOPD, we had max-depth = 4, min-child-weight = 5, eta = 0·03,643,557, gamma = 0·5,233,711, colsample-bytree = 0·8,115,991, subsample = 0·5,649,934, and nround = 173. Other hyperparameters were set as default values.

**Table 2:** COPD detection performance for XGBoost models based on different schemes.

Schemes	AUC (95% CI)	Sensitivity (%)	Specificity (%)	Accuracy (%)	PPV (%)	NPV (%)	F1 score
QCT							
Training cohort	0·912 (0·897–0·927)	85·8	81·3	82·8	69·7	92·0	0·769
Internal validation cohort	0·860 (0·823–0·898)	74·6	79·6	77·9	64·7	86·3	0·693
QCT + CT report							
Training cohort	0·928 (0·915–0·942)	85·2	85·2	85·2	74·2	92·0	0·793
Internal validation cohort	0·859 (0·821–0·898)	69·2	86·9	81·0	72·6	85·0	0·709
Questionnaire + QCT							
Training cohort	0·889 (0·872–0·906)	78·7	83·8	82·1	70·9	88·7	0·746
Internal validation cohort	0·858 (0·819–0·896)	66·2	90·8	82·6	78·2	84·3	0·717
Questionnaire + CT report							
Training cohort	0·745 (0·718–0·771)	69·4	69·0	69·2	52·9	81·9	0·600
Internal validation cohort	0·698 (0·641–0·754)	53·8	81·9	72·6	59·8	78·0	0·567
CT report							
Training cohort	0·705 (0·678–0·733)	58·1	73·7	68·5	52·4	77·8	0·551
Internal validation cohort	0·711 (0·655–0·767)	52·3	83·1	72·8	60·7	77·7	0·562
Questionnaire							
Training cohort	0·656 (0·627–0·685)	51·9	71·2	64·7	47·4	74·7	0·495
Internal validation cohort	0·667 (0·610–0·725)	44·6	81·2	69·0	54·2	74·6	0·489

Abbreviations: QCT, quantitative computed tomography; CT, computed tomography; AUC, area under the receiver operating characteristic curve; CI, confidence interval; NPV, negative predictive value; PPV, positive predictive value. The AUC, sensitivity, specificity, accuracy, NPV, PPV and F1 score were calculated using Youden's index.

### Feature importance of AutoCOPD

**Fig. 3:**
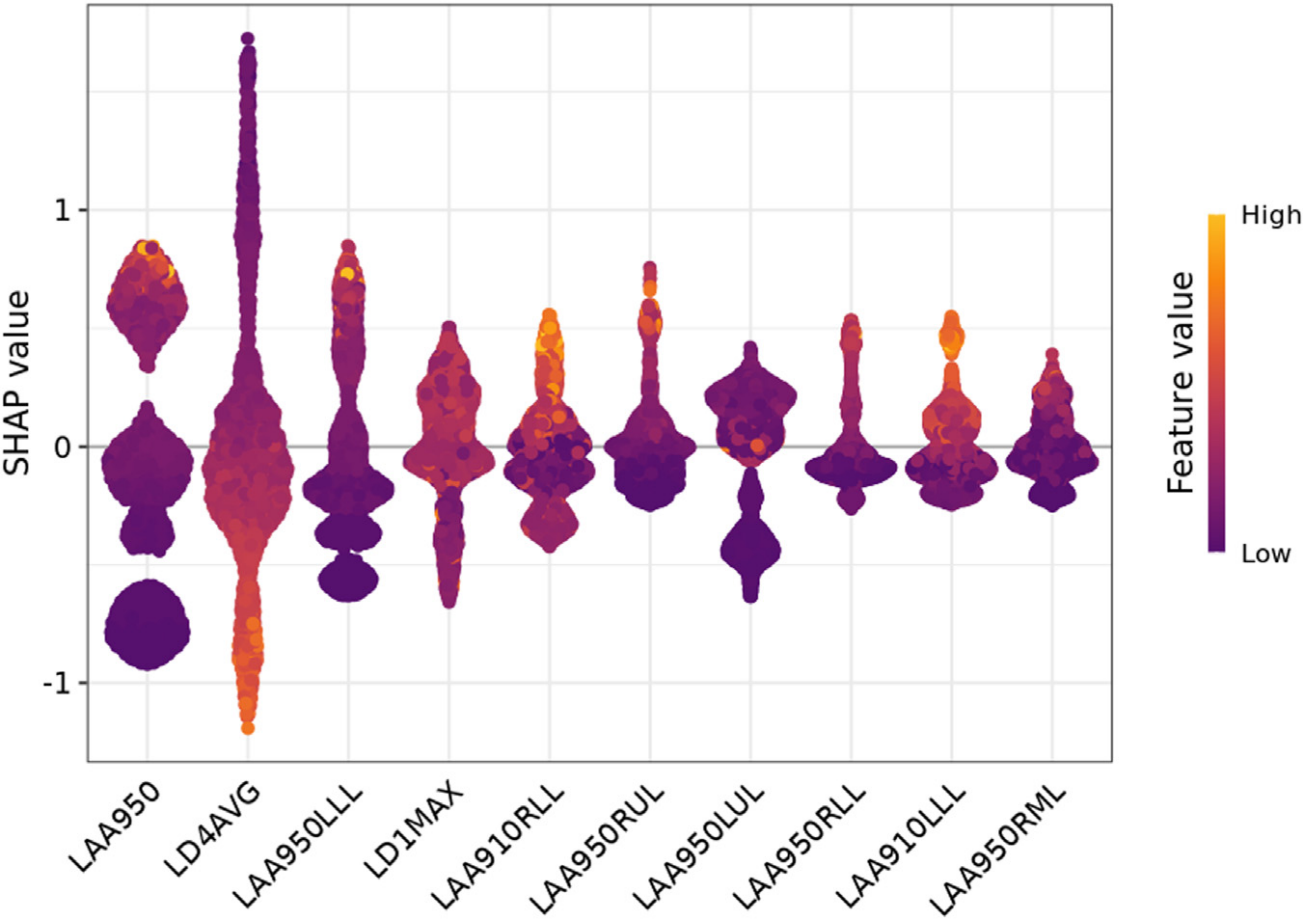
**SHAP beeswarm summary plot for ten features of AutoCOPD.** The SHAP value (y-axis) of a feature represents the contribution of a specific feature to the COPD development, with positive values indicating the contribution of increasing the risk score and negative values indicating the contribution of decreasing the score. The location of the dot on the y-axis represents the feature' s SHAP value, while its color represents the actual feature values of each cluster, with yellow indicating higher feature value and purple indicating lower feature value. The dots are stacked vertically to show their density. The order of features is ranked by their mean absolute SHAP values. Abbreviations: SHAP, SHapley Additive exPlanation; COPD, chronic obstructive pulmonary disease.

By beeswarm plot of SHAP analysis in the training cohort (Fig. 3), the contribution of variables in AutoCOPD from high to low is LAA-950, average LD of the 4th generation, LAA-950 of left lower lobe, max LD of the 1st generation, LAA-910 of right lower lobe, LAA-950 of right upper lobe, LAA-950 of left upper lobe, LAA-950 of right lower lobe, LAA-910 of left lower lobe and LAA-950 of right middle lobe. Intuitively, higher LAA-950 of whole lung and each lobe, higher LAA-910 of left lower and right lower lobes, and wider max LD of the 1st generation all contributed to a heightened risk of COPD, while narrower average LD of the 4th generation contributed to a lower risk.

### Evaluation of AutoCOPD

**Fig. 4:**
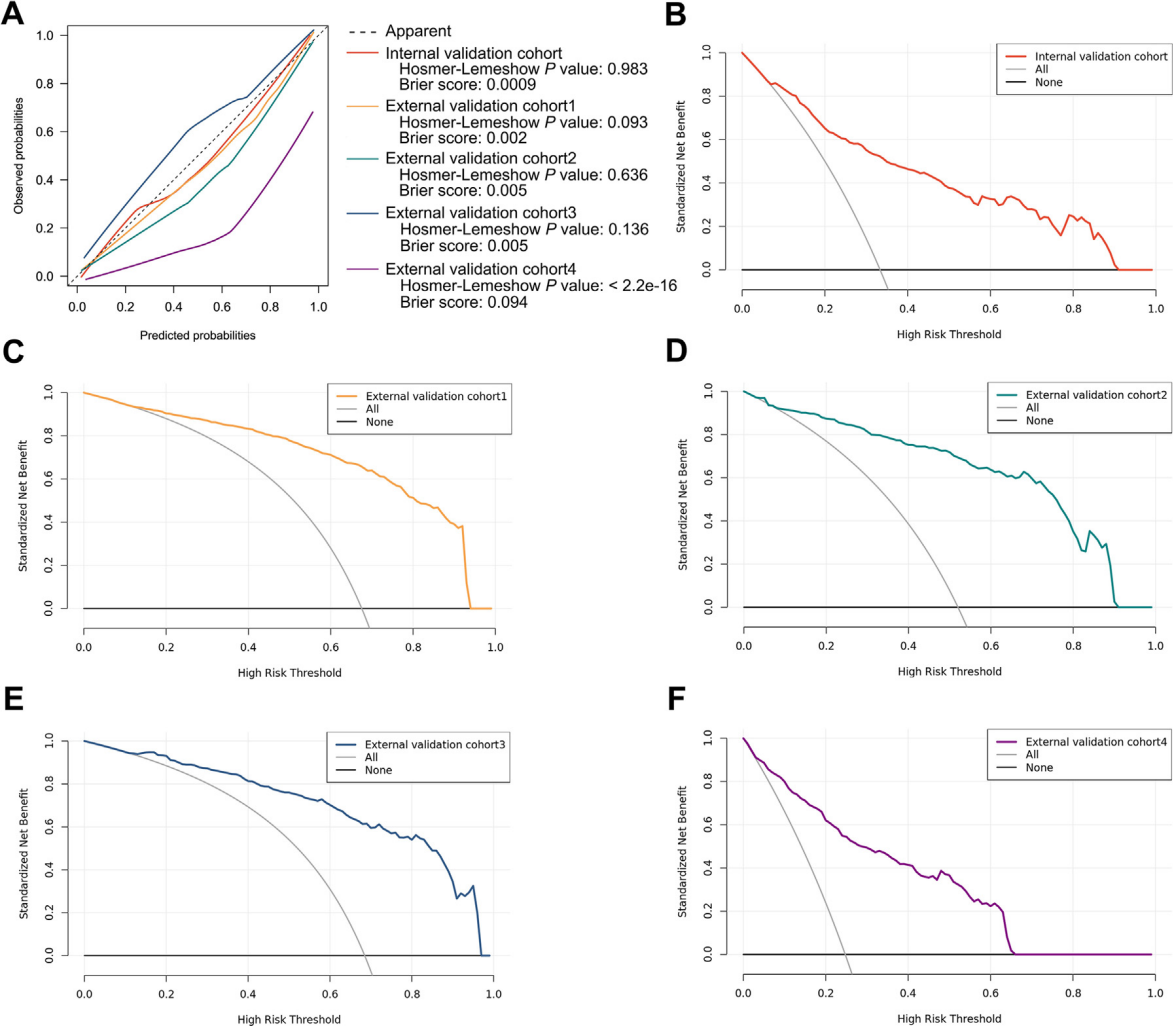
**Calibration and DCA for AutoCOPD. (A)** Observed versus predicted COPD risk in the internal and external validation cohorts. **(B–F)** The plot shows the standardized net benefit (y-axis) across a range of COPD risk thresholds (x-axis) of AutoCOPD compared with intervention in all participants (all) or no intervention (none) in the internal validation cohort **(B)**, external validation cohort 1 **(C)**, external validation cohort 2 **(D)**, external validation cohort 3 **(E)**, and external validation cohort 4 **(F)**. Abbreviations: DCA, decision curve analysis; COPD, chronic obstructive pulmonary disease.

The AutoCOPD was further evaluated in four external validation cohorts without imputation to verify the extrapolation. As depicted in Table 3, AutoCOPD demonstrated excellent predictive performance, manifested by AUCs of 0·915 (95% CI: 0·898–0·931), 0·903 (95% CI: 0·864–0·943), 0·914 (95% CI: 0·882–0·947), and 0·881 (95% CI: 0·846–0·915). Calibration performance of AutoCOPD appeared to be well-calibrated in three external validation cohorts from China, although it seemed to underestimate or overestimate the probability of COPD (Fig. 4), and HL test was not statistically significant (all *P* > 0·05). The overall DCA demonstrated the net benefits of AutoCOPD were greater than zero across a range of COPD risk thresholds between 0·12 and 0·66 compared with the strategies of managing all patients with COPD as if they do or do not have COPD (Fig. 4), even if the model illustrated poor calibration performance when validated in the NLST cohort (*P* < 2·2e-16). Given that lung segmentation and airway tree modeling require significant image preprocessing, we investigated the scenario of employing only LAA-950 data to detect COPD. The results showed that despite its overall robustness was weaker than complete AutoCOPD (Table 4), it had acceptable predictive performance (Table 3), which is favorable for the model to be more widely adopted in screening.

**Table 3:** COPD detection performance for AutoCOPD and LAA-950.

Cohorts	AUC (95% CI)	Sensitivity (%)	Specificity (%)	Accuracy (%)	PPV (%)	NPV (%)	F1 score
**AutoCOPD**							
Training cohort	0·912 (0·897–0·927)	85·8	81·3	82·8	69·7	92·0	0·769
Internal validation cohort	0·860 (0·823–0·898)	74·6	79·6	77·9	64·7	86·3	0·693
External validation cohort 1	0·915 (0·898–0·931)	86·6	80·5	84·7	90·2	74·3	0·884
External validation cohort 2	0·903 (0·864–0·943)	84·6	85·2	84·9	86·1	83·6	0·853
External validation cohort 3	0·914 (0·882–0·947)	77·5	89·1	81·2	93·9	64·6	0·849
External validation cohort 4	0·881 (0·846–0·915)	81·3	81·2	81·2	58·7	93·0	0·682
**LAA-950**							
Training cohort	0·830 (0·809–0·851)	68·7	81·8	77·4	65·4	83·9	0·670
Internal validation cohort	0·829 (0·787–0·871)	67·7	83·1	77·9	66·7	83·7	0·672
External validation cohort 1	0·868 (0·846–0·891)	81·1	82·1	81·5	90·4	67·7	0·855
External validation cohort 2	0·881 (0·837–0·925)	71·8	90·7	80·9	89·4	74·8	0·796
External validation cohort 3	0·890 (0·849–0·931)	76·5	83·7	78·8	91·1	62·1	0·832
External validation cohort 4	0·868 (0·828–0·908)	76·8	86·2	83·9	64·7	91·9	0·702

Abbreviations: AUC, area under the receiver operating characteristic curve; CI, confidence interval; NPV, negative predictive value; PPV, positive predictive value; LAA-950, low-attenuating area below −950 Hounsfield Units. The AUC, sensitivity, specificity, accuracy, NPV, PPV and F1 score were calculated using Youden's index.

**Table 4:** The dissimilarity of AutoCOPD and LAA-950 for COPD detection.

Cohorts	AutoCOPD	LAA-950	*P* value
	AUC (95% CI)	AUC (95% CI)	
Training cohort	0·912 (0·897–0·927)	0·830 (0·809–0·851)	5·72e-35
Internal validation cohort	0·860 (0·823–0·898)	0·829 (0·787–0·871)	0·014
External validation cohort 1	0·915 (0·898–0·931)	0·868 (0·846–0·891)	6·44e-12
External validation cohort 2	0·903 (0·864–0·943)	0·881 (0·837–0·925)	0·457
External validation cohort 3	0·914 (0·882–0·947)	0·890 (0·849–0·931)	0·043
External validation cohort 4	0·881 (0·846–0·915)	0·868 (0·828–0·908)	0·273

Abbreviations: AUC, area under the receiver operating characteristic curve; CI, confidence interval. The AUC was calculated using Youden's index, and the *P* value was calculated using DeLong's test.

In subgroup analysis, AutoCOPD exhibited relatively robust performance in both internal and external validation cohorts without imputation, with AUCs ranging from 0·714 to 1 (Table 5 and Table S8) and the median of AUCs being 0·860. This performance was not significantly affected by modifications to CT acquisition protocol or demographics information.

**Table 5:** COPD detection performance for AutoCOPD in various subgroups of the internal validation cohort.

Subgroups	AUC (95% CI)	Sensitivity (%)	Specificity (%)	Accuracy (%)	PPV (%)	NPV (%)	F1 score
Overall performance	0·860 (0·823–0·898)	74·6	79·6	77·9	64·7	86·3	0·693
Sex							
Male (n = 278)	0·875 (0·833–0·917)	81·1	78·7	79·5	64·6	89·7	0·719
Female (n = 112)	0·832 (0·753–0·910)	90·0	63·9	73·2	58·1	92·0	0·706
Age							
35–49 years (n = 16)	0·927 (0·773–1·00)	80·0	100	93·8	100	91·7	0·889
50–59 years (n = 98)	0·766 (0·671–0·860)	94·3	55·6	69·4	54·1	94·6	0·688
60–69 years (n = 199)	0·881 (0·832–0·929)	90·8	69·4	76·4	59·0	93·9	0·715
70–80 years (n = 77)	0·921 (0·848–0·994)	84·0	92·3	89·6	84·0	92·3	0·840
BMI							
<18·5 (n = 25)	0·897 (0·778–1·00)	100	66·7	84·0	76·5	100	0·867
18·5–23·9 (n = 233)	0·859 (0·809–0·909)	76·3	79·7	78·5	66·3	86·5	0·709
24–27·9 (n = 115)	0·861 (0·793–0·928)	76·5	82·7	80·9	65·0	89·3	0·703
≥28 (n = 17)	0·786 (0·357–1·00)	66·7	100	94·1	100	93·3	0·800
Former or current smoker							
Yes (n = 190)	0·865 (0·813–0·917)	84·0	73·0	77·4	67·0	87·5	0·746
No (n = 200)	0·850 (0·795–0·906)	90·9	66·2	73·0	50·5	95·0	0·649
Education							
Primary school or lower (n = 32)	0·796 (0·592–1·00)	66·7	100	87·5	100	83·3	0·800
Middle school (n = 138)	0·832 (0·764–0·900)	72·9	76·7	75·4	62·5	84·1	0·673
High school (n = 148)	0·889 (0·835–0·943)	76.5	85·6	82·4	73·6	87·4	0·750
Associate's degree (n = 49)	0·885 (0·791–0·979)	100	73·0	79·6	54·5	100	0·706
Bachelor's degree or higher (n = 21)	0·950 (0·843–1·00)	80.0	100	95·2	100	94·1	0·889
CT Apparatus							
SIEMENS (n = 375)	0·860 (0·821–0·898)	73·8	80·0	77·9	65·0	85·8	0·691
GE (n = 15)	0·909 (0·751–1·00)	100	81·8	86·7	66·7	100	0·800
Slice thickness							
≤1 mm (n = 279)	0·850 (0·805–0·896)	66·0	88·4	79·9	77·8	81·0	0·714
1–2 mm (n = 111)	0·877 (0·803–0·951)	83·3	82·8	82·9	57·1	94·7	0·678
Small airway dysfunction							
Yes (n = 193)	0·825 (0·769–0·882)	60·0	89·0	71·0	90·0	57·5	0·720
No (n = 197)	0·901 (0·823–0·979)	80·0	88·8	88·3	27·6	98·8	0·410
Blood EOS count							
≥300/μl (n = 22)	0·822 (0·706–0·938)	78·9	73·0	75·0	60·0	87·1	0·682
<300/μl (n = 156)	0·885 (0·835–0·936)	93·6	69·7	76·9	57·1	96·2	0·710
Chronic respiratory disease							
Yes (n = 86)	0·920 (0·866–0·973)	74·5	94·9	83·7	94·6	75·5	0·833
No (n = 304)	0·829 (0·779–0·879)	69·9	79·6	77·0	56·3	87·6	0·624
Allergic disease							
Yes (n = 88)	0·831 (0·743–0·918)	61·1	92·3	79·5	84·6	77·4	0·710
No (n = 302)	0·868 (0·826–0·910)	76·6	82·7	80·8	66·7	88·7	0·713
Emphysema (CT report)							
Yes (n = 126)	0·835 (0·765–0·905)	71·4	87·3	79·4	84·9	75·3	0·776
No (n = 264)	0·858 (0·808–0·908)	89·6	67·0	72·7	48·0	95·0	0·625
Bronchitis (CT report)							
Yes (n = 21)	0·823 (0·676–0·970)	79·2	87·5	81·3	95·0	58·3	0·864
No (n = 369)	0·858 (0·818–0·897)	71·7	81·0	78·2	61·3	87·2	0·661
Pulmonary nodule (CT report)							
Yes (n = 202)	0·845 (0·787–0·902)	74·6	81·3	79·2	64·4	87·6	0·691
No (n = 188)	0·875 (0·826–0·925)	91·0	66·9	75·5	60·4	93·1	0·726
Bronchiectasis (CT report)							
Yes (n = 46)	0·924 (0·852–0·995)	88·0	81·0	84·8	84·6	85·0	0·863
No (n = 344)	0·844 (0·800–0·888)	71·4	79·9	77·3	61·0	86·4	0·658
Fibrosis (CT report)							
Yes (n = 144)	0·838 (0·772–0·904)	84·0	69·1	74·3	59·2	89·0	0·694
No (n = 246)	0·875 (0·829–0·921)	65·0	93·4	84·1	82·5	84·7	0·727

Abbreviations: AUC, area under the receiver operating characteristic curve; CI, confidence interval; NPV, negative predictive value; PPV, positive predictive value; CT, computed tomography; EOS, eosinophil. The AUC, sensitivity, specificity, accuracy, NPV, PPV and F1 score were calculated using Youden's index.

### Comparison of the AutoCOPD to COPD-SQ

**Fig. 5:**
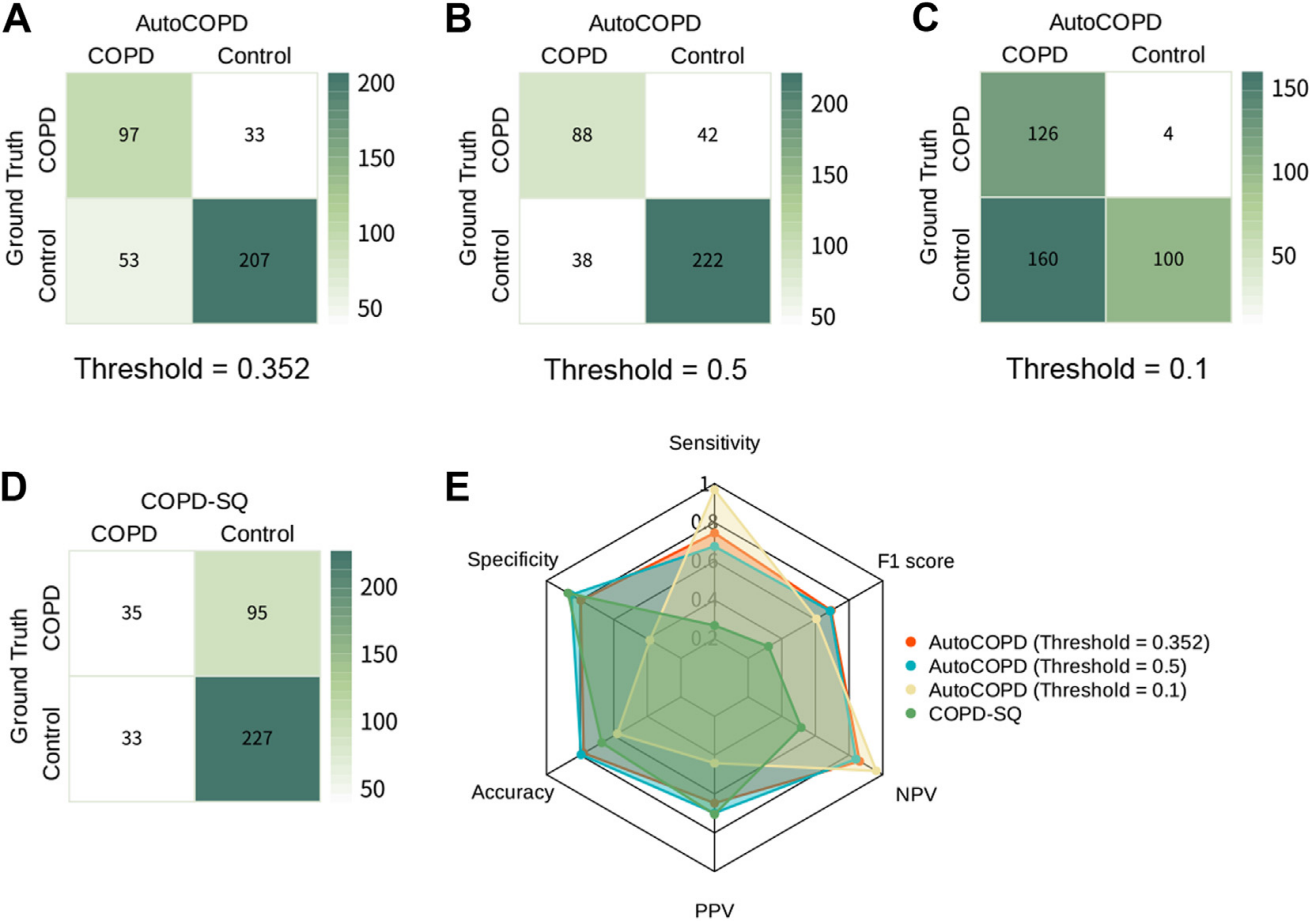
**COPD detection performance using AutoCOPD and COPD-SQ in the internal validation cohort. (A**–**D)** Confusion matrices for AutoCOPD **(A**–**C)** with different thresholds and COPD-SQ **(D)** during prediction of COPD. **(E)** Radar plot for overall performance between AutoCOPD with different thresholds and COPD-SQ evaluated by sensitivity, specificity, accuracy, PPV, NPV, and F1 score. Abbreviations: COPD, chronic obstructive pulmonary disease; COPD-SQ, chronic obstructive pulmonary disease screening questionnaire; PPV, positive predictive value; NPV, negative predictive value.

The confusion matrices for classifier performance in the internal validation cohort using AutoCOPD and COPD-SQ are shown in Fig. 5. Concretely, 97/130 patients with COPD were found with the 0·352 threshold determined by Youden's index. When the threshold was set to 0·5, the number of correctly identified cases mildly decreased to 88/130. At a more extreme threshold of 0·1, the model managed to identify 126/130 patients, but the false positive rate strikingly increased. Instead, COPD-SQ could only screen 35/130 patients with COPD, and its performance in differentiating the control group (227/260) was only slightly better than AutoCOPD with the thresholds of 0·352 (207/260) and 0·5 (222/260). All measurements included sensitivity, specificity, accuracy, NPV, PPV and F1 score were summarized. As shown in the Fig. 5, AutoCOPD was far better than COPD-SQ in terms of the overall performance of case identification. In addition, AutoCOPD had higher F1 scores with different thresholds, indicating a more balanced screening performance.

### Missing data

The overall miss rate for all cohorts ranged from 0% to 12·4%. Specifically, the miss rate in the control group ranged from 0% to 6·52%, and that in the COPD group ranged from 0% to 19·7%. The miss rate was less than 2% for all modeling features, except for the information about the max LD of the 1st generation and average LD of the 4th generation in the external validation cohorts. All the missing values derived from the data of questionnaires, QCT features and blood EOS count. Missing values of the questionnaire information were classified as MAR and NMAR. First, in the scenario of large-scale epidemiological investigation, MAR values occurred for the reason that subjects neglected to answer some questions. In addition, the elimination of incorrect or unreasonable answers due to recall bias resulted in a small quantity of NMAR values. For the missing values of QCT features, they belong to NMAR values which were formed by removing inaccurate data of airway tree annotation or segmentation caused by image resolution, special airway structures (such as heterogeneous bronchi), or algorithm limitations. The deficiency of airway measurements is currently difficult to avoid, but the data of LAA-950 and LAA-910 were non-zero so that the application of AutoCOPD in routine practice as a screening tool would not be significantly constrained. Since the overall miss rate of modeling features was lower than 2% in the derivation cohort, the high accuracy of imputation could be ensured. The reason for high miss rate of blood EOS count was that the subjects were unwilling or did not undergo the examination. However, the feature was not included in the modeling, so it would not affect the proposed AutoCOPD. Detailed information is recorded in Tables S9 and S10.

### Convenient application for routine practice

**Fig. 6:**
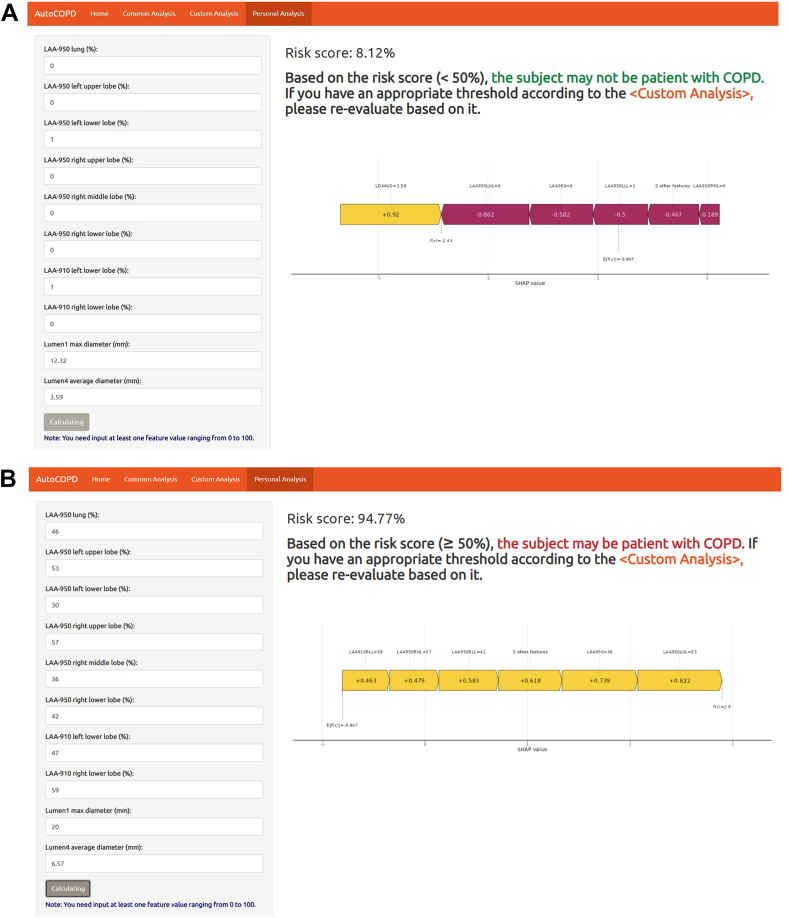
**The risk scores of COPD in two participants were calculatedusing the web application. (A)** The prediction indicated that the probability of the participant developing COPD is 8·12%. PFT showed that FEV1/FVC >0·7. **(B)** The prediction result indicated that the probability of the participant developing COPD is 94·77%. PFT showed that FEV1/FVC <0·7. The force plot indicates the features that contribute to the decision of COPD: the yellow features on the left are pushing the prediction towards the COPD, while the purple features on the right are pushing the prediction towards the non-COPD. Abbreviations: COPD, chronic obstructive pulmonary disease; PFT, pulmonary function test; FEV1/FVC, ratio of forced expiratory volume in 1 s to forced vital capacity.

The AutoCOPD was implemented at https://lwj-lab.shinyapps.io/autocopd/. This web application includes common, custom and personal analyses, which is highly compatible with the pattern of clinical practice. In the common analysis, users can prepare own datasets according to the format of example file and upload them to the application for batch prediction. The predicted score indicates COPD with a probability of ≥50% and non-COPD with a probability of <50%. The custom analysis is aimed at external validation of own dataset, providing the optimal threshold based on Youden's index, as well as showing standard evaluation metrics, ROC curve, calibration curve, and decision curve. All missing data will be imputed before analysis, and users can download the imputed data and predicted results for local research. In the personal prediction, when the actual values of ten features (at least one feature) required for the model are entered and “Calculating” button is clicked, the application will automatically predict the risk of COPD for an individual. Besides, a force plot for the individual will also be displayed to indicate the features that contribute to the decision of COPD: the purple features push the prediction towards “non-COPD”, while the yellow features push the prediction towards “COPD”. An example of its utilization is shown in Fig. 6.

## Discussion

Early diagnosis is crucial for reasonably preventing COPD. We innovatively proposed a predictive model (AutoCOPD) using QCT features extracted from whole-lung inspiratory CT scans to identify COPD in a large multicenter dataset consisting of highly heterogeneous and multi-ethnic participants, and validated its robustness in low- and standard-dose CT images from distinct scanners. Meanwhile, AutoCOPD achieved superior performance compared to multimodal models constructed of comprehensive questionnaire features and structured features from CT reports. To the best of our knowledge, this is the first large-scale, multicenter study that successfully applied a multimodal data fusion and comparison strategy based on QCT, demographics and clinical data in COPD detection in both community and clinical populations.

While multiple case-finding tools for detecting COPD have been proposed in recent decades,^32^ patients are remaining diagnosed based on PFT and clinical phenotype. By contrast, CT images are readily available and contain a wealth of disease information, making them ubiquitously used for diagnosing numerous clinical indications, including pulmonary nodule screening^33^ and interstitial lung disease assessment.^34^ An ever-increasing number of primary care settings in China are leveraging low-dose CT scans to conduct lung cancer screening.^35^ Hence, applying AutoCOPD to perform opportunistic COPD screening via redundant CT images may facilitate the early detection and intervention of COPD beyond the PFT alone, and alleviate the substantial burden on macroeconomy and healthcare systems.^36^

Along with the vigorous development of data preprocessing and artificial intelligence,^37^ CT images tend to be of great value in detecting COPD. González et al.^38^ trained a 2D convolutional neural network using CT scans of COPD genetic epidemiology study cohort at baseline and achieved an AUC of 0·85. Tang et al.^39^ proposed a deep residual network on regions of interest (ROI) pooled from three axial sections, achieving an AUC of 0·886 in the Evaluation of COPD Longitudinally to Identify Predictive Surrogate Endpoints cohort. These studies had preselected ROI or extracted a random subset of CT slices, but the CT manifestations of COPD are diffuse so that focal features cannot capture complete pathological transitions. Thus, the spatial heterogeneity of COPD promotes researchers to focus on changes in the whole lung. Sun et al.^40^ trained an attention-based multi-instance learning (MIL) model using a self-established dataset, and obtained an AUC of 0·934. Xue et al.^41^ developed a two-stage attention MIL model to detect COPD, with an AUC of 0·874 in the external validation set. MIL emphasizes extracting CT voxel to mark subtle lesions.^42^ Nevertheless, these models have yet to provide clear indications for clinical decision-making.

Two recent studies showed excellent performance in detecting COPD using radiomic features extracted from inspiratory low- or standard-dose CT scans.^11^^,^^12^ Although the preprocessing of these features does not involve registration of inspiratory and expiratory CT images, expiratory scans are not widely used in routine practice.^43^ It is worth noting that these features reflecting texture or grayscale may be affected by imaging processing algorithms and effects.^13^ It is also unclear whether these approaches perform well in highly heterogeneous populations rather than smoking-related and clinically diagnosed COPD. AutoCOPD includes predefined QCT features representing lung and airway morphology characteristics. We comprehensively evaluated its detection capability by comparing it with other text-related multimodal models and a typical screening tool, while also analyzing its performance across various subgroups. Furthermore, a user-friendly web application was constructed to help physicians to use model and interpret the results for patients conveniently. Notwithstanding that the image noise on low-dose scans may simulate emphysema^44^ and the segmentation of airways is challenging,^45^ we have successfully validated AutoCOPD in standard-dose scans, ensuring its generalizability in intricate clinical scenarios.

To maximize the transparency of AutoCOPD, we reported the SHAP values of ten features, containing eight CT emphysema and two airway features. Overall, LAA-950 of whole lung had the highest eigenvalue, which highlighted its significance. This finding is consistent with previous study that LAA-950 was validated using histology and universally used for quantifying CT emphysema.^46^ LAA-950 was proved to be importantly associated with the risk of developing COPD.^47^ Moreover, the CT emphysema measured by LAA-910 is significantly associated with FEV1.^48^ Our results showed that LAA-950 of each lobe and LAA-910 in specific regions increased the performance of AutoCOPD, suggesting that the severity of emphysema of COPD varies in different lobes and diverse density masks are also beneficial to evaluation of emphysema.

Two airway dimension measurements were also adopted for constructing AutoCOPD. Average LD of the 4th generation was another top indicator. According to Poiseuille formula, the airway resistance is inversely proportional to the fourth power of the inner diameter.^49^ We showed that the lower the value of average LD of the 4th generation, the higher the risk of developing COPD. Additionally, increased max LD of the 1st generation indicates the heightened risk of COPD. A previous study demonstrated that medium-to large-sized airways of COPD have more mucus plugs,^50^ leading to increased LD in CT images. The symptoms of cough and sputum are mainly associated with the impaired mucus clearance and over-activation of mucus-producing glands distributed in the proximal airways.^51^ It is important to note that, silent and symptomatic mucus plugs are significantly associated with airflow limitation, lower exercise capacity and structural impairment.^52^ As the airway tree extends, the contribution of LD of the 0th–4th generation to the risk of COPD was reversed. This suggests that the mechanism of airflow obstruction caused by large airways is complex, which underscores the necessity of studying those abnormalities on CT scans. In total, increased or decreased LD may occur throughout the entire disease cycle of COPD. It is imperative to integrate multi-omics analyses and experiments to elucidate the driving forces behind these pathological changes, and to assess their potential for detecting pre-COPD.

As radiology resources and practice guidelines vary in medical settings worldwide, it is challenging to propose a universal optimal probability threshold for detecting COPD. For AutoCOPD, a lower threshold might be favored to prevent the omission of potential cases of COPD, while a higher threshold might be more preferable in situations where false positives could give rise to unnecessary interventions. To streamline the clinical practice of COPD identification and enhance the applicability of the model, we set 50% as the diagnostic threshold in the common and personal analysis of the web application. The threshold was in the reasonable range of 0·12–0·66 determined by the DCA. Meanwhile, users were allowed to obtain the optimal threshold for calculating the risk score of COPD according to their actual needs in the custom analysis. It is noteworthy that AutoCOPD could run without the need for inputting complete features, which enables users to make predictions using the LAA-950 feature when lobe-related and airway quantitative indicators are unavailable. Our results corroborated that this scheme achieved acceptable performance, thereby providing confidence for expanding the application scope of the model.

Some limitations should be noted in the current study. First, selection bias was inevitable owing to its retrospective nature. We will conduct a prospective and multicenter study for further validation. Although the proportion of patients with COPD in four centers was imbalanced, which probably triggered some undesirable biases, the performance of AutoCOPD was robust. Second, AutoCOPD was trained in COPD individuals defined by prebronchodilator spirometry and without excluding other comorbidities, which may detract from the validity of the model. However, our model was well-validated in detecting diagnosed COPD. Moderate PPV in the derivation cohort and external validation cohort 4 is also worth noting, which indicates a low proportion of true positive individuals predicted by model. This is closely related to the high proportion (>84%) of subjects with mild-to-moderate airflow limitation in the community and to the long-term smokers (smoking pack-years ≥30) recruited in the NLST trial. On the one hand, manifestations of emphysema are not apparent in the early stage^53^; on the other hand, the degree of emphysema is greater in long-term smokers.^54^ Consequently, LAA measurements were analogous in some participants between the case and control groups, making the prediction tasks challenging. In spite of that, from the DCA, subgroup analyses and the comparison with COPD-SQ, AutoCOPD is conducive to identifying more undiagnosed patients and has latent benefit for screening high-risk populations such as those with SAD^55^ and high blood EOS count.^56^ Third, we did not extract QCT metrics from biphasic CT scans. Despite using these scans can more precisely quantify air trapping in COPD,^10^^,^^57^ they involve a higher radiation dose and more acquisition time, making them improper for routine clinical and physical examination scenarios. Therefore, it is highly valuable to establish a model based on inspiratory CT images for COPD detection. Fourth, some studies emphasized the potential value for COPD risk prediction of additional QCT features, including the lowest 15th percentile of the histogram of attenuation values,^58^ the square root of the bronchial wall area of a hypothetical airway with an internal perimeter of ten mm,^59^ low attenuation cluster^60^ and total airway count.^61^ We plan to upgrade the current model by incorporating these indicators. Fifth, the participants enrolled in this study included higher proportions of men than women, 89% Asian Chinese, and 11% North American populations. However, disparities by country, region, ethnicity, and sex exist in the epidemiology of COPD.^1^^,^^62^ It remains unclear whether AutoCOPD will perform equally well in other populations. Further research is needed to recruit diverse populations to validate the model's performance. Sixth, some questions of COPD-SQ are not mandatory for clinical practice, making it difficult to reflect in electronic health record. For instance, biomass smoke exposure is generally recorded as tobacco exposure, without showing coal or firewood exposure. The question about coughing when not having a cold is a hypothetical question that needs to be circumvented during the consultation. Meanwhile, patients usually seek treatment due to obvious signs or acute exacerbation, resulting in difficulties in recording their stable respiratory symptoms. The data of NLST cohort also lack the above information and could not be scored using COPD-SQ. In light of the retrospective nature, it is infeasible to vindicate whether the predictive performance of AutoCOPD is also superior to that of COPD-SQ within the external validation cohorts. Admittedly, the comparison with COPD-SQ only in the internal validation cohort may unfairly favour the derived AutoCOPD. We will steer clear of this constraint in the future prospective trial. Finally, we did not evaluate the performance of AutoCOPD on other low-dose CT datasets from China, but it was applicable to the NLST cohort when validated. Our future work will collect more low-dose CT scans to assess the model.

In conclusion, AutoCOPD constructed by only ten QCT features representing parenchymal structures and airway morphology from whole-lung inspiratory CT scans can accurately detect heterogeneous COPD. It may also serve as a feasible tool for early COPD detection, provide concrete indicators for helping better characterize early COPD and improve the management strategy of specific individuals.

## Contributors

WL, NZ, ZL, YL, and JW conceived the study and designed the experiments. FL, CL, JX, YL, XZ, JH, RS, and JZ performed the experiments. FL performed the data analyses, established the machine learning models, and drafted the manuscript. RS edited the manuscript. CL and JX contributed substantially to the development of QCT measurement system. FL, WLia, QZ, HC, JZ, YM, QM, JX, YL, LY, XW, LW, and RH contributed to data collection. TX, ZZ, and RS contributed to data analyses and visualization. FL, ZZ, CL, and WL have accessed and verified the underlying data. WL, NZ, ZZ, JW, XZ, ZL, and YL oversaw the completion of this study and determined the final manuscript. All authors read and approved the final version of the manuscript.

## Data sharing statement

Deidentified data of NLST cohort analyzed during the study were acquired through data transfer agreement with NCI. Requests for data will require approval from NCI and the signing of data access agreements. Other data analyzed and the codes used during the current study are available from the corresponding author on reasonable request.

## Declaration of interests

CL is a senior engineer of Neusoft Medical Systems, a leading company of global information technology, product, and solution. WL received free access to the *NeuLungCare*–*QA* software for CT images analysis provided by Neusoft Medical Systems, and received free technical support from Guangzhou Tianpeng Computer Technology Co., Ltd. FL received free access to NCI's data collected by NLST. All other authors do not have any potential conflicts of interest to declare.
