# Supplementary material for: AutoCOPD–A novel and practical machine learning model for COPD detection using whole-lung inspiratory quantitative CT measurements: a retrospective, multicenter study
Source: eClinicalMedicine. 2025 Apr 3;82:103166. doi: 10.1016/j.eclinm.2025.103166 (PMC12002883; doi:10.1016/j.eclinm.2025.103166)
Supplement: Supplementary Tables and Figures [file mmc1.docx]

**Table of contents**

**Table S1: CT acquisition protocols of derivation and external validation cohorts. 2**

**Table S2: Data dictionary of questionnaire, QCT and CT report features 5**

**Table S3: QCT measurements of lung parenchyma and airway.. 16**

**Table S4: Detailed information of primary packages for statistical analysis, machine learning, and**

**web application construction. 17**

**Table S5: Questionnaire, QCT measurements and CT report characteristics in the overall derivation**

**cohort and split cohorts. 18**

**Table S6: Demographic characteristics for the external validation cohorts. 29**

**Table S7: Selected features of seven schemes. 36**

**Table S8: COPD detection performance for AutoCOPD in various subgroups of the external**

**validation cohorts. 38**

**Table S9: Missingness of derivation cohort. 45**

**Table S10: Missingness of external validation cohorts. 50**

**Figure S1: Graphical abstract. 53**

**Figure S2: QCT measurements of emphysema and airway tree. 54**

| **Cohorts** | **Scanning equipment** | **Tube voltage (kV)** | **Tube current (mAs)** | **Pitch** | **Slice thickness (mm)** | **Matrix** | **FOV** | **Type** |
| --- | --- | --- | --- | --- | --- | --- | --- | --- |
| **Derivation cohort** | SIEMENS Perspective | 110 | AEC | 0·85 | 1, 2 | 512×512 | Lung | Low-dose |
|  | SIEMENS SOMATOM Definition AS+ | 100,120 | 43, 45, 50, 52, 54, 72 | 0·9 | 1, 2 | 512×512 |  |  |
|  | GE MEDICAL SYSTEMS Revolution CT | 100 | 50, 60 | 0·9921875 | 1, 2 | 512×512 |  |  |
| **External**  **validation cohort 1** | SIEMENS Perspective | 110, 130 | AEC | 0·45, 0·55, 0·6, 0·65, 0·7, 0·75, 0·8, 0·85, 1, 1·1, 1·3 | 0·75, 1, 2 | 512×512 | Lung | Standard-dose |
|  | SIEMENS SOMATOM Definition AS+ | 100, 120, 140 | AEC | 0·6, 0·65, 0·8, 0·9, 1, 1·1, 1·2, 1·5 | 0·75, 1, 2 | 512×512 |  |  |
|  | GE MEDICAL SYSTEMS Revolution CT | 100, 120, 140 | AEC | 0·9921875 | 0·82, 1, 2 | 512×512 |  |  |
|  | GE MEDICAL SYSTEMS Revolution Apex | 80, 100, 120, 140 | AEC | 0·9921875 | 0·679688, 0·726563, 0·808594, 0·818359, 1, 2 | 512×512 |  |  |
|  | NMS NeuViz 64 In | 120 | AEC | 1·3 | 1 | 512×512 |  |  |
|  | NMS NeuViz 128 | 120 | AEC | 0·9, 1 | 1 | 512×512 |  |  |
|  | NMS NeuViz Extra | 120 | 252, 270, 337 | 0·9 | 1 | 512×512 |  |  |
|  | NMS NeuViz Epoch | 120, 230 | AEC | 0·8, 0·9, 1 | 1, 1·1, 2 | 512×512 |  |  |
|  | TOSHIBA Aquilion | 120 | 100, 150, 190, 200 | none | 1, 2 | 512×512 |  |  |
| **External**  **validation cohort 2** | SIEMENS SOMATOM Drive | 120 | AEC | 0·7, 0·8, 1 | 1, 2 | 512×512 | Lung | Standard-dose |
|  | Siemens Healthineers SOMATOM go.Fit | 100, 110, 120 | AEC | 0·8, 1·2 | 1, 2 | 512×512 |  |  |
|  | UIH uCT 510 | 100, 120 | AEC | 1·0625, 1·1875, 1·3125 | 1, 2 | 512×512 |  |  |
|  | Philips MX 16-slice | 120 | 224 | 0·8631 | 1·5, 2 | 512×512 |  |  |
|  | Philips Brilliance 64 | 120 | 279, 287 | none | 1 | 512×512 |  |  |
|  | Philips iCT 256 | 120, 140 | AEC | 0·603, 0·763, 0·874, 0·875, 0·914, 0·925, 0·941, 0·992, 1·1875, 2 | 0·9, 1, 2 | 512×512 |  |  |
|  | Philips Ingenuity CT | 120 | 320, 446, 455 | 0·64, 1·2, 1·238, 1·477 | 1 | 512×512 |  |  |
| **External**  **validation cohort 3** | SIEMENS SOMATOM Force | 70, 90, 100, 110 | AEC | 0·6, 1·2, 1·9, 2 | 0·5, 0·6, 0·75, 1, 1·5 | 512×512 | Lung | Standard-dose |
|  | SIEMENS SOMATOM go.Top | 100, 120 | AEC | 0·6 | 1·5 |  |  |  |
|  | SIEMENS SOMATOM Definition Flash | 100, 120, 140 | AEC | 0·35, 0·45, 0·5, 0·55, 0·6, 0·65, 0·7, 0·75, 0·8, 0·85, 1, 1·15, 1·2, 1·55 | 0·5, 0·6, 0·75, 1, 1·5, 2 | 512×512 |  |  |
|  | SIEMENS Healthineers SOMATOM go.Top | 100 | AEC | 0·6, 1·1, 1·2, 1·5 | 0·6, 1·5 | 512×512 |  |  |
|  | GE MEDICAL SYSTEMS Revolution CT | 100 | AEC | 0·9921875 | 1·25 | 512×512 |  |  |
|  | GE MEDICAL SYSTEMS LightSpeed VCT | 120 | 150, 350 | 0·984375 | 1·25, 2 | 512×512 |  |  |
|  | GE MEDICAL SYSTEMS BrightSpeed | 120 | AEC | 1·375 | 1·25 | 512×512 |  |  |
|  | UIH uCT 528 | 100 | AEC | 1·175 | 1 | 512×512 |  |  |
|  | UIH uCT 780 | 100, 120, 140 | AEC | 0·975, 0·9875, 1·0875, 1·2125 | 1, 1·2 | 512×512 |  |  |
|  | Anke ANATOM 64 Precision | 100 | AEC | 1, 1·5 | 1·25 | 512×512 |  |  |
| **External**  **validation cohort 4** | SIEMENS Sensation 4 | 120 | 80, 160 | none | 2 | 512×512 | Lung | Low-dose |
|  | SIEMENS Sensation 16 | 120 | AEC | none | 1, 2 | 512×512 |  |  |
|  | SIEMENS Sensation 64 | 120 | 75 | none | 2 | 512×512 |  |  |
|  | SIEMENS Volume Zoom | 120, 140 | AEC | none | 1, 2 | 512×512 |  |  |
|  | GE MEDICAL SYSTEMS LightSpeed16 | 120 | 80, 120, 140 | none | 1·25 | 512×512 |  |  |
|  | GE MEDICAL SYSTEMS LightSpeed Pro 16 | 120 | 80, 140, 160 | none | 1·25 | 512×512 |  |  |
|  | GE MEDICAL SYSTEMS LightSpeed Ultra | 120 | 80, 100, 110, 115, 120, 140 | none | 1·25, 2·5 | 512×512 |  |  |
|  | GE MEDICAL SYSTEMS LightSpeed QX/i | 120 | 50 | none | 2·5 | 512×512 |  |  |
|  | Philips Mx8000 | 120 | 120, 150, 210 | none | 1·3 | 512×512 |  |  |
|  | Philips Mx8000 IDT | 120 | AEC | none | 2 | 512×512 |  |  |
|  | TOSHIBA Aquilion | 120 | 80, 100, 120, 150, 160 | none, 1·4375, 1·5 | 2, 2·5 |  |  |  |
| Abbreviations: AEC, automatic exposure control; FOV, field of view. | | | | | | | |  |

**Table S1: CT acquisition protocols of derivation and external validation cohorts.**

| **Features** | **Label** | **Description** | **Format text** |
| --- | --- | --- | --- |
| AGEY | Age | Age in years. | Numeric |
| AGE | Age group | Age in years (interval). | 1 = “35–49”  2 = “50–59”  3 = “60–69”  4 = “70–80” |
| SEX | Sex | Sex is defined according to physiological characteristics of male or female. | 1 = “Female”  2 = “Male” |
| BMI | Body mass index | BMI is defined as weight divided by height squared (kg/m^2^). | 1 = “≥ 28”  2 = “24–27·9”  3 = “18·5–23·9”  4 = “< 18·5” |
| SMING | Smoking | Participant smoked more than 100 cigarettes at the start of the survey. | 1 = “No”  2 = “Yes” |
| SMINGPY | Smoking pack-years | Pack years, calculated as: total years smoked x cigarettes per day/20. | 1 = “0”  2 = “1–14”  3 = “15–30”  4 = “≥ 30” |
| QSMING | Quit smoking | Participant has ceased smoking at least one year at the start of the survey. | 1 = “Non-smoker”  2 = “Yes”  3 = “No” |
| QSMING15Y | Quit smoking over 15 years | Participant has ceased smoking over 15 years at the start of the survey. | 1 = “Non-smoker”  2 = “Yes”  3 = “No”  4 = “Current smoker” |
| SECSMING | Secondhand smoke exposure | Participant (non-smoker) was exposed to secondhand smoke for over 15 minutes on at least one day per week. | 1 = “No”  2 = “Yes”  3 = “Former smoker”  4 = “Current smoker” |
| EDU | Education | Educational level completed. | 1 = “Bachelor's degree or higher”  2 = “Associate's degree”  3 = “High school”  4 = “Middle school”  5 = “Primary school or lower” |
| MARRIAGE | Marital status | Participant who was first married or remarried were defined as “Married” individual, while who was unmarried, cohabiting, divorced or widowed were defined as “Ever or never married” individual. | 1 = “Married”  2 = “Ever or never married” |
| OCCUPE | Occupational exposure | Exposure includes inorganic dust, organic dust, vapours, harmful exhaust gases, fumes, pesticides, aromatic solvents, chlorinated solvents, and metals. | 1 = “No”  2 = “Yes” |
| KITVENT | Kitchen ventilator | / | 1 = “No”  2 = “Yes” |
| VENT | Home ventilation | / | 1 = “Good”  2 = “General”  3 = “Bad” |
| DECOR | Indoor renovation within ten years | Indoor places include house and office. | 1 = “No”  2 = “Yes” |
| INCENSE | Incense burning | / | 1 = “Yearly or less”  2 = “Monthly”  3 = “Weekly or more” |
| ICOOK | Self-cooking | / | 1 = “Yearly or less”  2 = “Monthly”  3 = “Weekly or more” |
| BIOFUEL | Cooking fuel type within ten years | / | 1 = “LPG or gas”  2 = “Coal or firewood” |
| BIOFUELCHI | Cooking fuel type in childhood | / | 1 = “LPG or gas”  2 = “Coal or firewood” |
| DRINK | Alcohol drinking | / | 1 = “Yearly or less”  2 = “Monthly”  3 = “Weekly or more” |
| TEA | Tea drinking | / | 1 = “Weekly or more”  2 = “Monthly”  3 = “Yearly or less” |
| PICKLE | Pickled food | / | 1 = “Yearly or less”  2 = “Monthly”  3 = “Weekly or more” |
| BEAN | Bean food | / | 1 = “Weekly or more”  2 = “Monthly”  3 = “Yearly or less” |
| EXERCISE | Exercise | / | 1 = “Weekly or more”  2 = “Monthly”  3 = “Yearly or less” |
| PET | Pet raising | / | 1 = “No”  2 = “Yes” |
| SLEEP | Total sleep time | Unit: hour. | Numeric |
| TRAUMA | Mental trauma | Participant suffered mental trauma containing experiencing serious illness or death of family members, family breakdown, significant financial burden, unemployment, serious physical injury, and violent intimidation. | 1 = “No”  2 = “Yes” |
| DEPRESS | Depressed mood | Participant had been in a depressed state for more than six months. | 1 = “No”  2 = “Yes” |
| ALLER | Ambient air temperature-related allergy | / | 1 = “No”  2 = “Yes” |
| HOSPINTEN | Hospitalized before the age of ten | Participant hospitalized before the age of ten due to the respiratory diseases. | 1 = “No”  2 = “Yes” |
| COUGH | Cough | Participant coughed frequently when not having a cold. | 1 = “No”  2 = “Yes” |
| PHLEGM | Produce phlegm | Participant produced phlegm frequently when not having a cold. | 1 = “No”  2 = “Yes” |
| TACHYPNEA | Tachypnea | / | 1 = “No”  2 = “Hurrying on flat ground or walking up a small slope”  3 = “Walking at own pace on flat ground” |
| FHRESPIRDIS | Family history of respiratory disease | Parents, brothers, sisters or children of participant suffered from chronic bronchitis, emphysema or COPD. | 1 = “No”  2 = “Yes” |
| RESPIRDIS | Chronic respiratory disease | Participant suffered from chronic bronchitis, emphysema, asthma, bronchiectasia, interstitial pneumonia, obstructive sleep apnoea/hypopnoea syndrome, pulmonary hypertension, pulmonary tuberculosis, pneumoconiosis, asbestosis, sarcoidosis as self-reported at the start of the survey. | 1 = “No”  2 = “Yes” |
| HYPRESS | Hypertension | Hypertension: Ever diagnosed prior to survey. | 1 = “No”  2 = “Yes” |
| DIABET | Diabetes | Diabetes: Ever diagnosed prior to survey. | 1 = “No”  2 = “Yes” |
| HEARTDIS | Heart disease | Heart disease: Ever diagnosed prior to survey. | 1 = “No”  2 = “Yes” |
| STROKE | Stroke | Stroke: Ever diagnosed prior to survey. | 1 = “No”  2 = “Yes” |
| ALLERDIS | Allergic disease | Allergic disease: Ever diagnosed prior to survey. | 1 = “No”  2 = “Yes” |
| LAA950 | LAA-950 of lung | Unit: %. | Numeric |
| LAA950LUL | LAA-950 of left upper lobe | Unit: %. | Numeric |
| LAA950LLL | LAA-950 of left lower lobe | Unit: %. | Numeric |
| LAA950RUL | LAA-950 of right upper lobe | Unit: %. | Numeric |
| LAA950RML | LAA-950 of right middle lobe | Unit: %. | Numeric |
| LAA950RLL | LAA-950 of right lower lobe | Unit: %. | Numeric |
| LAA910 | LAA-910 of lung | Unit: %. | Numeric |
| LAA910LUL | LAA-910 of left upper lobe | Unit: %. | Numeric |
| LAA910LLL | LAA-910 of left lower lobe | Unit: %. | Numeric |
| LAA910RUL | LAA-910 of right upper lobe | Unit: %. | Numeric |
| LAA910RML | LAA-910 of right middle lobe | Unit: %. | Numeric |
| LAA910RLL | LAA-910 of right lower lobe | Unit: %. | Numeric |
| WA0 | %WA of 0th generation | Unit: %. | Numeric |
| WA1 | %WA of 1st generation | Unit: %. | Numeric |
| WA2 | %WA of 2nd generation | Unit: %. | Numeric |
| WA3 | %WA of 3rd generation | Unit: %. | Numeric |
| WA4 | %WA of 4th generation | Unit: %. | Numeric |
| WT0AVG | Average WT of 0th generation | Unit: mm. | Numeric |
| WT1AVG | Average WT of 1st generation | Unit: mm. | Numeric |
| WT2AVG | Average WT of 2nd generation | Unit: mm. | Numeric |
| WT3AVG | Average WT of 3rd generation | Unit: mm. | Numeric |
| WT4AVG | Average WT of 4th generation | Unit: mm. | Numeric |
| WT0MAX | Max WT of 0th generation | Unit: mm. | Numeric |
| WT1MAX | Max WT of 1st generation | Unit: mm. | Numeric |
| WT2MAX | Max WT of 2nd generation | Unit: mm. | Numeric |
| WT3MAX | Max WT of 3rd generation | Unit: mm. | Numeric |
| WT4MAX | Max WT of 4th generation | Unit: mm. | Numeric |
| WT0MIN | Min WT of 0th generation | Unit: mm. | Numeric |
| WT1MIN | Min WT of 1st generation | Unit: mm. | Numeric |
| WT2MIN | Min WT of 2nd generation | Unit: mm. | Numeric |
| WT3MIN | Min WT of 3rd generation | Unit: mm. | Numeric |
| WT4MIN | Min WT of 4th generation | Unit: mm. | Numeric |
| LD0AVG | Average LD of 0th generation | Unit: mm. | Numeric |
| LD1AVG | Average LD of 1st generation | Unit: mm. | Numeric |
| LD2AVG | Average LD of 2nd generation | Unit: mm. | Numeric |
| LD3AVG | Average LD of 3rd generation | Unit: mm. | Numeric |
| LD4AVG | Average LD of 4th generation | Unit: mm. | Numeric |
| LD0MAX | Max LD of 0th generation | Unit: mm. | Numeric |
| LD1MAX | Max LD of 1st generation | Unit: mm. | Numeric |
| LD2MAX | Max LD of 2nd generation | Unit: mm. | Numeric |
| LD3MAX | Max LD of 3rd generation | Unit: mm. | Numeric |
| LD4MAX | Max LD of 4th generation | Unit: mm. | Numeric |
| LD0MIN | Min LD of 0th generation | Unit: mm. | Numeric |
| LD1MIN | Min LD of 1st generation | Unit: mm. | Numeric |
| LD2MIN | Min LD of 2nd generation | Unit: mm. | Numeric |
| LD3MIN | Min LD of 3rd generation | Unit: mm. | Numeric |
| LD4MIN | Min LD of 4th generation | Unit: mm. | Numeric |
| EMPHY | Emphysema of lung | The “Diagnosis” section of CT report included emphysema and pulmonary bullae of lung. | 1 = “No”  2 = “Yes” |
| EMPHYLL | Emphysema of left lung | The “Diagnosis” section of CT report included emphysema and pulmonary bullae of left lung. | 1 = “No”  2 = “Yes” |
| EMPHYLUL | Emphysema of left upper lobe | The “Diagnosis” section of CT report included emphysema and pulmonary bullae of left upper lobe. | 1 = “No”  2 = “Yes” |
| EMPHYLLL | Emphysema of left lower lobe | The “Diagnosis” section of CT report included emphysema and pulmonary bullae of left lower lobe. | 1 = “No”  2 = “Yes” |
| EMPHYRL | Emphysema of right lung | The “Diagnosis” section of CT report included emphysema and pulmonary bullae of right lung. | 1 = “No”  2 = “Yes” |
| EMPHYRUL | Emphysema of right upper lobe | The “Diagnosis” section of CT report included emphysema and pulmonary bullae of right upper lobe. | 1 = “No”  2 = “Yes” |
| EMPHYRML | Emphysema of right middle lobe | The “Diagnosis” section of CT report included emphysema and pulmonary bullae of right middle lobe. | 1 = “No”  2 = “Yes” |
| EMPHYRLL | Emphysema of right lower lobe | The “Diagnosis” section of CT report included emphysema and pulmonary bullae of right lower lobe. | 1 = “No”  2 = “Yes” |
| BROCHI | Bronchitis of lung | The “Diagnosis” section of CT report included bronchitis, bronchiolitis, and small airway inflammation of lung. | 1 = “No”  2 = “Yes” |
| BROCHILL | Bronchitis of left lung | The “Diagnosis” section of CT report included bronchitis, bronchiolitis, and small airway inflammation of left lung. | 1 = “No”  2 = “Yes” |
| BROCHILUL | Bronchitis of left upper lobe | The “Diagnosis” section of CT report included bronchitis, bronchiolitis, and small airway inflammation of left upper lobe. | 1 = “No”  2 = “Yes” |
| BROCHILLL | Bronchitis of left lower lobe | The “Diagnosis” section of CT report included bronchitis, bronchiolitis, and small airway inflammation of left lower lobe. | 1 = “No”  2 = “Yes” |
| BROCHIRL | Bronchitis of right lung | The “Diagnosis” section of CT report included bronchitis, bronchiolitis, and small airway inflammation of right lung. | 1 = “No”  2 = “Yes” |
| BROCHIRUL | Bronchitis of right upper lobe | The “Diagnosis” section of CT report included bronchitis, bronchiolitis, and small airway inflammation of right upper lobe. | 1 = “No”  2 = “Yes” |
| BROCHIRML | Bronchitis of right middle lobe | The “Diagnosis” section of CT report included bronchitis, bronchiolitis, and small airway inflammation of right middle lobe. | 1 = “No”  2 = “Yes” |
| BROCHIRLL | Bronchitis of right lower lobe | The “Diagnosis” section of CT report included bronchitis, bronchiolitis, and small airway inflammation of right lower lobe. | 1 = “No”  2 = “Yes” |
| NODULE | Pulmonary nodule of lung | The “Diagnosis” section of CT report included granuloma and inflammatory nodule of lung. | 1 = “No”  2 = “Yes” |
| NODULELL | Pulmonary nodule of left lung | The “Diagnosis” section of CT report included granuloma and inflammatory nodule of left lung. | 1 = “No”  2 = “Yes” |
| NODULELUL | Pulmonary nodule of left upper lobe | The “Diagnosis” section of CT report included granuloma and inflammatory nodule of left upper lobe. | 1 = “No”  2 = “Yes” |
| NODULELLL | Pulmonary nodule of left lower lobe | The “Diagnosis” section of CT report included granuloma and inflammatory nodule of left lower lobe. | 1 = “No”  2 = “Yes” |
| NODULERL | Pulmonary nodule of right lung | The “Diagnosis” section of CT report included granuloma and inflammatory nodule of right lung. | 1 = “No”  2 = “Yes” |
| NODULERUL | Pulmonary nodule of right upper lobe | The “Diagnosis” section of CT report included granuloma and inflammatory nodule of right upper lobe. | 1 = “No”  2 = “Yes” |
| NODULERML | Pulmonary nodule of right middle lobe | The “Diagnosis” section of CT report included granuloma and inflammatory nodule of right middle lobe. | 1 = “No”  2 = “Yes” |
| NODULERLL | Pulmonary nodule of right lower lobe | The “Diagnosis” section of CT report included granuloma and inflammatory nodule of right lower lobe. | 1 = “No”  2 = “Yes” |
| BRONCHIECT | Bronchiectasia of lung | The “Diagnosis” section of CT report included bronchiectasia of lung. | 1 = “No”  2 = “Yes” |
| BRONCHIECTLL | Bronchiectasia of left lung | The “Diagnosis” section of CT report included bronchiectasia of left lung. | 1 = “No”  2 = “Yes” |
| BRONCHIECTLUL | Bronchiectasia of left upper lobe | The “Diagnosis” section of CT report included bronchiectasia of left upper lobe. | 1 = “No”  2 = “Yes” |
| BRONCHIECTLLL | Bronchiectasia of left lower lobe | The “Diagnosis” section of CT report included bronchiectasia of left lower lobe. | 1 = “No”  2 = “Yes” |
| BRONCHIECTRL | Bronchiectasia of right lung | The “Diagnosis” section of CT report included bronchiectasia of right lung. | 1 = “No”  2 = “Yes” |
| BRONCHIECTRUL | Bronchiectasia of right upper lobe | The “Diagnosis” section of CT report included bronchiectasia of right upper lobe. | 1 = “No”  2 = “Yes” |
| BRONCHIECTRML | Bronchiectasia of right middle lobe | The “Diagnosis” section of CT report included bronchiectasia of right middle lobe. | 1 = “No”  2 = “Yes” |
| BRONCHIECTRLL | Bronchiectasia of right lower lobe | The “Diagnosis” section of CT report included bronchiectasia of right lower lobe. | 1 = “No”  2 = “Yes” |
| FIBRO | Fibrosis of lung | The “Diagnosis” section of CT report included fibrosis and fibrous foci of lung. | 1 = “No”  2 = “Yes” |
| FIBROLL | Fibrosis of left lung | The “Diagnosis” section of CT report included fibrosis and fibrous foci of left lung. | 1 = “No”  2 = “Yes” |
| FIBROLUL | Fibrosis of left upper lobe | The “Diagnosis” section of CT report included fibrosis and fibrous foci of left upper lobe. | 1 = “No”  2 = “Yes” |
| FIBROLLL | Fibrosis of left lower lobe | The “Diagnosis” section of CT report included fibrosis and fibrous foci of left lower lobe. | 1 = “No”  2 = “Yes” |
| FIBRORL | Fibrosis of right lung | The “Diagnosis” section of CT report included fibrosis and fibrous foci of right lung. | 1 = “No”  2 = “Yes” |
| FIBRORUL | Fibrosis of right upper lobe | The “Diagnosis” section of CT report included fibrosis and fibrous foci of right upper lobe. | 1 = “No”  2 = “Yes” |
| FIBRORML | Fibrosis of right middle lobe | The “Diagnosis” section of CT report included fibrosis and fibrous foci of right middle lobe. | 1 = “No”  2 = “Yes” |
| FIBRORLL | Fibrosis of right lower lobe | The “Diagnosis” section of CT report included fibrosis and fibrous foci of right lower lobe. | 1 = “No”  2 = “Yes” |
| INFLAM | Inflammation of lung | The “Diagnosis” section of CT report included chronic inflammation of lung. | 1 = “No”  2 = “Yes” |
| INFLAMLL | Inflammation of left lung | The “Diagnosis” section of CT report included chronic inflammation of left lung. | 1 = “No”  2 = “Yes” |
| INFLAMLUL | Inflammation of left upper lobe | The “Diagnosis” section of CT report included chronic inflammation of left upper lobe. | 1 = “No”  2 = “Yes” |
| INFLAMLLL | Inflammation of left lower lobe | The “Diagnosis” section of CT report included chronic inflammation of left lower lobe. | 1 = “No”  2 = “Yes” |
| INFLAMRL | Inflammation of right lung | The “Diagnosis” section of CT report included chronic inflammation of right lung. | 1 = “No”  2 = “Yes” |
| INFLAMRUL | Inflammation of right upper lobe | The “Diagnosis” section of CT report included chronic inflammation of right upper lobe. | 1 = “No”  2 = “Yes” |
| INFLAMRML | Inflammation of right middle lobe | The “Diagnosis” section of CT report included chronic inflammation of right middle lobe. | 1 = “No”  2 = “Yes” |
| INFLAMRLL | Inflammation of right lower lobe | The “Diagnosis” section of CT report included chronic inflammation of right lower lobe. | 1 = “No”  2 = “Yes” |
| CALCI | Calcification of lung | The “Diagnosis” section of CT report included calcification of lung. | 1 = “No”  2 = “Yes” |
| CALCILL | Calcification of left lung | The “Diagnosis” section of CT report included calcification of left lung. | 1 = “No”  2 = “Yes” |
| CALCILUL | Calcification of left upper lobe | The “Diagnosis” section of CT report included calcification of left upper lobe. | 1 = “No”  2 = “Yes” |
| CALCILLL | Calcification of left lower lobe | The “Diagnosis” section of CT report included calcification of left lower lobe. | 1 = “No”  2 = “Yes” |
| CALCIRL | Calcification of right lung | The “Diagnosis” section of CT report included calcification of right lung. | 1 = “No”  2 = “Yes” |
| CALCIRUL | Calcification of right upper lobe | The “Diagnosis” section of CT report included calcification of right upper lobe. | 1 = “No”  2 = “Yes” |
| CALCIRML | Calcification of right middle lobe | The “Diagnosis” section of CT report included calcification of right middle lobe. | 1 = “No”  2 = “Yes” |
| CALCIRLL | Calcification of right lower lobe | The “Diagnosis” section of CT report included calcification of right lower lobe. | 1 = “No”  2 = “Yes” |
| TB | Tuberculosis of lung | The “Diagnosis” section of CT report included tuberculosis of lung. | 1 = “No”  2 = “Yes” |
| TBLL | Tuberculosis of left lung | The “Diagnosis” section of CT report included tuberculosis of left lung. | 1 = “No”  2 = “Yes” |
| TBLUL | Tuberculosis of left upper lobe | The “Diagnosis” section of CT report included tuberculosis of left upper lobe. | 1 = “No”  2 = “Yes” |
| TBLLL | Tuberculosis of left lower lobe | The “Diagnosis” section of CT report included tuberculosis of left lower lobe. | 1 = “No”  2 = “Yes” |
| TBRL | Tuberculosis of right lung | The “Diagnosis” section of CT report included tuberculosis of right lung. | 1 = “No”  2 = “Yes” |
| TBRUL | Tuberculosis of right upper lobe | The “Diagnosis” section of CT report included tuberculosis of right upper lobe. | 1 = “No”  2 = “Yes” |
| TBRML | Tuberculosis of right middle lobe | The “Diagnosis” section of CT report included tuberculosis of right middle lobe. | 1 = “No”  2 = “Yes” |
| TBRLL | Tuberculosis of right lower lobe | The “Diagnosis” section of CT report included tuberculosis of right lower lobe. | 1 = “No”  2 = “Yes” |
| PLEUTHICK | Pleural thickness | The “Diagnosis” section of CT report included pleural thickness of lung. | 1 = “No”  2 = “Yes” |
| Abbreviations: LPG: liquefied petroleum gas; COPD, chronic obstructive pulmonary disease; LAA-950, low-attenuating area below -950 Hounsfield Units; LAA-910, low-attenuating area below -910 Hounsfield Units; %WA, airway wall area percentage; WT, wall thickness; LD, lumen diameter; CT, computed tomography. | | | |

**Table S2: Data dictionary of questionnaire, QCT and CT report features.**

| **Measurements** | **Whole Lung** | **Region** | | | | | | | | | |
| --- | --- | --- | --- | --- | --- | --- | --- | --- | --- | --- | --- |
|  |  | **Left upper lobe** | **Left lower lobe** | **Right upper lobe** | **Right middle lobe** | **Right lower lobe** | **0th generation** | **1st generation** | **2nd generation** | **3rd generation** | **4th generation** |
| LAA-950 | √ | √ | √ | √ | √ | √ | × | × | × | × | × |
| LAA-910 | √ | √ | √ | √ | √ | √ | × | × | × | × | × |
| %WA | × | × | × | × | × | × | √ | √ | √ | √ | √ |
| Average WT | × | × | × | × | × | × | √ | √ | √ | √ | √ |
| Max WT | × | × | × | × | × | × | √ | √ | √ | √ | √ |
| Min WT | × | × | × | × | × | × | √ | √ | √ | √ | √ |
| Average LD | × | × | × | × | × | × | √ | √ | √ | √ | √ |
| Max LD | × | × | × | × | × | × | √ | √ | √ | √ | √ |
| Min LD | × | × | × | × | × | × | √ | √ | √ | √ | √ |
| Abbreviations: LAA-950, low-attenuating area below -950 Hounsfield Units; LAA-910, low-attenuating area below -910 Hounsfield Units; %WA, airway wall area percentage; WT, wall thickness; LD, lumen diameter. | | | | | | | | | | | |

**Table S3: QCT measurements of lung parenchyma and airway.**

| **Package** | **Version number** | **Function** | **Application** |
| --- | --- | --- | --- |
| caret | 6·0-94 | createDataPartition() | Randomly divide the derivation cohort into training and internal validation cohorts at a ratio of 8:2 |
|  |  | createFolds() | Split the data into ten folds |
|  |  | nearZeroVar() | Exclude variables with zero variance and near-zero variance |
| missForest | 1·5 | missForest() | Impute missing data |
| fBasics | 4032·96 | shapiroTest() | Test the normality of independent variables |
| tableone | 0·13·2 | CreateTableOne() | Describe baseline characteristics |
| xgboost | 1·7·7·1 | xgboost() | Train XGBoost models |
|  |  | xgb.cv() | The cross validation function of XGBoost |
| rBayesianOptimization | 1·2·1 | BayesianOptimization() | Bayesian optimization of hyperparameters. |
| shapviz | 0·9·3 | shapviz() | Calculate the SHAP values of features |
| pROC | 1·18·5 | roc() | Construct ROC curves |
|  |  | auc() | Computes the numeric value of AUC with the trapezoidal rule |
|  |  | ci() | Computes the CI of a ROC curve (Default: the 95% CI are computed with 2000 stratified bootstrap replicates) |
|  |  | coords() | Return the coordinates of ROC curves |
|  |  | roc.test() | Compares two correlated (or paired) or uncorrelated (unpaired) ROC curves |
| rmda | 1·6 | decision_curve() | Evaluate the value of using AutoCOPD to decide treatment or intervention (versus no treatment or intervention) |
| stats | 4·4·0 | lowess() | Perform the computations for the LOWESS smoother |
| ResourceSelection | 0·3-6 | hoslem.test() | Hosmer-Lemeshow goodness of fit test |
| shiny | 1·8·0 | shinyApp(ui, server) | Web application framework |
| shinythemes | 1·2·0 | shinytheme() | Themes for shiny |
| Abbreviations: XGBoost, eXtreme gradient boosting; SHAP, SHapley Additive exPlanation; ROC, receiver operating characteristic curve; AUC, area under the receiver operating characteristic curve; CI, confidence interval. | | | |

**Table S4: Detailed information of primary packages for statistical analysis, machine learning, and web application construction.**

|  | **Derivation cohort** | | ***P* value** | **Training cohort** | | ***P* value** | **Internal validation cohort** | | ***P* value** |
| --- | --- | --- | --- | --- | --- | --- | --- | --- | --- |
| **Characteristics** | **Control (n=1300)** | **COPD (n=650)** |  | **Control (n=1040)** | **COPD (n=520)** |  | **Control (n=260)** | **COPD (n=130)** |  |
| **Questionnaire** |  |  |  |  |  |  |  |  |  |
| Age, Yrs, M (IQR) | 63·0 (58·0–67·0) | 63·5 (59·0–68·0) | 0·443 | 63·0 (58·8–67·0) | 64·0 (59·0–68·0) | 0·199 | 64·0 (58·0–69·0) | 63·0 (58·0–68·8) | 0·464 |
| Age group, n (%) |  |  |  |  |  |  |  |  |  |
| 35–49 | 48 (3·70) | 24 (3·70) | 1 | 37 (3·60) | 19 (3·70) | 0·993 | 11 (4·20) | 5 (3·80) | 0·951 |
| 50–59 | 322 (24·8) | 161 (24·8) |  | 259 (24·9) | 126 (24·2) |  | 63 (24·2) | 35 (26·9) |  |
| 60–69 | 716 (55·1) | 358 (55·1) |  | 582 (56·0) | 293 (56·3) |  | 134 (51·5) | 65 (50·0) |  |
| 70–80 | 214 (16·5) | 107 (16·5) |  | 162 (15·6) | 82 (15·8) |  | 52 (20·0) | 25 (19·2) |  |
| Sex, n (%) |  |  |  |  |  |  |  |  |  |
| Male | 944 (72·6) | 472 (72·6) | 1 | 756 (72·7) | 382 (73·5) | 0·793 | 188 (72·3) | 90 (69·2) | 0·607 |
| Female | 356 (27·4) | 178 (27·4) |  | 284 (27·3) | 138 (26·5) |  | 72 (27·7) | 40 (30·8) |  |
| BMI, n (%) |  |  |  |  |  |  |  |  |  |
| <18·5 | 49 (3·80) | 42 (6·50) | <0·001 | 37 (3·60) | 29 (5·60) | <0·001 | 12 (4·60) | 13 (10·0) | 0·093 |
| 18·5–23·9 | 711 (54·7) | 408 (62·8) |  | 558 (53·7) | 328 (63·1) |  | 153 (58·8) | 80 (61·5) |  |
| 24–27·9 | 448 (34·5) | 178 (27·4) |  | 367 (35·3) | 144 (27·7) |  | 81 (31·2) | 34 (26·2) |  |
| ≥ 28 | 92 (7·10) | 22 (3·40) |  | 78 (7·50) | 19 (3·70) |  | 14 (5·40) | 3 (2·30) |  |
| Smoking, n (%) |  |  |  |  |  |  |  |  |  |
| Never | 639 (49·2) | 252 (38·8) | <0·001 | 494 (47·5) | 197 (37·9) | <0·001 | 145 (55·8) | 55 (42·3) | 0·016 |
| Former or current | 661 (50·8) | 398 (61·2) |  | 546 (52·5) | 323 (62·1) |  | 115 (44·2) | 75 (57·7) |  |
| Smoking pack-years, n (%) |  |  |  |  |  |  |  |  |  |
| 0 | 639 (49·2) | 252 (38·8) | <0·001 | 494 (47·5) | 197 (37·9) | 0·003 | 145 (55·8) | 55 (42·3) | 0·086 |
| 1–14 | 134 (10·3) | 71 (10·9) |  | 106 (10·2) | 55 (10·6) |  | 28 (10·8) | 16 (12·3) |  |
| 15–30 | 191 (14·7) | 115 (17·7) |  | 157 (15·1) | 93 (17·9) |  | 34 (13·1) | 22 (16·9) |  |
| ≥ 30 | 336 (25·8) | 212 (32·6) |  | 283 (27·2) | 175 (33·7) |  | 53 (20·4) | 37 (28·5) |  |
| Quit smoking, n (%) |  |  |  |  |  |  |  |  |  |
| Non-smoker | 639 (49·2) | 252 (38·8) | <0·001 | 494 (47·5) | 197 (37·9) | <0·001 | 145 (55·8) | 55 (42·3) | 0·043 |
| Yes | 213 (16·4) | 108 (16·6) |  | 174 (16·7) | 83 (16·0) |  | 39 (15·0) | 25 (19·2) |  |
| No | 448 (34·5) | 290 (44·6) |  | 372 (35·8) | 240 (46·2) |  | 76 (29·2) | 50 (38·5) |  |
| Quit smoking over 15 years, n (%) |  |  |  |  |  |  |  |  |  |
| Non-smoker | 639 (49·2) | 252 (38·8) | <0·001 | 494 (47·5) | 197 (37·9) | 0·001 | 145 (55·8) | 55 (42·3) | 0·093 |
| No | 135 (10·4) | 70 (10·8) |  | 107 (10·3) | 53 (10·2) |  | 28 (10·8) | 17 (13·1) |  |
| Yes | 78 (6·00) | 38 (5·80) |  | 67 (6·40) | 30 (5·80) |  | 11 (4·20) | 8 (6·20) |  |
| Current smoker | 448 (34·5) | 290 (44·6) |  | 372 (35·8) | 240 (46·2) |  | 76 (29·2) | 50 (38·5) |  |
| Secondhand smoke exposure, n (%) |  |  |  |  |  |  |  |  |  |
| No | 203 (15·6) | 85 (13·1) | <0·001 | 164 (15·8) | 67 (12·9) | 0·001 | 39 (15·0) | 18 (13·8) | 0·075 |
| Yes | 436 (33·5) | 167 (25·7) |  | 330 (31·7) | 130 (25·0) |  | 106 (40·8) | 37 (28·5) |  |
| Former smoker | 213 (16·4) | 108 (16·6) |  | 174 (16·7) | 83 (16·0) |  | 39 (15·0) | 25 (19·2) |  |
| Current smoker | 448 (34·5) | 290 (44·6) |  | 372 (35·8) | 240 (46·2) |  | 76 (29·2) | 50 (38·5) |  |
| Education, n (%) |  |  |  |  |  |  |  |  |  |
| Primary school or lower | 94 (7·20) | 63 (9·70) | 0·011 | 74 (7·10) | 51 (9·80) | 0·019 | 20 (7·70) | 12 (9·20) | 0·513 |
| Middle school | 392 (30·2) | 230 (35·4) |  | 302 (29·0) | 180 (34·6) |  | 90 (34·6) | 50 (38·5) |  |
| High school | 528 (40·6) | 243 (37·4) |  | 431 (41·4) | 192 (36·9) |  | 97 (37·3) | 51 (39·2) |  |
| Associate's degree | 202 (15·5) | 75 (11·5) |  | 165 (15·9) | 63 (12·1) |  | 37 (14·2) | 12 (9·20) |  |
| Bachelor's degree or higher | 84 (6·50) | 39 (6·00) |  | 68 (6·50) | 34 (6·50) |  | 16 (6·20) | 5 (3·80) |  |
| Marital status, n (%) |  |  |  |  |  |  |  |  |  |
| Married | 1214 (93·4) | 599 (92·2) | 0·364 | 970 (93·3) | 482 (92·7) | 0·751 | 244 (93·8) | 117 (90·0) | 0·246 |
| Ever or never married | 86 (6·60) | 51 (7·80) |  | 70 (6·70) | 38 (7·30) |  | 16 (6·20) | 13 (10·0) |  |
| Occupational exposure, n (%) |  |  |  |  |  |  |  |  |  |
| No | 500 (38·5) | 241 (37·1) | 0·586 | 394 (37·9) | 192 (36·9) | 0·753 | 106 (40·8) | 49 (37·7) | 0·634 |
| Yes | 800 (61·5) | 409 (62·9) |  | 646 (62·1) | 328 (63·1) |  | 154 (59·2) | 81 (62·3) |  |
| Kitchen ventilator, n (%) |  |  |  |  |  |  |  |  |  |
| No | 210 (16·2) | 136 (20·9) | 0·011 | 168 (16·2) | 103 (19·8) | 0·085 | 42 (16·2) | 33 (25·4) | 0·041 |
| Yes | 1090 (83·8) | 514 (79·1) |  | 872 (83·8) | 417 (80·2) |  | 218 (83·8) | 97 (74·6) |  |
| Home ventilation, n (%) |  |  |  |  |  |  |  |  |  |
| Good | 990 (76·2) | 469 (72·2) | 0·038 | 796 (76·5) | 375 (72·1) | 0·07 | 194 (74·6) | 94 (72·3) | 0·448 |
| General | 293 (22·5) | 164 (25·2) |  | 229 (22·0) | 131 (25·2) |  | 64 (24·6) | 33 (25·4) |  |
| Bad | 17 (1·30) | 17 (2·60) |  | 15 (1·40) | 14 (2·70) |  | 2 (0·800) | 3 (2·30) |  |
| Indoor renovation within ten years, n (%) |  |  |  |  |  |  |  |  |  |
| No | 873 (67·2) | 435 (66·9) | 0·959 | 702 (67·5) | 354 (68·1) | 0·863 | 171 (65·8) | 81 (62·3) | 0·574 |
| Yes | 427 (32·8) | 215 (33·1) |  | 338 (32·5) | 166 (31·9) |  | 89 (34·2) | 49 (37·7) |  |
| Incense burning, n (%) |  |  |  |  |  |  |  |  |  |
| Yearly or less | 513 (39·5) | 252 (38·8) | 0·956 | 410 (39·4) | 208 (40·0) | 0·886 | 103 (39·6) | 44 (33·8) | 0·409 |
| Monthly | 537 (41·3) | 271 (41·7) |  | 431 (41·4) | 209 (40·2) |  | 106 (40·8) | 62 (47·7) |  |
| Weekly or more | 250 (19·2) | 127 (19·5) |  | 199 (19·1) | 103 (19·8) |  | 51 (19·6) | 24 (18·5) |  |
| Self-cooking, n (%) |  |  |  |  |  |  |  |  |  |
| Yearly or less | 153 (11·8) | 80 (12·3) | 0·119 | 125 (12·0) | 64 (12·3) | 0·544 | 28 (10·8) | 16 (12·3) | 0·057 |
| Monthly | 581 (44·7) | 259 (39·8) |  | 452 (43·5) | 211 (40·6) |  | 129 (49·6) | 48 (36·9) |  |
| Weekly or more | 566 (43·5) | 311 (47·8) |  | 463 (44·5) | 245 (47·1) |  | 103 (39·6) | 66 (50·8) |  |
| Cooking fuel type within ten years, n (%) |  |  |  |  |  |  |  |  |  |
| LPG or gas | 1292 (99·4) | 643 (98·9) | 0·410 | 1034 (99·4) | 515 (99·0) | 0·593 | 258 (99·2) | 128 (98·5) | 0·603 |
| Coal or firewood | 8 (0·600) | 7 (1·10) |  | 6 (0·600) | 5 (1·00) |  | 2 (0·800) | 2 (1·50) |  |
| Cooking fuel type in childhood, n (%) |  |  |  |  |  |  |  |  |  |
| LPG or gas | 55 (4·20) | 16 (2·50) | 0·066 | 41 (3·90) | 12 (2·30) | 0·126 | 14 (5·40) | 4 (3·10) | 0·443 |
| Coal or firewood | 1245 (95·8) | 634 (97·5) |  | 999 (96·1) | 508 (97·7) |  | 246 (94·6) | 126 (96·9) |  |
| Alcohol drinking, n (%) |  |  |  |  |  |  |  |  |  |
| Yearly or less | 655 (50·4) | 349 (53·7) | 0·311 | 515 (49·5) | 275 (52·9) | 0·455 | 140 (53·8) | 74 (56·9) | 0·272 |
| Monthly | 513 (39·5) | 245 (37·7) |  | 417 (40·1) | 195 (37·5) |  | 96 (36·9) | 50 (38·5) |  |
| Weekly or more | 132 (10·2) | 56 (8·60) |  | 108 (10·4) | 50 (9·60) |  | 24 (9·20) | 6 (4·60) |  |
| Tea drinking, n (%) |  |  |  |  |  |  |  |  |  |
| Yearly or less | 133 (10·2) | 64 (9·80) | 0·033 | 109 (10·5) | 50 (9·60) | 0·032 | 24 (9·20) | 14 (10·8) | 0·711 |
| Monthly | 652 (50·2) | 289 (44·5) |  | 523 (50·3) | 230 (44·2) |  | 129 (49·6) | 59 (45·4) |  |
| Weekly or more | 515 (39·6) | 297 (45·7) |  | 408 (39·2) | 240 (46·2) |  | 107 (41·2) | 57 (43·8) |  |
| Pickled food, n (%) |  |  |  |  |  |  |  |  |  |
| Yearly or less | 113 (8·70) | 64 (9·80) | 0·698 | 89 (8·60) | 54 (10·40) | 0·403 | 24 (9·20) | 10 (7·70) | 0·675 |
| Monthly | 1112 (85·5) | 550 (84·6) |  | 896 (86·2) | 443 (85·2) |  | 216 (83·1) | 107 (82·3) |  |
| Weekly or more | 75 (5·80) | 36 (5·50) |  | 55 (5·30) | 23 (4·40) |  | 20 (7·70) | 13 (10·0) |  |
| Bean food, n (%) |  |  |  |  |  |  |  |  |  |
| Yearly or less | 61 (4·70) | 36 (5·50) | 0·697 | 50 (4·80) | 31 (6·00) | 0·462 | 11 (4·20) | 5 (3·80) | 0·561 |
| Monthly | 973 (74·8) | 479 (73·7) |  | 773 (74·3) | 373 (71·7) |  | 200 (76·9) | 106 (81·5) |  |
| Weekly or more | 266 (20·5) | 135 (20·8) |  | 217 (20·9) | 116 (22·3) |  | 49 (18·8) | 19 (14·6) |  |
| Exercise, n (%) |  |  |  |  |  |  |  |  |  |
| Yearly or less | 327 (25·2) | 167 (25·7) | 0·626 | 262 (25·2) | 129 (24·8) | 0·576 | 65 (25·0) | 38 (29·2) | 0·242 |
| Monthly | 810 (62·3) | 386 (59·4) |  | 644 (61·9) | 314 (60·4) |  | 166 (63·8) | 72 (55·4) |  |
| Weekly or more | 163 (12·5) | 97 (14·9) |  | 134 (12·9) | 77 (14·8) |  | 29 (11·2) | 20 (15·4) |  |
| Pet raising, n (%) |  |  |  |  |  |  |  |  |  |
| No | 1079 (83·0) | 517 (79·5) | 0·071 | 863 (83·0) | 416 (80·0) | 0·169 | 216 (83·1) | 101 (77·7) | 0·251 |
| Yes | 221 (17·0) | 133 (20·5) |  | 177 (17·0) | 104 (20·0) |  | 44 (16·9) | 29 (22·3) |  |
| Total sleep time, h, M (IQR) | 7·00 (6·00–8·00) | 7·00 (6·00–8·00) | 0·361 | 7·00 (6·00–8·00) | 7·00 (6·00–8·00) | 0·410 | 7·00 (6·00–8·00) | 7·00 (6·00–8·00) | 0·670 |
| Mental trauma, n (%) |  |  |  |  |  |  |  |  |  |
| No | 1195 (91·9) | 589 (90·6) | 0·374 | 955 (91·8) | 474 (91·2) | 0·723 | 240 (92·3) | 115 (88·5) | 0·287 |
| Yes | 105 (8·10) | 61 (9·40) |  | 85 (8·20) | 46 (8·80) |  | 20 (7·70) | 15 (11·50) |  |
| Depressed mood, n (%) |  |  |  |  |  |  |  |  |  |
| No | 1209 (93·0) | 607 (93·4) | 0·825 | 963 (92·6) | 486 (93·5) | 0·601 | 246 (94·6) | 121 (93·1) | 0·704 |
| Yes | 91 (7·00) | 43 (6·60) |  | 77 (7·40) | 34 (6·50) |  | 14 (5·40) | 9 (6·90) |  |
| Ambient air temperature-related allergy, n (%) |  |  |  |  |  |  |  |  |  |
| No | 1144 (88·0) | 535 (82·3) | 0·001 | 912 (87·7) | 429 (82·5) | 0·007 | 232 (89·2) | 106 (81·5) | 0·051 |
| Yes | 156 (12·0) | 115 (17·7) |  | 128 (12·3) | 91 (17·5) |  | 28 (10·8) | 24 (18·5) |  |
| Hospitalized before the age of ten, n (%) |  |  |  |  |  |  |  |  |  |
| No | 1254 (96·5) | 603 (92·8) | <0·001 | 1005 (96·6) | 481 (92·5) | <0·001 | 249 (95·8) | 122 (93·8) | 0·560 |
| Yes | 46 (3·50) | 47 (7·20) |  | 35 (3·40) | 39 (7·50) |  | 11 (4·20) | 8 (6·20) |  |
| Cough, n (%) |  |  |  |  |  |  |  |  |  |
| No | 906 (69·7) | 409 (62·9) | 0·003 | 724 (69·6) | 321 (61·7) | 0·002 | 182 (70·0) | 88 (67·7) | 0·727 |
| Yes | 394 (30·3) | 241 (37·1) |  | 316 (30·4) | 199 (38·3) |  | 78 (30·0) | 42 (32·3) |  |
| Produce phlegm, n (%) |  |  |  |  |  |  |  |  |  |
| No | 870 (66·9) | 403 (62·0) | 0·036 | 689 (66·2) | 318 (61·2) | 0·054 | 181 (69·6) | 85 (65·4) | 0·465 |
| Yes | 430 (33·1) | 247 (38·0) |  | 351 (33·8) | 202 (38·8) |  | 79 (30·4) | 45 (34·6) |  |
| Tachypnea, n (%) |  |  |  |  |  |  |  |  |  |
| No | 1095 (84·2) | 506 (77·8) | 0·001 | 873 (83·9) | 406 (78·1) | 0·012 | 222 (85·4) | 100 (76·9) | 0·079 |
| Hurrying on flat ground or walking up a small slope | 184 (14·2) | 124 (19·1) |  | 149 (14·3) | 98 (18·8) |  | 35 (13·5) | 26 (20·0) |  |
| Walking at own pace on flat ground | 21 (1·60) | 20 (3·10) |  | 18 (1·70) | 16 (3·10) |  | 3 (1·20) | 4 (3·10) |  |
| Family history of respiratory disease, n (%) |  |  |  |  |  |  |  |  |  |
| No | 1199 (92·2) | 567 (87·2) | 0·001 | 956 (91·9) | 451 (86·7) | 0·002 | 243 (93·5) | 116 (89·2) | 0·209 |
| Yes | 101 (7·80) | 83 (12·8) |  | 84 (8·10) | 69 (13·3) |  | 17 (6·50) | 14 (10·8) |  |
| Underlying diseases (yes), n (%) |  |  |  |  |  |  |  |  |  |
| Chronic respiratory disease | 193 (14·8) | 212 (32·6) | <0·001 | 154 (14·8) | 165 (31·7) | <0·001 | 39 (15·0) | 47 (36·2) | <0·001 |
| Hypertension | 419 (32·2) | 193 (29·7) | 0·277 | 332 (31·9) | 150 (28·8) | 0·237 | 87 (33·5) | 43 (33·1) | 1 |
| Diabetes | 140 (10·8) | 59 (9·10) | 0·278 | 108 (10·4) | 50 (9·60) | 0·700 | 32 (12·3) | 9 (6·90) | 0·145 |
| Heart disease | 169 (13·0) | 86 (13·2) | 0·943 | 138 (13·3) | 70 (13·5) | 0·979 | 31 (11·9) | 16 (12·3) | 1 |
| Stroke | 31 (2·40) | 15 (2·30) | 1 | 24 (2·30) | 13 (2·50) | 0·953 | 7 (2·70) | 2 (1·50) | 0·724 |
| Allergic disease | 260 (20·0) | 167 (25·7) | 0·005 | 208 (20·0) | 131 (25·2) | 0·023 | 52 (20·0) | 36 (27·7) | 0·113 |
| **QCT** |  |  |  |  |  |  |  |  |  |
| LAA-950, %, M (IQR) |  |  |  |  |  |  |  |  |  |
| Lung | 3·00 (1·00–7·00) | 11·0 (7·00–16·0) | <0·001 | 3·00 (1·00–7·00) | 11·0 (6·00–15·3) | <0·001 | 3·00 (1·00–7·00) | 11·0 (7·00–17·0) | <0·001 |
| Left upper lobe | 4·00 (1·00–8·00) | 12·0 (7·00–18·0) | <0·001 | 4·00 (1·00–8·00) | 12·0 (7·00–18·0) | <0·001 | 3·50 (1·00–8·00) | 11·5 (8·00–19·0) | <0·001 |
| Left lower lobe | 2·00 (1·00–5·00) | 10·0 (5·00–15·0) | <0·001 | 2·00 (1·00–5·00) | 9·0 (5·00–15·0) | <0·001 | 2·00 (1·00–6·00) | 10·0 (5·00–15·0) | <0·001 |
| Right upper lobe | 3·00 (0–7·00) | 10·0 (6·00–17·0) | <0·001 | 3·00 (0–7·00) | 10·0 (6·00–16·0) | <0·001 | 3·00 (0–7·00) | 11·0 (6·00–18·0) | <0·001 |
| Right middle lobe | 4·00 (1·00–8·00) | 12·0 (7·00–18·0) | <0·001 | 4·00 (1·00–8·00) | 13·0 (7·00–18·0) | <0·001 | 4·00 (1·00–8·00) | 12·0 (7·00–19·0) | <0·001 |
| Right lower lobe | 2·00 (0–5·00) | 9·00 (4·00–14·0) | <0·001 | 2·00 (0–5·00) | 9·00 (4·00–14·0) | <0·001 | 2·00 (0–5·00) | 9·00 (5·00–14·0) | <0·001 |
| LAA-910, %, M (IQR) |  |  |  |  |  |  |  |  |  |
| Lung | 19·5 (11·0–27·0) | 35·0 (25·0–45·0) | <0·001 | 19·0 (11·0–27·0) | 35·0 (25·0–44·0) | <0·001 | 20·0 (11·0–28·0) | 36·0 (24·5–45·0) | <0·001 |
| Left upper lobe | 23·0 (14·0–33·0) | 38·0 (28·0–49·0) | <0·001 | 23·0 (14·0–33·0) | 38·0 (28·0–48·3) | <0·001 | 23·0 (13·8–32·3) | 38·5 (29·0–50·0) | <0·001 |
| Left lower lobe | 14·0 (7·00–23·0) | 31·0 (20·0–41·0) | <0·001 | 14·0 (7·00–22·0) | 31·0 (20·0–41·0) | <0·001 | 15·0 (7·75–23·0) | 32·0 (20·3–41·8) | <0·001 |
| Right upper lobe | 20·0 (11·0–29·0) | 35·5 (25·0–46·0) | <0·001 | 20·0 (11·0–29·0) | 35·0 (25·0–46·0) | <0·001 | 21·0 (11·0–29·0) | 36·5 (25·3–45·0) | <0·001 |
| Right middle lobe | 26·0 (16·0–37·0) | 41·0 (31·0–52·0) | <0·001 | 26·0 (16·0–37·0) | 41·0 (31·0–52·0) | <0·001 | 26·0 (17·0–37·3) | 41·5 (31·0–51·0) | <0·001 |
| Right lower lobe | 15·0 (7·00–23·0) | 31·0 (20·3–41·0) | <0·001 | 15·0 (7·00–23·0) | 31·0 (20·0–41·0) | <0·001 | 15·5 (7·00–23·0) | 31·0 (22·0–40·8) | <0·001 |
| %WA, %, M (IQR) |  |  |  |  |  |  |  |  |  |
| 0th generation | 37·0 (35·0–40·0) | 36·0 (34·0–39·0) | 0·040 | 37·0 (35·0–40·0) | 36·0 (34·0–39·0) | 0·018 | 37·0 (34·0–39·0) | 37·0 (35·0–39·0) | 0·760 |
| 1st generation | 42·0 (39·0–45·0) | 41·0 (38·0–44·0) | <0·001 | 42·0 (39·0–45·0) | 41·0 (38·0–44·0) | 0·001 | 41·5 (39·0–45·0) | 41·0 (38·0–44·0) | 0·172 |
| 2nd generation | 51·0 (47·0–54·0) | 50·0 (47·0–53·0) | 0·053 | 51·0 (48·0–54·0) | 50·0 (47·0–53·0) | 0·029 | 50·0 (47·0–54·0) | 50·0 (47·0–53·8) | 0·956 |
| 3rd generation | 62·0 (58·0–66·0) | 62·0 (58·0–66·0) | 0·351 | 62·0 (58·0–66·0) | 62·0 (58·0–66·0) | 0·586 | 61·0 (58·0–65·0) | 62·0 (59·0–66·8) | 0·316 |
| 4th generation | 68·0 (65·0–72·0) | 69·0 (65·0–73·0) | 0·018 | 68·0 (65·0–72·0) | 69·0 (65·0–73·0) | 0·037 | 68·0 (64·0–73·0) | 70·0 (65·0–72·0) | 0·283 |
| Average WT, mm, M (IQR) |  |  |  |  |  |  |  |  |  |
| 0th generation | 1·99 (1·90–2·10) | 1·97 (1·88–2·08) | 0·037 | 1·99 (1·90–2·10) | 1·98 (1·89–2·08) | 0·057 | 1·96 (1·89–2·09) | 1·95 (1·87–2·05) | 0·381 |
| 1st generation | 1·93 (1·84–2·05) | 1·92 (1·83–2·03) | 0·039 | 1·94 (1·85–2·06) | 1·93 (1·84–2·04) | 0·069 | 1·92 (1·84–2·04) | 1·90 (1·81–2·01) | 0·307 |
| 2nd generation | 1·88 (1·77–2·00) | 1·86 (1·76–1·97) | 0·062 | 1·88 (1·78–2·00) | 1·87 (1·76–1·97) | 0·077 | 1·85 (1·77–1·97) | 1·83 (1·76–1·95) | 0·494 |
| 3rd generation | 1·83 (1·74–1·95) | 1·83 (1·73–1·93) | 0·142 | 1·83 (1·74–1·96) | 1·83 (1·73–1·93) | 0·201 | 1·83 (1·73–1·94) | 1·81 (1·72–1·91) | 0·459 |
| 4th generation | 1·47 (1·40–1·55) | 1·46 (1·38–1·54) | 0·007 | 1·48 (1·40–1·56) | 1·46 (1·39–1·54) | 0·017 | 1·46 (1·40–1·55) | 1·45 (1·38–1·53) | 0·206 |
| Max WT, mm, M (IQR) |  |  |  |  |  |  |  |  |  |
| 0th generation | 2·59 (2·47–2·74) | 2·57 (2·45–2·71) | 0·094 | 2·59 (2·47–2·74) | 2·58 (2·46–2·71) | 0·147 | 2·57 (2·46–2·72) | 2·56 (2·43–2·70) | 0·386 |
| 1st generation | 2·62 (2·49–2·77) | 2·60 (2·48–2·74) | 0·124 | 2·62 (2·49–2·77) | 2·60 (2·48–2·74) | 0·120 | 2·59 (2·49–2·75) | 2·60 (2·45–2·73) | 0·734 |
| 2nd generation | 2·58 (2·44–2·72) | 2·55 (2·43–2·70) | 0·057 | 2·58 (2·45–2·72) | 2·56 (2·43–2·70) | 0·113 | 2·56 (2·44–2·71) | 2·52 (2·42–2·67) | 0·277 |
| 3rd generation | 2·48 (2·36–2·63) | 2·47 (2·34–2·61) | 0·026 | 2·49 (2·36–2·64) | 2·48 (2·34–2·61) | 0·045 | 2·48 (2·35–2·60) | 2·45 (2·33–2·60) | 0·304 |
| 4th generation | 1·47 (1·40–1·55) | 1·46 (1·38–1·54) | 0·007 | 1·96 (1·86–2·06) | 1·93 (1·84–2·03) | 0·001 | 1·95 (1·85–2·05) | 1·92 (1·81–2·02) | 0·087 |
| Min WT, mm, M (IQR) |  |  |  |  |  |  |  |  |  |
| 0th generation | 1·31 (1·24–1·39) | 1·29 (1·23–1·37) | 0·031 | 1·31 (1·24–1·40) | 1·29 (1·23–1·37) | 0·046 | 1·29 (1·23–1·38) | 1·28 (1·23–1·36) | 0·421 |
| 1st generation | 1·17 (1·09–1·25) | 1·16 (1·09–1·23) | 0·027 | 1·17 (1·10–1·26) | 1·16 (1·09–1·23) | 0·045 | 1·17 (1·08–1·24) | 1·15 (1·08–1·22) | 0·346 |
| 2nd generation | 1·11 (1·01–1·20) | 1·10 (1·01–1·18) | 0·081 | 1·11 (1·02–1·21) | 1·11 (1·02–1·18) | 0·117 | 1·10 (0·99–1·19) | 1·07 (1·00–1·16) | 0·463 |
| 3rd generation | 1·12 (1·04–1·22) | 1·13 (1·05–1·20) | 0·748 | 1·12 (1·04–1·22) | 1·13 (1·05–1·20) | 0·741 | 1·13 (1·04–1·21) | 1·13 (1·06–1·19) | 0·976 |
| 4th generation | 0·930 (0·870–1·00) | 0·930 (0·880–1·00) | 0·529 | 0·930 (0·870–1·00) | 0·940 (0·880–1·00) | 0·608 | 0·930 (0·860–0·990) | 0·920 (0·870–0·980) | 0·699 |
| Average LD, mm, M (IQR) |  |  |  |  |  |  |  |  |  |
| 0th generation | 16·5 (14·9–17·9) | 16·8 (15·1–18·1) | 0·021 | 16·5 (14·9–17·9) | 16·8 (15·3–18·1) | 0·009 | 16·5 (15·0–18·1) | 16·6 (14·8–18·0) | 0·968 |
| 1st generation | 16·0 (14·2–17·6) | 16·3 (14·5–18·2) | 0·005 | 15·9 (14·2–17·7) | 16·4 (14·6–18·3) | 0·006 | 16·1 (14·1–17·6) | 16·3 (14·2–17·9) | 0·413 |
| 2nd generation | 12·2 (10·6–13·7) | 12·4 (10·7–14·0) | 0·266 | 12·2 (10·6–13·7) | 12·4 (10·7–14·2) | 0·154 | 12·2 (10·6–13·8) | 12·2 (10·3–13·7) | 0·695 |
| 3rd generation | 8·13 (6·96–9·49) | 8·01 (6·68–9·41) | 0·067 | 8·11 (6·93–9·52) | 8·00 (6·65–9·48) | 0·111 | 8·23 (7·11–9·39) | 8·07 (7·02–9·27) | 0·343 |
| 4th generation | 5·54 (4·59–6·54) | 5·35 (4·30–6·41) | <0·001 | 5·56 (4·62–6·56) | 5·38 (4·24–6·40) | <0·001 | 5·49 (4·48–6·47) | 5·20 (4·36–6·40) | 0·192 |
| Max LD, mm, M (IQR) |  |  |  |  |  |  |  |  |  |
| 0th generation | 18·3 (16·3–20·1) | 18·9 (16·9–20·3) | <0·001 | 18·3 (16·3–20·1) | 18·9 (17·1–20·3) | <0·001 | 18·3 (16·4–20·1) | 18·6 (16·3–20·4) | 0·536 |
| 1st generation | 20·6 (18·1–23·0) | 21·3 (18·5–23·9) | 0·004 | 20·6 (18·2–23·0) | 21·4 (18·6–23·9) | 0·005 | 20·9 (18·0–23·0) | 21·1 (18·3–23·9) | 0·380 |
| 2nd generation | 16·2 (13·9–18·7) | 16·6 (14·0–19·1) | 0·293 | 16·1 (13·9–18·6) | 16·7 (14·1–19·2) | 0·177 | 16·5 (13·7–18·8) | 16·3 (13·6–18·7) | 0·685 |
| 3rd generation | 10·6 (8·91–12·8) | 10·4 (8·52–12·6) | 0·043 | 10·6 (8·87–12·8) | 10·4 (8·43–12·7) | 0·057 | 10·7 (9·04–12·6) | 10·6 (8·93–12·1) | 0·475 |
| 4th generation | 7·30 (5·92–8·84) | 6·97 (5·53–8·52) | <0·001 | 7·31 (5·95–8·90) | 7·00 (5·51–8·48) | <0·001 | 7·27 (5·80–8·63) | 6·68 (5·61–8·57) | 0·198 |
| Min LD, mm, M (IQR) |  |  |  |  |  |  |  |  |  |
| 0th generation | 14·6 (13·2–16·0) | 14·8 (13·2–15·9) | 0·509 | 14·6 (13·2–15·9) | 14·8 (13·2–16·0) | 0·338 | 14·6 (13·3–16·0) | 14·6 (13·1–15·7) | 0·652 |
| 1st generation | 11·2 (9·90–12·4) | 11·5 (10·1–12·6) | 0·023 | 11·2 (9·90–12·4) | 11·5 (10·1–12·7) | 0·022 | 11·3 (9·88–12·3) | 11·4 (9·99–12·4) | 0·666 |
| 2nd generation | 7·98 (7·16–8·91) | 8·09 (7·18–8·98) | 0·330 | 7·98 (7·15–8·91) | 8·17 (7·18–9·01) | 0·225 | 7·98 (7·19–8·93) | 7·96 (7·10–8·90) | 0·810 |
| 3rd generation | 5·60 (4·93–6·32) | 5·57 (4·78–6·35) | 0·250 | 5·57 (4·92–6·30) | 5·58 (4·76–6·40) | 0·552 | 5·69 (5·01–6·39) | 5·50 (4·82–6·15) | 0·150 |
| 4th generation | 3·73 (3·19–4·31) | 3·62 (3·03–4·17) | 0·001 | 3·74 (3·21–4·33) | 3·64 (3·01–4·18) | 0·003 | 3·71 (3·16–4·27) | 3·58 (3·10–4·12) | 0·228 |
| **CT report** |  |  |  |  |  |  |  |  |  |
| Emphysema (yes), n (%) |  |  |  |  |  |  |  |  |  |
| Lung | 317 (24·4) | 306 (47·1) | <0·001 | 254 (24·4) | 243 (46·7) | <0·001 | 63 (24·2) | 63 (48·5) | <0·001 |
| Left lung | 253 (19·5) | 272 (41·8) | <0·001 | 202 (19·4) | 221 (42·5) | <0·001 | 51 (19·6) | 51 (39·2) | <0·001 |
| Left upper lobe | 241 (18·5) | 264 (40·6) | <0·001 | 193 (18·6) | 215 (41·3) | <0·001 | 48 (18·5) | 49 (37·7) | <0·001 |
| Left lower lobe | 164 (12·6) | 233 (35·8) | <0·001 | 130 (12·5) | 187 (36·0) | <0·001 | 34 (13·1) | 46 (35·4) | <0·001 |
| Right lung | 268 (20·6) | 284 (43·7) | <0·001 | 218 (21·0) | 225 (43·3) | <0·001 | 50 (19·2) | 59 (45·4) | <0·001 |
| Right upper lobe | 236 (18·2) | 262 (40·3) | <0·001 | 192 (18·5) | 209 (40·2) | <0·001 | 44 (16·9) | 53 (40·8) | <0·001 |
| Right middle lobe | 162 (12·5) | 228 (35·1) | <0·001 | 129 (12·4) | 183 (35·2) | <0·001 | 33 (12·7) | 45 (34·6) | <0·001 |
| Right lower lobe | 178 (13·7) | 251 (38·6) | <0·001 | 142 (13·7) | 198 (38·1) | <0·001 | 36 (13·8) | 53 (40·8) | <0·001 |
| Bronchitis (yes), n (%) |  |  |  |  |  |  |  |  |  |
| Lung | 43 (3·30) | 110 (16·9) | <0·001 | 35 (3·40) | 86 (16·5) | <0·001 | 8 (3·10) | 24 (18·5) | <0·001 |
| Left lung | 38 (2·90) | 108 (16·6) | <0·001 | 32 (3·10) | 84 (16·2) | <0·001 | 6 (2·30) | 24 (18·5) | <0·001 |
| Left upper lobe | 36 (2·80) | 104 (16·0) | <0·001 | 30 (2·90) | 81 (15·6) | <0·001 | 6 (2·30) | 23 (17·7) | <0·001 |
| Left lower lobe | 36 (2·80) | 101 (15·5) | <0·001 | 30 (2·90) | 80 (15·4) | <0·001 | 6 (2·30) | 21 (16·2) | <0·001 |
| Right lung | 41 (3·20) | 107 (16·5) | <0·001 | 33 (3·20) | 84 (16·2) | <0·001 | 8 (3·10) | 23 (17·7) | <0·001 |
| Right upper lobe | 40 (3·10) | 104 (16·0) | <0·001 | 32 (3·10) | 81 (15·6) | <0·001 | 8 (3·10) | 23 (17·7) | <0·001 |
| Right middle lobe | 39 (3·00) | 103 (15·8) | <0·001 | 31 (3·00) | 81 (15·6) | <0·001 | 8 (3·10) | 22 (16·9) | <0·001 |
| Right lower lobe | 41 (3·20) | 107 (16·5) | <0·001 | 30 (2·90) | 80 (15·4) | <0·001 | 7 (2·70) | 20 (15·4) | <0·001 |
| Pulmonary nodule (yes), n (%) |  |  |  |  |  |  |  |  |  |
| Lung | 658 (50·6) | 304 (46·8) | 0·120 | 519 (49·9) | 241 (46·3) | 0·204 | 139 (53·5) | 63 (48·5) | 0·410 |
| Left lung | 387 (29·8) | 170 (26·2) | 0·107 | 305 (29·3) | 134 (25·8) | 0·158 | 82 (31·5) | 36 (27·7) | 0·508 |
| Left upper lobe | 235 (18·1) | 96 (14·8) | 0·077 | 178 (17·1) | 76 (14·6) | 0·235 | 57 (21·9) | 20 (15·4) | 0·163 |
| Left lower lobe | 231 (17·8) | 108 (16·6) | 0·568 | 191 (18·4) | 86 (16·5) | 0·412 | 40 (15·4) | 22 (16·9) | 0·807 |
| Right lung | 494 (38·0) | 238 (36·6) | 0·585 | 389 (37·4) | 190 (36·5) | 0·781 | 105 (40·4) | 48 (36·9) | 0·582 |
| Right upper lobe | 298 (22·9) | 141 (21·7) | 0·578 | 233 (22·4) | 111 (21·3) | 0·682 | 65 (25·0) | 30 (23·1) | 0·770 |
| Right middle lobe | 147 (11·3) | 76 (11·7) | 0·860 | 118 (11·3) | 62 (11·9) | 0·801 | 29 (11·2) | 14 (10·8) | 1 |
| Right lower lobe | 209 (16·1) | 127 (19·5) | 0·065 | 166 (16·0) | 100 (19·2) | 0·122 | 43 (16·5) | 27 (20·8) | 0·375 |
| Bronchiectasia (yes), n (%) |  |  |  |  |  |  |  |  |  |
| Lung | 101 (7·80) | 123 (18·9) | <0·001 | 80 (7·70) | 98 (18·8) | <0·001 | 21 (8·10) | 25 (19·2) | 0·002 |
| Left lung | 57 (4·40) | 90 (13·8) | <0·001 | 44 (4·20) | 69 (13·3) | <0·001 | 13 (5·00) | 21 (16·2) | <0·001 |
| Left upper lobe | 42 (3·20) | 61 (9·40) | <0·001 | 31 (3·00) | 51 (9·80) | <0·001 | 11 (4·20) | 10 (7·70) | 0·234 |
| Left lower lobe | 29 (2·20) | 58 (8·90) | <0·001 | 23 (2·20) | 41 (7·90) | <0·001 | 6 (2·30) | 17 (13·1) | <0·001 |
| Right lung | 82 (6·30) | 83 (12·8) | <0·001 | 64 (6·20) | 66 (12·7) | <0·001 | 18 (6·90) | 17 (13·1) | 0·069 |
| Right upper lobe | 37 (2·80) | 43 (6·60) | <0·001 | 27 (2·60) | 41 (7·90) | <0·001 | 10 (3·80) | 2 (1·50) | 0·351 |
| Right middle lobe | 44 (3·40) | 45 (6·90) | 0·001 | 35 (3·40) | 34 (6·50) | 0·006 | 9 (3·50) | 11 (8·50) | 0·062 |
| Right lower lobe | 29 (2·20) | 46 (7·10) | <0·001 | 22 (2·10) | 35 (6·70) | <0·001 | 7 (2·70) | 11 (8·50) | 0·021 |
| Fibrosis (yes), n (%) |  |  |  |  |  |  |  |  |  |
| Lung | 524 (40·3) | 244 (37·5) | 0·258 | 430 (41·3) | 194 (37·3) | 0·139 | 94 (36·2) | 50 (38·5) | 0·738 |
| Left lung | 385 (29·6) | 181 (27·8) | 0·488 | 320 (30·8) | 147 (28·3) | 0·338 | 65 (25·0) | 34 (26·2) | 0·902 |
| Left upper lobe | 295 (22·7) | 143 (22·0) | 0·774 | 243 (23·4) | 115 (22·1) | 0·624 | 52 (20·0) | 28 (21·5) | 0·825 |
| Left lower lobe | 186 (14·3) | 105 (16·2) | 0·312 | 156 (15·0) | 84 (16·2) | 0·602 | 30 (11·5) | 21 (16·2) | 0·265 |
| Right lung | 434 (33·4) | 200 (30·8) | 0·267 | 352 (33·8) | 156 (30·0) | 0·141 | 82 (31·5) | 44 (33·8) | 0·730 |
| Right upper lobe | 123 (9·50) | 69 (10·6) | 0·468 | 96 (9·20) | 51 (9·80) | 0·783 | 27 (10·4) | 18 (13·8) | 0·401 |
| Right middle lobe | 285 (21·9) | 129 (19·8) | 0·318 | 228 (21·9) | 99 (19·0) | 0·210 | 57 (21·9) | 30 (23·1) | 0·897 |
| Right lower lobe | 182 (14·0) | 107 (16·5) | 0·169 | 158 (15·2) | 89 (17·1) | 0·364 | 24 (9·20) | 18 (13·8) | 0·225 |
| Inflammation (yes), n (%) |  |  |  |  |  |  |  |  |  |
| Lung | 633 (48·7) | 383 (58·9) | <0·001 | 509 (48·9) | 308 (59·2) | <0·001 | 124 (47·7) | 75 (57·7) | 0·079 |
| Left lung | 468 (36·0) | 292 (44·9) | <0·001 | 380 (36·5) | 239 (46·0) | <0·001 | 88 (33·8) | 53 (40·8) | 0·219 |
| Left upper lobe | 348 (26·8) | 225 (34·6) | <0·001 | 281 (27·0) | 188 (36·2) | <0·001 | 67 (25·8) | 37 (28·5) | 0·656 |
| Left lower lobe | 283 (21·8) | 198 (30·5) | <0·001 | 229 (22·0) | 158 (30·4) | <0·001 | 54 (20·8) | 40 (30·8) | 0·040 |
| Right lung | 530 (40·8) | 325 (50·0) | <0·001 | 422 (40·6) | 257 (49·4) | 0·001 | 108 (41·5) | 68 (52·3) | 0·057 |
| Right upper lobe | 141 (10·8) | 113 (17·4) | <0·001 | 113 (10·9) | 87 (16·7) | 0·001 | 28 (10·8) | 26 (20·0) | 0·020 |
| Right middle lobe | 357 (27·5) | 218 (33·5) | 0·006 | 288 (27·7) | 172 (33·1) | 0·032 | 69 (26·5) | 46 (35·4) | 0·091 |
| Right lower lobe | 302 (23·2) | 212 (32·6) | <0·001 | 242 (23·3) | 174 (33·5) | <0·001 | 60 (23·1) | 38 (29·2) | 0·231 |
| Calcification (yes), n (%) |  |  |  |  |  |  |  |  |  |
| Lung | 176 (13·5) | 91 (14·0) | 0·834 | 141 (13·6) | 73 (14·0) | 0·855 | 35 (13·5) | 18 (13·8) | 1 |
| Left lung | 88 (6·80) | 44 (6·80) | 1 | 71 (6·80) | 33 (6·30) | 0·802 | 17 (6·50) | 11 (8·50) | 0·627 |
| Left upper lobe | 52 (4·00) | 31 (4·80) | 0·500 | 42 (4·00) | 22 (4·20) | 0·964 | 10 (3·80) | 9 (6·90) | 0·280 |
| Left lower lobe | 41 (3·20) | 22 (3·40) | 0·892 | 34 (3·30) | 18 (3·50) | 0·960 | 7 (2·70) | 4 (3·10) | 1 |
| Right lung | 117 (9·00) | 58 (8·90) | 1 | 95 (9·10) | 47 (9·00) | 1 | 22 (8·50) | 11 (8·50) | 1 |
| Right upper lobe | 76 (5·80) | 34 (5·20) | 0·652 | 61 (5·90) | 27 (5·20) | 0·670 | 15 (5·80) | 7 (5·40) | 1 |
| Right middle lobe | 15 (1·20) | 14 (2·20) | 0·128 | 13 (1·20) | 12 (2·30) | 0·176 | 2 (0·800) | 2 (1·50) | 0·603 |
| Right lower lobe | 32 (2·50) | 25 (3·80) | 0·117 | 27 (2·60) | 20 (3·80) | 0·228 | 5 (1·90) | 5 (3·80) | 0·428 |
| Tuberculosis (yes), n (%) |  |  |  |  |  |  |  |  |  |
| Lung | 51 (3·90) | 74 (11·4) | <0·001 | 43 (4·10) | 61 (11·7) | <0·001 | 8 (3·10) | 13 (10·0) | 0·009 |
| Left lung | 30 (2·30) | 47 (7·20) | <0·001 | 24 (2·30) | 39 (7·50) | <0·001 | 6 (2·30) | 8 (6·20) | 0·102 |
| Left upper lobe | 29 (2·20) | 47 (7·20) | <0·001 | 23 (2·20) | 39 (7·50) | <0·001 | 6 (2·30) | 8 (6·20) | 0·102 |
| Left lower lobe | 8 (0·600) | 14 (2·20) | 0·005 | 5 (0·500) | 12 (2·30) | 0·003 | 3 (1·20) | 2 (1·50) | 1 |
| Right lung | 43 (3·30) | 66 (10·2) | <0·001 | 36 (3·50) | 54 (10·4) | <0·001 | 7 (2·70) | 12 (9·20) | 0·010 |
| Right upper lobe | 39 (3·00) | 66 (10·2) | <0·001 | 32 (3·10) | 54 (10·4) | <0·001 | 7 (2·70) | 12 (9·20) | 0·010 |
| Right middle lobe | 4 (0·300) | 10 (1·50) | 0·006 | 3 (0·300) | 10 (1·90) | 0·002 | 1 (0·400) | 0 (0) | 1 |
| Right lower lobe | 12 (0·900) | 17 (2·60) | 0·007 | 9 (0·900) | 16 (3·10) | 0·002 | 3 (1·20) | 1 (0·800) | 1 |
| Pleural thickness (yes), n (%) | 63 (4·80) | 63 (9·70) | <0·001 | 53 (5·10) | 51 (9·80) | 0·001 | 10 (3·80) | 12 (9·20) | 0·052 |
| Abbreviations: COPD, chronic obstructive pulmonary disease; Yrs, years; M (IQR), median, interquartile range; BMI, body mass index; LPG: liquefied petroleum gas; CT, computed tomography; QCT, quantitative computed tomography; LAA-950, low-attenuating area below -950 Hounsfield Units; LAA-910, low-attenuating area below -910 Hounsfield Units; %WA, airway wall area percentage; WT, wall thickness; LD, lumen diameter. | | | | | | | | | |

**Table S5: Questionnaire, QCT measurements and CT report characteristics in the overall derivation cohort and split cohorts.**

| **Characteristics** | **Control** | **COPD** | ***P* value** |
| --- | --- | --- | --- |
| **External validation cohort 1** |  |  |  |
| Total number | 385 | 801 |  |
| Age, Yrs, M (IQR) | 57·0 (50·0–66·0) | 67·0 (61·0–72·0) | <0·001 |
| Age group, n (%) |  |  |  |
| 35–49 | 91 (23·6) | 21 (2·60) | <0·001 |
| 50–59 | 127 (33·0) | 133 (16·6) |  |
| 60–69 | 115 (29·9) | 346 (43·2) |  |
| 70–80 | 52 (13·5) | 301 (37·6) |  |
| Sex, n (%) |  |  |  |
| Male | 186 (48·3) | 765 (95·5) | <0·001 |
| Female | 199 (51·7) | 36 (4·50) |  |
| BMI, n (%) |  |  |  |
| <18·5 | 44 (11·4) | 184 (23·0) | <0·001 |
| 18·5–23·9 | 181 (47·0) | 433 (54·1) |  |
| 24–27·9 | 118 (30·6) | 152 (19·0) |  |
| ≥ 28 | 42 (10·9) | 32 (4·00) |  |
| Smoking, n (%) |  |  |  |
| Never | 287 (74·5) | 151 (18·9) | <0·001 |
| Former or current | 98 (25·5) | 650 (81·1) |  |
| Smoking pack-years, n (%) |  |  |  |
| 0 | 287 (74·5) | 151 (18·9) | <0·001 |
| 1–14 | 12 (3·10) | 3 (0·400) |  |
| 15–30 | 36 (9·40) | 26 (3·20) |  |
| ≥ 30 | 50 (13·0) | 621 (77·5) |  |
| GOLD stage, n (%) |  |  |  |
| 1 | NA | 25 (3·10) | NA |
| 2 | NA | 235 (29·3) | NA |
| 3 | NA | 297 (37·1) | NA |
| 4 | NA | 244 (30·5) | NA |
| Underlying diseases (yes), n (%) |  |  |  |
| Hypertension | 87 (22·6) | 292 (36·5) | <0·001 |
| Diabetes | 55 (14·3) | 131 (16·4) | 0·405 |
| Heart disease | 69 (17·9) | 235 (29·3) | <0·001 |
| Stroke | 5 (1·30) | 28 (3·50) | 0·049 |
| LAA-950, %, M (IQR) |  |  |  |
| Lung | 5·00 (2·00–10·0) | 27·0 (16·00–38·0) | <0·001 |
| Left upper lobe | 6·00 (2·00–12·0) | 26·0 (16·00–40·0) | <0·001 |
| Left lower lobe | 4·00 (1·00–9·00) | 25·0 (13·0–37·0) | <0·001 |
| Right upper lobe | 5·00 (2·00–10·0) | 27·0 (14·0–42·0) | <0·001 |
| Right middle lobe | 6·00 (3·00–12·0) | 22·0 (14·0–34·0) | <0·001 |
| Right lower lobe | 3·00 (1·00–8·00) | 23·0 (12·0–35·0) | <0·001 |
| **External validation cohort 2** |  |  |  |
| Total number | 108 | 117 |  |
| Age, Yrs, M (IQR) | 61·0 (53·0–68·0) | 70·0 (64·0–74·0) | <0·001 |
| Age group, n (%) |  |  |  |
| 35–49 | 16 (14·8) | 1 (0·900) | <0·001 |
| 50–59 | 32 (29·6) | 20 (17·1) |  |
| 60–69 | 37 (34·3) | 37 (31·6) |  |
| 70–80 | 23 (21·3) | 59 (50·4) |  |
| Sex, n (%) |  |  |  |
| Male | 57 (52·8) | 100 (85·5) | <0·001 |
| Female | 51 (47·2) | 17 (14·5) |  |
| BMI, n (%) |  |  |  |
| <18·5 | 7 (6·50) | 19 (16·2) | 0·032 |
| 18·5–23·9 | 42 (38·9) | 52 (44·4) |  |
| 24–27·9 | 44 (40·7) | 38 (32·5) |  |
| ≥ 28 | 15 (13·9) | 8 (6·80) |  |
| Smoking, n (%) |  |  |  |
| Never | 75 (69·4) | 44 (37·6) | <0·001 |
| Former or current | 33 (30·6) | 73 (62·4) |  |
| Smoking pack-years, n (%) |  |  |  |
| 0 | 75 (69·4) | 44 (37·6) | <0·001 |
| 1–14 | 3 (2·80) | 4 (3·40) |  |
| 15–30 | 10 (9·30) | 44 (37·6) |  |
| ≥ 30 | 20 (18·5) | 25 (21·4) |  |
| GOLD stage, n (%) |  |  |  |
| 1 | NA | 4 (3·40) | NA |
| 2 | NA | 30 (25·6) | NA |
| 3 | NA | 52 (44·4) | NA |
| 4 | NA | 31 (26·5) | NA |
| Underlying diseases (yes), n (%) |  |  |  |
| Hypertension | 47 (43·5) | 74 (63·2) | 0·005 |
| Diabetes | 15 (13·9) | 24 (20·5) | 0·256 |
| Heart disease | 24 (22·2) | 35 (29·9) | 0·246 |
| Stroke | 11 (10·2) | 14 (12·0) | 0·832 |
| LAA-950, %, M (IQR) |  |  |  |
| Lung | 1·50 (0–7·25) | 26·0 (15·0–35·0) | <0·001 |
| Left upper lobe | 2·00 (0–9·00) | 26·0 (17·0–37·0) | <0·001 |
| Left lower lobe | 1·00 (0–4·25) | 23·0 (12·0–33·0) | <0·001 |
| Right upper lobe | 1·00 (0–7·00) | 26·00 (14·0–38·0) | <0·001 |
| Right middle lobe | 2·00 (1·00–9·25) | 26·00 (16·0–37·0) | <0·001 |
| Right lower lobe | 1·00 (0–5·00) | 22·0 (12·0–31·0) | <0·001 |
| **External validation cohort 3** |  |  |  |
| Total number | 92 | 200 |  |
| Age, Yrs, M (IQR) | 60·5 (52·0–68·0) | 66·0 (59·0–72·0) | <0·001 |
| Age group, n (%) |  |  |  |
| 35–49 | 18 (19·6) | 9 (4·50) | <0·001 |
| 50–59 | 24 (26·1) | 43 (21·5) |  |
| 60–69 | 32 (34·8) | 81 (40·5) |  |
| 70–80 | 18 (19·6) | 67 (33·5) |  |
| Sex, n (%) |  |  |  |
| Male | 52 (56·5) | 162 (81·0) | <0·001 |
| Female | 40 (43·5) | 38 (19·0) |  |
| BMI, n (%) |  |  |  |
| <18·5 | 3 (3·30) | 16 (8·00) | 0·108 |
| 18·5–23·9 | 38 (41·3) | 98 (49·0) |  |
| 24–27·9 | 34 (37·0) | 64 (32·0) |  |
| ≥ 28 | 17 (18·5) | 22 (11·0) |  |
| Smoking, n (%) |  |  |  |
| Never | 69 (75·0) | 74 (37·0) | <0·001 |
| Former or current | 23 (25·0) | 126 (63·0) |  |
| Smoking pack-years, n (%) |  |  |  |
| 0 | 69 (75·0) | 74 (37·0) | <0·001 |
| 1–14 | 6 (6·50) | 15 (7·50) |  |
| 15–30 | 8 (8·70) | 35 (17·5) |  |
| ≥ 30 | 9 (9·80) | 76 (38·0) |  |
| GOLD stage, n (%) |  |  |  |
| 1 | NA | 31 (15·5) | NA |
| 2 | NA | 94 (47·0) | NA |
| 3 | NA | 53 (26·5) | NA |
| 4 | NA | 22 (11·0) | NA |
| Underlying diseases (yes), n (%) |  |  |  |
| Hypertension | 19 (20·7) | 53 (26·5) | 0·352 |
| Diabetes | 12 (13·0) | 17 (8·50) | 0·320 |
| Heart disease | 6 (6·50) | 35 (17·5) | 0·020 |
| Stroke | 1 (1·10) | 14 (7·00) | 0·066 |
| LAA-950, %, M (IQR) |  |  |  |
| Lung | 1·00 (0–5·25) | 18·0 (9·00–27·0) | <0·001 |
| Left upper lobe | 1·00 (0–6·00) | 19·0 (11·0–28·0) | <0·001 |
| Left lower lobe | 1·00 (0–4·00) | 15·5 (7·75–25·3) | <0·001 |
| Right upper lobe | 1·00 (0–4·25) | 19·0 (8·75–30·0) | <0·001 |
| Right middle lobe | 2·00 (0–5·00) | 18·0 (9·00–28·0) | <0·001 |
| Right lower lobe | 1·00 (0–3·00) | 14·0 (6·75–22·3) | <0·001 |
| **External validation cohort 4** |  |  |  |
| Total number | 341 | 112 |  |
| Age, Yrs, M (IQR) | 61·0 (57·0–64·0) | 64·0 (60·0–69·0) | <0·001 |
| Age group, n (%) |  |  |  |
| 50–59 | 134 (39·3) | 27 (24·1) | <0·001 |
| 60–69 | 180 (52·8) | 60 (53·6) |  |
| 70–80 | 27 (7·90) | 25 (22·3) |  |
| Sex, n (%) |  |  |  |
| Male | 182 (53·4) | 64 (57·1) | 0·558 |
| Female | 159 (46·6) | 48 (42·9) |  |
| BMI, n (%) |  |  |  |
| <18·5 | 4 (1·20) | 3 (2·70) | 0·369 |
| 18·5–23·9 | 90 (26·4) | 37 (33·0) |  |
| 24–27·9 | 113 (33·1) | 33 (29·5) |  |
| ≥ 28 | 132 (38·7) | 38 (33·9) |  |
| Unknown | 2 (0·600) | 1 (0·900) |  |
| Smoking (Former or current), n (%) | 341 (100) | 112 (100) | NA |
| Smoking pack-years (≥ 30), n (%) | 341 (100) | 112 (100) | NA |
| Education, n (%) |  |  |  |
| Middle school or lower | 6 (1·80) | 0 (0) | 0·051 |
| High school | 142 (41·6) | 41 (36·6) |  |
| Associate's degree | 62 (18·2) | 34 (30·4) |  |
| Bachelor's degree or higher | 121 (35·5) | 36 (32·1) |  |
| Other | 10 (2·90) | 1 (0·900) |  |
| Underlying diseases (yes), n (%) |  |  |  |
| Chronic respiratory disease | 60 (17·6) | 78 (69·6) | <0·001 |
| Hypertension | 114 (33·4) | 41 (36·6) | 0·617 |
| Diabetes | 27 (7·90) | 13 (11·6) | 0·316 |
| Heart disease | 47 (13·8) | 23 (20·5) | 0·118 |
| Stroke | 7 (2·10) | 4 (3·60) | 0·447 |
| LAA-950, %, M (IQR) |  |  |  |
| Lung | 5·00 (3·00–9·00) | 23·0 (13·0–32·0) | <0·001 |
| Left upper lobe | 6·00 (3·00–12·0) | 27·0 (14·8–37·0) | <0·001 |
| Left lower lobe | 4·00 (2·00–8·00) | 20·0 (10·0–30·0) | <0·001 |
| Right upper lobe | 5·00 (2·00–10·0) | 25·0 (14·0–34·3) | <0·001 |
| Right middle lobe | 7·00 (3·00–13·0) | 22·0 (13·8–36·0) | <0·001 |

| Right lower lobe | 3·00 (1·00–7·00) | 18·0 (9·00–28·3) | <0·001 |
| --- | --- | --- | --- |
| Abbreviations: COPD, chronic obstructive pulmonary disease; Yrs, years; M (IQR), median, interquartile range; BMI, body mass index; GOLD, Global Initiative for Chronic Obstructive Lung Disease; LAA-950, low-attenuating area below -950 Hounsfield Units; NA, not applicable. | | | |

| **Table S6: Demographic characteristics for the external validation cohorts.** |
| --- |

| **Schemes** | **Number of features** | **Features** |
| --- | --- | --- |
| Questionnaire | 36 | Age, Age group, Sex, BMI, Smoking, Smoking pack-years, Quit smoking, Quit smoking over 15 years, Secondhand smoke exposure, Education, Marital status, Occupational exposure, Kitchen ventilator, Home ventilation, Indoor renovation within ten years, Incense burning, Self-cooking, Alcohol drinking, Tea drinking, Pickled food, Bean food, Exercise, Pet raising, Total sleep time, Mental trauma, Depressed mood, Ambient air temperature-related allergy, Cough, Produce phlegm, Tachypnea, Family history of respiratory disease, Chronic respiratory disease, Hypertension, Diabetes, Heart disease, Stroke, Allergic disease |
| QCT | 47 | LAA-950 lung, LAA-950 left upper lobe, LAA-950 left lower lobe, LAA-950 right upper lobe, LAA-950 right middle lobe, LAA-950 right lower lobe, LAA-910 lung, LAA-910 left upper lobe, LAA-910 left lower lobe, LAA-910 right upper lobe, LAA-910 right middle lobe, LAA-910 right lower lobe, %WA of 0th generation, %WA of 1st generation, %WA of 2nd generation, %WA of 3rd generation, %WA of 4th generation, Average WT of 0th generation, Average WT of 1st generation, Average WT of 2nd generation, Average WT of 3rd generation, Average WT of 4th generation, Max WT of 0th generation, Max WT of 1st generation, Max WT of 2nd generation, Max WT of 3rd generation, Max WT of 4th generation, Min WT of 0th generation, Min WT of 1st generation, Min WT of 2nd generation, Min WT of 3rd generation, Min WT of 4th generation, Average LD of 0th generation, Average LD of 1st generation, Average LD of 2nd generation, Average LD of 3rd generation, Average LD of 4th generation, Max LD of 0th generation, Max LD of 1st generation, Max LD of 2nd generation, Max LD of 3rd generation, Max LD of 4th generation, Min LD of 0th generation, Min LD of 1st generation, Min LD of 2nd generation, Min LD of 3rd generation, Min LD of 4th generation |
| CT report | 52 | Emphysema of lung, Emphysema of left lung, Emphysema of left upper lobe, Emphysema of left lower lobe, Emphysema of right lung, Emphysema of right upper lobe, Emphysema of right middle lobe, Emphysema of right lower lobe, Bronchitis of lung, Bronchitis of left lung, Bronchitis of left upper lobe, Bronchitis of left lower lobe, Bronchitis of right lung, Bronchitis of right upper lobe, Bronchitis of right middle lobe, Bronchitis of right lower lobe, Pulmonary nodule of lung, Pulmonary nodule of left lung, Pulmonary nodule of left upper lobe, Pulmonary nodule of left lower lobe, Pulmonary nodule of right lung, Pulmonary nodule of right upper lobe, Pulmonary nodule of right middle lobe, Pulmonary nodule of right lower lobe, Bronchiectasia of lung, Bronchiectasia of left lung, Bronchiectasia of left upper lobe, Bronchiectasia of right lung, Fibrosis of lung, Fibrosis of left lung, Fibrosis of left upper lobe, Fibrosis of left lower lobe, Fibrosis of right lung, Fibrosis of right upper lobe, Fibrosis of right middle lobe, Fibrosis of right lower lobe, Inflammation of lung, Inflammation of left lung, Inflammation of left upper lobe, Inflammation of left lower lobe, Inflammation of right lung, Inflammation of right upper lobe, Inflammation of right middle lobe, Inflammation of right lower lobe, Calcification of lung, Calcification of left lung, Calcification of right lung, Calcification of right upper lobe, Tuberculosis of lung, Tuberculosis of right lung, Tuberculosis of right upper lobe, Pleural thickness |
| Questionnaire + QCT | 20 | BMI, Smoking, Smoking pack-years, Quit smoking, Quit smoking over 15 years, Secondhand smoke exposure, Education, Cough, Family history of respiratory disease, Chronic respiratory disease, LAA-950 lung, LAA-950 left upper lobe, LAA-950 left lower lobe, LAA-950 right upper lobe, LAA-950 right middle lobe, LAA-950 right lower lobe, LAA-910 left lower lobe, LAA-910 right lower lobe, Max LD of 1st generation, Average LD of 4th generation |
| Questionnaire + CT report | 20 | BMI, Smoking, Smoking pack-years, Quit smoking, Quit smoking over 15 years, Secondhand smoke exposure, Education, Cough, Family history of respiratory disease, Chronic respiratory disease, Emphysema of left lung, Emphysema of right upper, Emphysema of right lower, Bronchitis of left upper, Pulmonary nodule of right lower, Bronchiectasia of lung, Bronchiectasia of left lung, Inflammation of right lower, Calcification, Tuberculosis of right upper |
| QCT + CT report | 20 | LAA-950 lung, LAA-950 left upper lobe, LAA-950 left lower lobe, LAA-950 right upper lobe, LAA-950 right middle lobe, LAA-950 right lower lobe, LAA-910 left lower lobe, LAA-910 right lower lobe, Max LD of 1st generation, Average LD of 4th generation, Emphysema of left lung, Emphysema of right upper, Emphysema of right lower, Bronchitis of left upper, Pulmonary nodule of right lower, Bronchiectasia of lung, Bronchiectasia of left lung, Inflammation of right lower, Calcification, Tuberculosis of right upper |
| Questionnaire + QCT + CT report | 30 | BMI, Smoking, Smoking pack-years, Quit smoking, Quit smoking over 15 years, Secondhand smoke exposure, Education, Cough, Family history of respiratory disease, Chronic respiratory disease, LAA-950 lung, LAA-950 left upper lobe, LAA-950 left lower lobe, LAA-950 right upper lobe, LAA-950 right middle lobe, LAA-950 right lower lobe, LAA-910 left lower lobe, LAA-910 right lower lobe, Max LD of 1st generation, Average LD of 4th generation, Emphysema of left lung, Emphysema of right upper lobe, Emphysema of right lower lobe, Bronchitis of left upper lobe, Pulmonary nodule of right lower lobe, Bronchiectasia of lung, Bronchiectasia of left lung, Inflammation of right lower lobe, Calcification, Tuberculosis of right upper lobe |
| Abbreviations: BMI, body mass index; QCT, quantitative computed tomography; CT, computed tomography; LAA-950, low-attenuating area below -950 Hounsfield Units; LAA-910, low-attenuating area below -910 Hounsfield Units; %WA, airway wall area percentage; WT, wall thickness; LD, lumen diameter. | | |

**Table S7: Selected features of seven schemes.**

| **Subgroups** | **AUC(95%CI)** | **Sensitivity(%)** | **Specificity(%)** | **Accuracy(%)** | **PPV(%)** | **NPV(%)** | **F1 score** |
| --- | --- | --- | --- | --- | --- | --- | --- |
| **External validation cohort 1** |  |  |  |  |  |  |  |
| Overall performance | 0·915 (0·898–0·931) | 86·6 | 80·5 | 84·7 | 90·2 | 74·3 | 0·884 |
| Sex |  |  |  |  |  |  |  |
| Male (n = 951) | 0·920 (0·900–0·940) | 85·1 | 86·0 | 85·3 | 96·2 | 58·4 | 0·903 |
| Female (n = 235) | 0·787 (0·697–0·877) | 80·6 | 67·8 | 69·8 | 31·2 | 95·1 | 0·450 |
| Age |  |  |  |  |  |  |  |
| 35–49 years (n = 112) | 0·849 (0·755–0·944) | 76·2 | 83·5 | 82·1 | 51·6 | 93·8 | 0·615 |
| 50–59 years (n = 260) | 0·899 (0·863–0·935) | 77·4 | 87·4 | 82·3 | 86·6 | 78·7 | 0·817 |
| 60–69 years (n = 461) | 0·934 (0·912–0·956) | 80·1 | 94·8 | 83·7 | 97·9 | 61·2 | 0·881 |
| 70–80 years (n = 353) | 0·933 (0·902–0·963) | 87·7 | 86·5 | 87·5 | 97·4 | 54·9 | 0·923 |
| BMI |  |  |  |  |  |  |  |
| <18·5 (n = 228) | 0·960 (0·937–0·984) | 86·4 | 93·2 | 87·7 | 98·1 | 62·1 | 0·919 |
| 18·5–23·9 (n = 614) | 0·927 (0·906–0·949) | 85·4 | 86·2 | 85·7 | 93·7 | 71·2 | 0·894 |
| 24–27·9 (n = 270) | 0·891 (0·854–0·929) | 77·6 | 86·4 | 81·5 | 88·1 | 75·0 | 0·825 |
| ≥ 28 (n = 74) | 0·714 (0·593–0·834) | 75·0 | 64·3 | 68·9 | 61·5 | 77·1 | 0·676 |
| Former or current smoker |  |  |  |  |  |  |  |
| Yes (n = 748) | 0·924 (0·897–0·952) | 86·9 | 86·7 | 86·9 | 97·8 | 50·0 | 0·920 |
| No (n = 438) | 0·884 (0·850–0·917) | 75·5 | 86·1 | 82·4 | 74·0 | 87·0 | 0·748 |
| CT Apparatus |  |  |  |  |  |  |  |
| SIEMENS (n = 731) | 0·955 (0·938–0·972) | 89·9 | 91·4 | 90·2 | 98·4 | 60·4 | 0·940 |
| GE (n = 267) | 0·868 (0·827–0·910) | 61·2 | 96·4 | 79·4 | 94·0 | 72·7 | 0·742 |
| NMS (n = 160) | 0·947 (0·896–0·997) | 84·2 | 95·7 | 94·4 | 72·7 | 97·8 | 0·780 |
| TOSHIBA (n = 28) | 0·926 (NA) | 92·6 | 100 | 92·9 | 100 | 33·3 | 0·962 |
| Slice thickness |  |  |  |  |  |  |  |
| ≤ 1 mm (n = 1047) | 0·919 (0·903–0·935) | 83·8 | 86·4 | 84·7 | 91·7 | 74·9 | 0·875 |
| 1–2 mm (n = 111) | 0·920 (0·851–0·989) | 73·8 | 100 | 75·5 | 100 | 20·9 | 0·850 |
| Small airway dysfunction |  |  |  |  |  |  |  |
| Yes (n = 939) | 0·915 (0·893–0·938) | 83·9 | 86·0 | 84·2 | 97·1 | 49·0 | 0·900 |
| No (n = 247) | 0·865 (0·697–1·00) | 60·0 | 98·3 | 97·6 | 42·9 | 99·2 | 0·500 |
| Blood EOS count |  |  |  |  |  |  |  |
| ≥ 300 / μl (n = 278) | 0·928 (0·894–0·962) | 79·4 | 94·5 | 82·4 | 98·3 | 53·1 | 0·878 |
| < 300 / μl (n = 908) | 0·920 (0·903–0·937) | 84·8 | 85·2 | 84·9 | 90·9 | 76·2 | 0·877 |
| Emphysema (CT report) |  |  |  |  |  |  |  |
| Yes (n = 803) | 0·927 (0·900–0·953) | 86·7 | 84·8 | 86·6 | 98·1 | 41·1 | 0·921 |
| No (n = 383) | 0·765 (0·704–0·827) | 79·2 | 61·8 | 65·3 | 34·3 | 92·2 | 0·478 |
| Bronchitis (CT report) |  |  |  |  |  |  |  |
| Yes (n = 686) | 0·922 (0·887–0·957) | 85·2 | 86·2 | 85·3 | 98·5 | 35·0 | 0·914 |
| No (n = 500) | 0·868 (0·834–0·902) | 75·7 | 84·7 | 81·6 | 72·4 | 86·8 | 0·740 |
| Pulmonary nodule (CT report) |  |  |  |  |  |  |  |
| Yes (n = 439) | 0·899 (0·870–0·927) | 78·5 | 86·6 | 81·1 | 92·5 | 65·8 | 0·849 |
| No (n = 747) | 0·931 (0·914–0·949) | 84·3 | 88·9 | 85·8 | 94·0 | 73·2 | 0·889 |
| Bronchiectasis (CT report) |  |  |  |  |  |  |  |
| Yes (n = 271) | 0·886 (0·846–0·927) | 79·5 | 84·0 | 80·8 | 92·1 | 63·6 | 0·853 |
| No (n = 915) | 0·928 (0·912–0·944) | 84·1 | 87·8 | 85·4 | 93·3 | 73·4 | 0·885 |
| Fibrosis (CT report) |  |  |  |  |  |  |  |
| Yes (n = 451) | 0·938 (0·917–0·960) | 87·1 | 88·1 | 87·4 | 95·4 | 70·7 | 0·911 |
| No (n = 735) | 0·907 (0·886–0·928) | 82·5 | 85·4 | 83·5 | 90·8 | 73·5 | 0·865 |
| **External validation cohort 2** |  |  |  |  |  |  |  |
| Overall performance | 0·903 (0·864–0·943) | 84·6 | 85·2 | 84·9 | 86·1 | 83·6 | 0·853 |
| Sex |  |  |  |  |  |  |  |
| Male (n = 157) | 0·929 (0·882–0·976) | 89·0 | 87·7 | 88·5 | 92·7 | 82·0 | 0·908 |
| Female (n = 68) | 0·901 (0·816–0·985) | 88·2 | 84·3 | 85·3 | 65·2 | 95·6 | 0·750 |
| Age |  |  |  |  |  |  |  |
| 35–49 years (n = 17) | 0·875 (NA) | 100 | 87·5 | 88·2 | 33·3 | 100 | 0·500 |
| 50–59 years (n = 52) | 0·942 (0·873–1·00) | 95·0 | 87·5 | 90·4 | 82·6 | 96·6 | 0·884 |
| 60–69 years (n = 74) | 0·926 (0·859–0·992) | 86·5 | 91·9 | 89·2 | 91·4 | 87·2 | 0·889 |
| 70–80 years (n = 82) | 0·938 (0·887–0·990) | 91·5 | 82·6 | 89·0 | 93·1 | 79·2 | 0·923 |
| BMI |  |  |  |  |  |  |  |
| <18·5 (n = 26) | 0·955 (0·881–1·00) | 84·2 | 100 | 88·5 | 100 | 70·0 | 0·914 |
| 18·5–23·9 (n = 94) | 0·949 (0·906–0·993) | 88·5 | 95·2 | 91·5 | 95·8 | 87·0 | 0·920 |
| 24–27·9 (n = 82) | 0·921 (0·857–0·984) | 89·5 | 88·6 | 89·0 | 87·2 | 90·7 | 0·883 |
| ≥ 28 (n = 23) | 0·800 (0·616–0·984) | 100 | 60·0 | 73·9 | 57·1 | 100 | 0·727 |
| Former or current smoker |  |  |  |  |  |  |  |
| Yes (n = 106) | 0·911 (0·836–0·985) | 89·0 | 87·9 | 88·7 | 94·2 | 78·4 | 0·915 |
| No (n = 119) | 0·922 (0·873–0·971) | 86·4 | 88·0 | 87·4 | 80·9 | 91·7 | 0·835 |
| CT Apparatus |  |  |  |  |  |  |  |
| SIEMENS (n = 66) | 0·947 (0·893–1·00) | 93·1 | 89·2 | 90·9 | 87·1 | 94·3 | 0·900 |
| Philips (n = 77) | 0·936 (0·877–0·995) | 91·4 | 90·5 | 90·9 | 88·9 | 92·7 | 0·901 |
| UIH (n = 82) | 0·889 (0·813–0·965) | 86·8 | 79·3 | 84·1 | 88·5 | 76·7 | 0·876 |
| Slice thickness |  |  |  |  |  |  |  |
| ≤ 1 mm (n = 178) | 0·940 (0·907–0·973) | 88·0 | 89·7 | 88·8 | 91·7 | 85·4 | 0·898 |
| 1–2 mm (n = 47) | 0·896 (0·801–0·991) | 94·1 | 83·3 | 87·2 | 76·2 | 96·2 | 0·842 |
| Small airway dysfunction |  |  |  |  |  |  |  |
| Yes (n = 162) | 0·898 (0·838–0·957) | 87·9 | 84·8 | 87·0 | 93·6 | 73·6 | 0·907 |
| No (n = 63) | 1·00 (NA) | 100 | 100 | 100 | 100 | 100 | 1·00 |
| Blood EOS count |  |  |  |  |  |  |  |
| ≥ 300 / μl (n = 30) | 0·831 (0·658–1·00) | 80·0 | 93·3 | 86·7 | 92·3 | 82·4 | 0·857 |
| < 300 / μl (n = 195) | 0·935 (0·900–0·970) | 89·2 | 88·2 | 88·7 | 89·2 | 88·2 | 0·892 |
| Emphysema (CT report) |  |  |  |  |  |  |  |
| Yes (n = 120) | 0·891 (0·812–0·970) | 88·3 | 80·8 | 86·7 | 94·3 | 65·6 | 0·912 |
| No (n = 105) | 0·900 (0·824–0·976) | 87·0 | 89·0 | 88·6 | 69·0 | 96·1 | 0·769 |
| Bronchitis (CT report) |  |  |  |  |  |  |  |
| Yes (n = 55) | 0·922 (0·851–0·993) | 85·3 | 90·5 | 87·3 | 93·5 | 79·2 | 0·892 |
| No (n = 170) | 0·931 (0·893–0·969) | 89·2 | 87·4 | 88·2 | 87·1 | 89·4 | 0·881 |
| Pulmonary nodule (CT report) |  |  |  |  |  |  |  |
| Yes (n = 177) | 0·946 (0·914–0·979) | 92·8 | 88·3 | 90·4 | 87·5 | 93·3 | 0·901 |
| No (n = 48) | 0·821 (0·687–0·956) | 79·4 | 78·6 | 79·2 | 90·0 | 61·1 | 0·844 |
| Bronchiectasis (CT report) |  |  |  |  |  |  |  |
| Yes (n = 31) | 0·994 (0·978–1·00) | 95·8 | 100 | 96·8 | 100 | 87·5 | 0·979 |
| No (n = 194) | 0·927 (0·889–0·965) | 90·3 | 86·1 | 88·1 | 85·7 | 90·6 | 0·880 |
| Fibrosis (CT report) |  |  |  |  |  |  |  |
| Yes (n = 34) | 0·962 (0·892–1·00) | 100 | 88·2 | 94·1 | 89·5 | 100 | 0·944 |
| No (n = 191) | 0·920 (0·881–0·960) | 85·0 | 87·9 | 86·4 | 88·5 | 84·2 | 0·867 |
| **External validation cohort 3** |  |  |  |  |  |  |  |
| Overall performance | 0·914 (0·882–0·947) | 77·5 | 89·1 | 81·2 | 93·9 | 64·6 | 0·849 |
| Sex |  |  |  |  |  |  |  |
| Male (n = 214) | 0·907 (0·863–0·951) | 77·2 | 90·4 | 80·4 | 96·2 | 56·0 | 0·856 |
| Female (n = 78) | 0·928 (0·872–0·984) | 100 | 80·0 | 89·7 | 82·6 | 100 | 0·905 |
| Age |  |  |  |  |  |  |  |
| 35–49 years (n = 27) | 0·914 (0·808–1·00) | 100 | 66·7 | 77·8 | 60·0 | 100 | 0·750 |
| 50–59 years (n = 67) | 0·958 (0·918–0·999) | 90·7 | 87·5 | 89·6 | 92·9 | 84·0 | 0·918 |
| 60–69 years (n = 113) | 0·892 (0·828–0·956) | 76·5 | 90·6 | 80·5 | 95·4 | 60·4 | 0·849 |
| 70–80 years (n = 85) | 0·877 (0·781–0·972) | 82·1 | 83·3 | 82·4 | 94·8 | 55·6 | 0·880 |
| BMI |  |  |  |  |  |  |  |
| <18·5 (n = 19) | 0·938 (0·798–1·00) | 81·3 | 100 | 84·2 | 100 | 50·0 | 0·897 |
| 18·5–23·9 (n = 136) | 0·936 (0·891–0·981) | 87·8 | 86·8 | 87·5 | 94·5 | 73·3 | 0·910 |
| 24–27·9 (n = 98) | 0·893 (0·827–0·958) | 100 | 61·8 | 86·7 | 83·1 | 100 | 0·908 |
| ≥ 28 (n = 39) | 0·890 (0·790–0·991) | 100 | 64·7 | 84·6 | 78·6 | 100 | 0·880 |
| Former or current smoker |  |  |  |  |  |  |  |
| Yes (n = 149) | 0·883 (0·809–0·957) | 75·4 | 87·0 | 77·2 | 96·9 | 39·2 | 0·848 |
| No (n = 143) | 0·930 (0·892–0·969) | 82·4 | 87·0 | 84·6 | 87·1 | 82·2 | 0·847 |
| CT Apparatus |  |  |  |  |  |  |  |
| SIEMENS (n = 159) | 0·914 (0·872–0·956) | 76·3 | 91·9 | 82·4 | 93·7 | 71·3 | 0·841 |
| GE (n = 62) | 0·925 (0·820–1·00) | 100 | 81·8 | 96·8 | 96·2 | 100 | 0·981 |
| Other (n = 71) | 0·964 (0·919–1·00) | 94·2 | 89·5 | 93·0 | 96·1 | 85·0 | 0·951 |
| Slice thickness |  |  |  |  |  |  |  |
| ≤ 1 mm (n = 72) | 0·933 (0·873–0·994) | 95·2 | 80·0 | 88·9 | 87·0 | 92·3 | 0·909 |
| 1–2 mm (n = 220) | 0·912 (0·873–0·951) | 84·8 | 80·6 | 83·6 | 91·8 | 67·6 | 0·882 |
| Small airway dysfunction |  |  |  |  |  |  |  |
| Yes (n = 231) | 0·889 (0·829–0·950) | 70·4 | 90·6 | 73·2 | 97·9 | 33·0 | 0·819 |
| No (n = 61) | 1·00 (NA) | 100 | 100 | 100 | 100 | 100 | 1·00 |
| Blood EOS count |  |  |  |  |  |  |  |
| ≥ 300 / μl (n = 43) | 0·969 (0·921–1·00) | 100 | 81·8 | 95·3 | 94·1 | 100 | 0·970 |
| < 300 / μl (n = 242) | 0·912 (0·875–0·949) | 75·8 | 90·1 | 80·6 | 93·8 | 65·2 | 0·838 |
| Emphysema (CT report) |  |  |  |  |  |  |  |
| Yes (n = 191) | 0·903 (0·848–0·958) | 82·5 | 83·8 | 82·7 | 95·5 | 53·4 | 0·885 |
| No (n = 101) | 0·917 (0·867–0·967) | 80·4 | 85·5 | 83·2 | 82·2 | 83·9 | 0·813 |
| Bronchitis (CT report) |  |  |  |  |  |  |  |
| Yes (n = 53) | 0·983 (0·947–1·00) | 91·7 | 100 | 92·5 | 100 | 55·6 | 0·957 |
| No (n = 239) | 0·908 (0·870–0·945) | 73·7 | 90·8 | 79·9 | 93·3 | 66·4 | 0·824 |
| Pulmonary nodule (CT report) |  |  |  |  |  |  |  |
| Yes (n = 169) | 0·932 (0·893–0·972) | 88·9 | 80·8 | 86·4 | 91·2 | 76·4 | 0·900 |
| No (n = 123) | 0·902 (0·847–0·956) | 72·3 | 92·5 | 78·9 | 95·2 | 61·7 | 0·822 |
| Bronchiectasis (CT report) |  |  |  |  |  |  |  |
| Yes (n = 39) | 0·911 (0·824–0·999) | 84·6 | 84·6 | 84·6 | 91·7 | 73·3 | 0·880 |
| No (n = 253) | 0·925 (0·891–0·958) | 81·0 | 88·6 | 83·4 | 94·0 | 68·0 | 0·870 |
| Fibrosis (CT report) |  |  |  |  |  |  |  |
| Yes (n = 74) | 0·886 (0·814–0·957) | 70·5 | 90·0 | 78·4 | 91·2 | 67·5 | 0·795 |
| No (n = 218) | 0·929 (0·892–0·966) | 92·9 | 77·4 | 88·5 | 91·2 | 81·4 | 0·921 |
| **External validation cohort 4** |  |  |  |  |  |  |  |
| Overall performance | 0·881 (0·846–0·915) | 81·3 | 81·2 | 81·2 | 58·7 | 93·0 | 0·682 |
| Sex |  |  |  |  |  |  |  |
| Male (n = 246) | 0·890 (0·845–0·936) | 78·1 | 87·9 | 85·4 | 69·4 | 92·0 | 0·735 |
| Female (n = 207) | 0·889 (0·841–0·936) | 93·8 | 69·2 | 74·9 | 47·9 | 97·3 | 0·634 |
| Age |  |  |  |  |  |  |  |
| 50–59 years (n = 161) | 0·894 (0·838–0·949) | 96·3 | 67·9 | 72·7 | 37·7 | 98·9 | 0·542 |
| 60–69 years (n = 240) | 0·852 (0·798–0·906) | 80·0 | 78·9 | 79·2 | 55·8 | 92·2 | 0·658 |
| 70–80 years (n = 52) | 0·920 (0·839–1·00) | 88·0 | 88·9 | 88·5 | 88·0 | 88·9 | 0·880 |
| BMI |  |  |  |  |  |  |  |
| <18·5 (n = 7) | 0·833 (0·456–1·00) | 66·7 | 100 | 85·7 | 100 | 80·0 | 0·800 |
| 18·5–23·9 (n = 127) | 0·873 (0·809–0·938) | 78·4 | 83·3 | 81·9 | 65·9 | 90·4 | 0·716 |
| 24–27·9 (n = 146) | 0·904 (0·843–0·965) | 87·9 | 79·6 | 81·5 | 55·8 | 95·7 | 0·682 |
| ≥ 28 (n = 170) | 0·888 (0·835–0·941) | 73·7 | 91·7 | 87·6 | 71·8 | 92·4 | 0·727 |
| Education |  |  |  |  |  |  |  |
| Middle school or lower (n = 6) | NA | NA | NA | NA | NA | NA | NA |
| High school (n = 183) | 0·887 (0·835–0·939) | 70·7 | 91·5 | 86·9 | 70·7 | 91·5 | 0·707 |
| Associate's degree (n = 96) | 0·865 (0·794–0·936) | 73·5 | 87·1 | 82·3 | 75·8 | 85·7 | 0·746 |
| Bachelor's degree or higher (n = 157) | 0·902 (0·838–0·965) | 77·8 | 91·7 | 88·5 | 73·7 | 93·3 | 0·757 |
| CT Apparatus |  |  |  |  |  |  |  |
| SIEMENS (n = 332) | 0·866 (0·821–0·912) | 80·3 | 79·7 | 79·8 | 54·0 | 93·2 | 0·646 |
| GE (n = 86) | 0·940 (0·894–0·987) | 100 | 80·9 | 84·9 | 58·1 | 100 | 0·735 |
| Other (n = 35) | 0·935 (0·858–1·00) | 100 | 76·5 | 88·6 | 81·8 | 100 | 0·900 |
| Slice thickness |  |  |  |  |  |  |  |
| ≤ 1 mm (n = 19) | 1·00 (1·00–1·00) | 100 | 100 | 100 | 100 | 100 | 1·00 |
| > 1 mm (n = 434) | 0·873 (0·836–0·910) | 76·8 | 82·2 | 80·9 | 56·3 | 92·2 | 0·650 |
| Chronic respiratory disease |  |  |  |  |  |  |  |
| Yes (n = 138) | 0·897 (0·848–0·947) | 73·1 | 93·3 | 81·9 | 93·4 | 72·7 | 0·820 |
| No (n = 315) | 0·870 (0·814–0·927) | 85·3 | 77·2 | 78·1 | 31·2 | 97·7 | 0·457 |
| Emphysema |  |  |  |  |  |  |  |
| Yes (n = 69) | 0·912 (0·845–0·979) | 81·1 | 93·8 | 84·1 | 97·7 | 60·0 | 0·887 |
| No (n = 384) | 0·848 (0·800–0·897) | 71·2 | 82·5 | 80·7 | 42·4 | 94·0 | 0·532 |
| Bronchitis |  |  |  |  |  |  |  |
| Yes (n = 75) | 0·927 (0·867–0·986) | 97·7 | 77·4 | 89·3 | 86·0 | 96·0 | 0·915 |
| No (n = 378) | 0·878 (0·834–0·922) | 79·4 | 81·6 | 81·2 | 48·6 | 94·8 | 0·603 |
| Pulmonary nodule |  |  |  |  |  |  |  |
| Yes (n = 252) | 0·886 (0·840–0·932) | 79·0 | 80·5 | 80·2 | 57·0 | 92·2 | 0·662 |
| No (n = 201) | 0·883 (0·834–0·933) | 86·0 | 77·5 | 79·6 | 55·8 | 94·4 | 0·677 |
| Bronchiectasis |  |  |  |  |  |  |  |
| Yes (n = 17) | 0·955 (0·857–1·00) | 100 | 90·9 | 94·1 | 85·7 | 100 | 0·923 |
| No (n = 436) | 0·879 (0·843–0·914) | 77·4 | 82·1 | 81·0 | 58·2 | 91·9 | 0·664 |
| Fibrosis |  |  |  |  |  |  |  |
| Yes (n = 2) | NA | NA | NA | NA | NA | NA | NA |
| No (n = 451) | 0·882 (0·848–0·916) | 78·2 | 82·1 | 81·2 | 58·5 | 92·1 | 0·669 |
| Abbreviations: AUC, area under the receiver operating characteristic curve; CI, confidence interval; NPV, negative predictive value; PPV, positive predictive value; CT, computed tomography; EOS, eosinophil; NA, not applicable. The AUC, sensitivity, specificity, accuracy, NPV, PPV and F1 score were calculated using Youden’s index. | | | | | | | |
| **Table S8: COPD detection performance for AutoCOPD in various subgroups of the external validation cohorts.** | | | | | | | |

| **Characteristics** | **Overall (n=1950)** | **Control (n=1300)** | **COPD (n=650)** |
| --- | --- | --- | --- |
| Age, % | 0 | 0 | 0 |
| Sex, % | 0 | 0 | 0 |
| BMI, % | 0 | 0 | 0 |
| Smoking, % | 0 | 0 | 0 |
| Smoking pack-years, % | 0·210 | 0·150 | 0·310 |
| Quit smoking, % | 0 | 0 | 0 |
| Quit smoking over 15 years, % | 0·100 | 0 | 0·310 |
| Secondhand smoke exposure, % | 0·360 | 0·230 | 0·620 |
| Education, % | 0·620 | 0·080 | 1·69 |
| Marital status, % | 0·620 | 0·080 | 1·69 |
| Occupational exposure, % | 0·620 | 0·080 | 1·69 |
| Kitchen ventilator, % | 0·620 | 0·080 | 1·69 |
| Home ventilation, % | 0·620 | 0·080 | 1·69 |
| Indoor renovation within ten years, % | 0·620 | 0·080 | 1·69 |
| Incense burning, % | 0·620 | 0·080 | 1·69 |
| Self-cooking, % | 0·620 | 0·080 | 1·69 |
| Cooking fuel type within ten years, % | 0 | 0 | 0 |
| Cooking fuel type in childhood, % | 0·620 | 0·080 | 1·69 |
| Alcohol drinking, % | 0·620 | 0·080 | 1·69 |
| Tea drinking, % | 0·620 | 0·080 | 1·69 |
| Pickled food, % | 0·620 | 0·080 | 1·69 |
| Bean food, % | 0·620 | 0·080 | 1·69 |
| Exercise, % | 0·620 | 0·080 | 1·69 |
| Pet raising, % | 0·620 | 0·080 | 1·69 |
| Total sleep time, % | 0 | 0 | 0 |
| Mental trauma, % | 0 | 0 | 0 |
| Depressed mood, % | 0 | 0 | 0 |
| Ambient air temperature-related allergy, % | 0 | 0 | 0 |
| Hospitalized before the age of ten, % | 0 | 0 | 0 |
| Cough, % | 0 | 0 | 0 |
| Produce phlegm, % | 0 | 0 | 0 |
| Tachypnea, % | 0 | 0 | 0 |
| Family history of respiratory disease, % | 0 | 0 | 0 |
| Underlying diseases, % |  |  |  |
| Chronic respiratory disease | 0 | 0 | 0 |
| Hypertension | 0 | 0 | 0 |
| Diabetes | 0 | 0 | 0 |
| Heart disease | 0 | 0 | 0 |
| Stroke | 0 | 0 | 0 |
| Allergic disease | 0 | 0 | 0 |
| LAA-950, % |  |  |  |
| Lung | 0 | 0 | 0 |
| Left upper lobe | 0 | 0 | 0 |
| Left lower lobe | 0 | 0 | 0 |
| Right upper lobe | 0 | 0 | 0 |
| Right middle lobe | 0 | 0 | 0 |
| Right lower lobe | 0 | 0 | 0 |
| LAA-910, % |  |  |  |
| Lung | 0 | 0 | 0 |
| Left upper lobe | 0 | 0 | 0 |
| Left lower lobe | 0 | 0 | 0 |
| Right upper lobe | 0 | 0 | 0 |
| Right middle lobe | 0 | 0 | 0 |
| Right lower lobe | 0 | 0 | 0 |
| %WA, % |  |  |  |
| 0th generation | 0 | 0 | 0 |
| 1st generation | 0·050 | 0 | 0·150 |
| 2nd generation | 0·050 | 0 | 0·150 |
| 3rd generation | 0·150 | 0 | 0·460 |
| 4th generation | 0·150 | 0 | 0·460 |
| Average WT, % |  |  |  |
| 0th generation | 0 | 0 | 0 |
| 1st generation | 0·050 | 0 | 0·150 |
| 2nd generation | 0·050 | 0 | 0·150 |
| 3rd generation | 0·150 | 0 | 0·460 |
| 4th generation | 0·150 | 0 | 0·460 |
| Max WT, % |  |  |  |
| 0th generation | 0 | 0 | 0 |
| 1st generation | 0·050 | 0 | 0·150 |
| 2nd generation | 0·050 | 0 | 0·150 |
| 3rd generation | 0·150 | 0 | 0·460 |
| 4th generation | 0·150 | 0 | 0·460 |
| Min WT, % |  |  |  |
| 0th generation | 0 | 0 | 0 |
| 1st generation | 0·050 | 0 | 0·150 |
| 2nd generation | 0·050 | 0 | 0·150 |
| 3rd generation | 0·150 | 0 | 0·460 |
| 4th generation | 0·150 | 0 | 0·460 |
| Average LD, % |  |  |  |
| 0th generation | 0 | 0 | 0 |
| 1st generation | 0·050 | 0 | 0·150 |
| 2nd generation | 0·050 | 0 | 0·150 |
| 3rd generation | 0·150 | 0 | 0·460 |
| 4th generation | 0·150 | 0 | 0·460 |
| Max LD, % |  |  |  |
| 0th generation | 0 | 0 | 0 |
| 1st generation | 0·050 | 0 | 0·150 |
| 2nd generation | 0·050 | 0 | 0·150 |
| 3rd generation | 0·150 | 0 | 0·460 |
| 4th generation | 0·150 | 0 | 0·460 |
| Min LD, % |  |  |  |
| 0th generation | 0 | 0 | 0 |
| 1st generation | 0·050 | 0 | 0·150 |
| 2nd generation | 0·050 | 0 | 0·150 |
| 3rd generation | 0·150 | 0 | 0·460 |
| 4th generation | 0·150 | 0 | 0·460 |
| Emphysema, % |  |  |  |
| Lung | 0 | 0 | 0 |
| Left lung | 0 | 0 | 0 |
| Left upper lobe | 0 | 0 | 0 |
| Left lower lobe | 0 | 0 | 0 |
| Right lung | 0 | 0 | 0 |
| Right upper lobe | 0 | 0 | 0 |
| Right middle lobe | 0 | 0 | 0 |
| Right lower lobe | 0 | 0 | 0 |
| Bronchitis, % |  |  |  |
| Lung | 0 | 0 | 0 |
| Left lung lobe | 0 | 0 | 0 |
| Left upper lobe | 0 | 0 | 0 |
| Left lower lobe | 0 | 0 | 0 |
| Right lung | 0 | 0 | 0 |
| Right upper lobe | 0 | 0 | 0 |
| Right middle lobe | 0 | 0 | 0 |
| Right lower lobe | 0 | 0 | 0 |
| Pulmonary nodule, % |  |  |  |
| Lung | 0 | 0 | 0 |
| Left lung | 0 | 0 | 0 |
| Left upper lobe | 0 | 0 | 0 |
| Left lower lobe | 0 | 0 | 0 |
| Right lung | 0 | 0 | 0 |
| Right upper lobe | 0 | 0 | 0 |
| Right middle lobe | 0 | 0 | 0 |
| Right lower lobe | 0 | 0 | 0 |
| Bronchiectasia, % |  |  |  |
| Lung | 0 | 0 | 0 |
| Left lung | 0 | 0 | 0 |
| Left upper lobe | 0 | 0 | 0 |
| Left lower lobe | 0 | 0 | 0 |
| Right lung | 0 | 0 | 0 |
| Right upper lobe | 0 | 0 | 0 |
| Right middle lobe | 0 | 0 | 0 |
| Right lower lobe | 0 | 0 | 0 |
| Fibrosis, % |  |  |  |
| Lung | 0 | 0 | 0 |
| Left lung | 0 | 0 | 0 |
| Left upper lobe | 0 | 0 | 0 |
| Left lower lobe | 0 | 0 | 0 |
| Right lung | 0 | 0 | 0 |
| Right upper lobe | 0 | 0 | 0 |
| Right middle lobe | 0 | 0 | 0 |
| Right lower lobe | 0 | 0 | 0 |
| Inflammation, % |  |  |  |
| Lung | 0 | 0 | 0 |
| Left lung | 0 | 0 | 0 |
| Left upper lobe | 0 | 0 | 0 |
| Left lower lobe | 0 | 0 | 0 |
| Right lung | 0 | 0 | 0 |
| Right upper lobe | 0 | 0 | 0 |
| Right middle lobe | 0 | 0 | 0 |
| Right lower lobe | 0 | 0 | 0 |
| Calcification, % |  |  |  |
| Lung | 0 | 0 | 0 |
| Left lung | 0 | 0 | 0 |
| Left upper lobe | 0 | 0 | 0 |
| Left lower lobe | 0 | 0 | 0 |
| Right lung | 0 | 0 | 0 |
| Right upper lobe | 0 | 0 | 0 |
| Right middle lobe | 0 | 0 | 0 |
| Right lower lobe | 0 | 0 | 0 |
| Tuberculosis, % |  |  |  |
| Lung | 0 | 0 | 0 |
| Left lung | 0 | 0 | 0 |
| Left upper lobe | 0 | 0 | 0 |
| Left lower lobe | 0 | 0 | 0 |
| Right lung | 0 | 0 | 0 |
| Right upper lobe | 0 | 0 | 0 |
| Right middle lobe | 0 | 0 | 0 |
| Right lower lobe | 0 | 0 | 0 |
| Pleural thickness, % | 0 | 0 | 0 |
| Blood EOS count, % | 45·6% | 43·8% | 49·2% |
| Abbreviations: COPD, chronic obstructive pulmonary disease; BMI, body mass index; LAA-950, low-attenuating area below -950 Hounsfield Units; LAA-910, low-attenuating area below -910 Hounsfield Units; %WA, airway wall area percentage; WT, wall thickness; LD, lumen diameter; EOS, eosinophil. | | | |

**Table S9: Missingness of derivation cohort.**

| **Characteristics** | **Overall** | **Control** | **COPD** |
| --- | --- | --- | --- |
| **External validation cohort 1** |  |  |  |
| Total number | 1186 | 385 | 801 |
| Age, % | 0 | 0 | 0 |
| Sex, % | 0 | 0 | 0 |
| BMI, % | 0 | 0 | 0 |
| Smoking, % | 0 | 0 | 0 |
| Smoking pack-years, % | 0 | 0 | 0 |
| Underlying diseases, % |  |  |  |
| Hypertension | 0 | 0 | 0 |
| Diabetes | 0 | 0 | 0 |
| Heart disease | 0 | 0 | 0 |
| Stroke | 0 | 0 | 0 |
| LAA-950, % |  |  |  |
| Lung | 0 | 0 | 0 |
| Left upper lobe | 0 | 0 | 0 |
| Left lower lobe | 0 | 0 | 0 |
| Right upper lobe | 0 | 0 | 0 |
| Right middle lobe | 0 | 0 | 0 |
| Right lower lobe | 0 | 0 | 0 |
| LAA-910, % |  |  |  |
| Left lower lobe | 0 | 0 | 0 |
| Right lower lobe | 0 | 0 | 0 |
| Max LD of 1st generation, % | 0·050 | 0 | 0·150 |
| Average LD of 4th generation, % | 0·150 | 0 | 0·460 |
| Emphysema, % | 0 | 0 | 0 |
| Bronchitis, % | 0 | 0 | 0 |
| Pulmonary nodule, % | 0 | 0 | 0 |
| Bronchiectasia, % | 0 | 0 | 0 |
| Fibrosis, % | 0 | 0 | 0 |
| Blood EOS count, % | 0 | 0 | 0 |
| **External validation cohort 2** |  |  |  |
| Total number | 225 | 108 | 117 |
| Age, % | 0 | 0 | 0 |
| Sex, % | 0 | 0 | 0 |
| BMI, % | 0 | 0 | 0 |
| Smoking, % | 0 | 0 | 0 |
| Smoking pack-years, % | 0 | 0 | 0 |
| Underlying diseases, % |  |  |  |
| Hypertension | 0 | 0 | 0 |
| Diabetes | 0 | 0 | 0 |
| Heart disease | 0 | 0 | 0 |
| Stroke | 0 | 0 | 0 |
| LAA-950, % |  |  |  |
| Lung | 0 | 0 | 0 |
| Left upper lobe | 0 | 0 | 0 |
| Left lower lobe | 0 | 0 | 0 |
| Right upper lobe | 0 | 0 | 0 |
| Right middle lobe | 0 | 0 | 0 |
| Right lower lobe | 0 | 0 | 0 |
| LAA-910, % |  |  |  |
| Left lower lobe | 0 | 0 | 0 |
| Right lower lobe | 0 | 0 | 0 |
| Max LD of 1st generation | 12·0 | 6·48 | 12·1 |
| Average LD of 4th generation | 12·4 | 4·63 | 19·7 |
| Emphysema, % | 0 | 0 | 0 |
| Bronchitis, % | 0 | 0 | 0 |
| Pulmonary nodule, % | 0 | 0 | 0 |
| Bronchiectasia, % | 0 | 0 | 0 |
| Fibrosis, % | 0 | 0 | 0 |
| Blood EOS count, % | 0 | 0 | 0 |
| **External validation cohort 3** |  |  |  |
| Total number | 292 | 92 | 200 |
| Age, % | 0 | 0 | 0 |
| Sex, % | 0 | 0 | 0 |
| BMI, % | 0 | 0 | 0 |
| Smoking, % | 0 | 0 | 0 |
| Smoking pack-years, % | 0 | 0 | 0 |
| Underlying diseases, % |  |  |  |
| Hypertension | 0 | 0 | 0 |
| Diabetes | 0 | 0 | 0 |
| Heart disease | 0 | 0 | 0 |
| Stroke | 0 | 0 | 0 |
| LAA-950, % |  |  |  |
| Lung | 0 | 0 | 0 |
| Left upper lobe | 0 | 0 | 0 |
| Left lower lobe | 0 | 0 | 0 |
| Right upper lobe | 0 | 0 | 0 |
| Right middle lobe | 0 | 0 | 0 |
| Right lower lobe | 0 | 0 | 0 |
| LAA-910, % |  |  |  |
| Left lower lobe | 0 | 0 | 0 |
| Right lower lobe | 0 | 0 | 0 |
| Max LD of 1st generation, % | 5·48 | 6·52 | 5·00 |
| Average LD of 4th generation, % | 6·16 | 4·35 | 7·00 |
| Emphysema, % | 0 | 0 | 0 |
| Bronchitis, % | 0 | 0 | 0 |
| Pulmonary nodule, % | 0 | 0 | 0 |
| Bronchiectasia, % | 0 | 0 | 0 |
| Fibrosis, % | 0 | 0 | 0 |
| Blood EOS count, % | 2·40 | 0 | 3·50 |
| **External validation cohort 4** |  |  |  |
| Total number | 453 | 341 | 112 |
| Age, % | 0 | 0 | 0 |
| Sex, % | 0 | 0 | 0 |
| BMI, % | 0·660 | 0·590 | 0·890 |
| Smoking, % | 0 | 0 | 0 |
| Smoking pack-years, % | 0 | 0 | 0 |
| Education, % | 0 | 0 | 0 |
| Underlying diseases, % |  |  |  |
| Chronic respiratory disease | 0 | 0 | 0 |
| Hypertension | 0 | 0 | 0 |
| Diabetes | 0 | 0 | 0 |
| Heart disease | 0 | 0 | 0 |
| Stroke | 0 | 0 | 0 |
| LAA-950, % |  |  |  |
| Lung | 0 | 0 | 0 |
| Left upper lobe | 0 | 0 | 0 |
| Left lower lobe | 0 | 0 | 0 |
| Right upper lobe | 0 | 0 | 0 |
| Right middle lobe | 0 | 0 | 0 |
| Right lower lobe | 0 | 0 | 0 |
| LAA-910, % |  |  |  |
| Left lower lobe | 0 | 0 | 0 |
| Right lower lobe | 0 | 0 | 0 |
| Max LD of 1st generation, % | 3·53 | 3·23 | 4·46 |
| Average LD of 4th generation, % | 3·97 | 3·52 | 5·36 |
| Emphysema, % | 0 | 0 | 0 |
| Bronchitis, % | 0 | 0 | 0 |
| Pulmonary nodule, % | 0 | 0 | 0 |
| Bronchiectasia, % | 0 | 0 | 0 |
| Fibrosis, % | 0 | 0 | 0 |
| Abbreviations: COPD, chronic obstructive pulmonary disease; BMI, body mass index; LAA-950, low-attenuating area below -950 Hounsfield Units; LAA-910, low-attenuating area below -910 Hounsfield Units; %WA, airway wall area percentage; WT, wall thickness; LD, lumen diameter; EOS, eosinophil. | | | |

**Table S10: Missingness of external validation cohorts.**


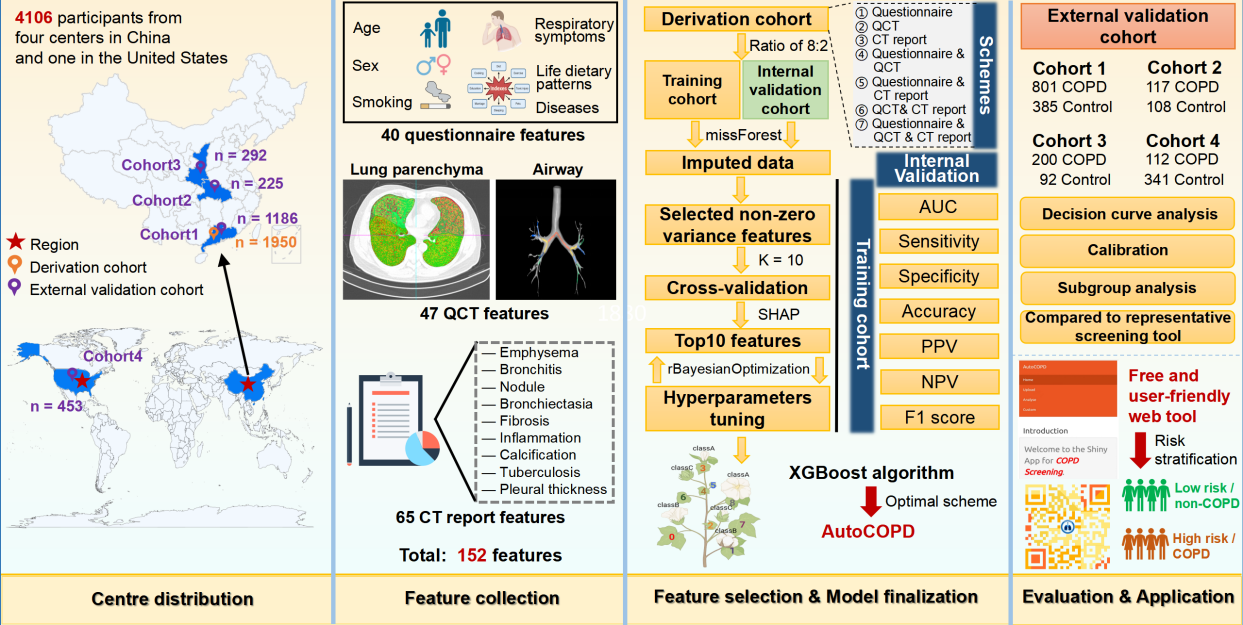


**Figure S1: Graphical abstract.** The diagram displays the development and evaluation of AutoCOPD. Briefly, a total of 40 questionnaire features, 47 QCT features and 65 CT report features were selected, and seven schemes were trained using the XGBoost algorithm. The participants of derivation cohort were randomly split into training (80%) and internal validation cohorts (20%). Feature selection, model development, and hyperparameter tuning were performed in the training cohort and the validation in the internal and external validation cohorts. Ten-fold cross-validation was used to compare the performance of different models, and models’ hyperparameters were tuned by Bayesian optimization. Top ten features selection and contribution were using the SHAP values to interpret. For evaluating overall discriminatory ability of models, AUC, sensitivity, specificity, accuracy, PPV, NPV, and F1 score were estimated. The performance of the optimal model, referred to as AutoCOPD, was further evaluated through decision curve analysis, calibration curve, subgroup analysis and a comparison with a representative screening tool (COPD-SQ). Finally, a web browser-accessible version of AutoCOPD was made available for clinical practice (<https://lwj-lab.shinyapps.io/autocopd/>).

Abbreviations: QCT, quantitative computed tomography; CT, computed tomography; XGBoost, eXtreme gradient boosting; SHAP, SHapley Additive exPlanation; AUC, area under the receiver operating characteristic curve; PPV, positive predictive value; NPV, negative predictive value; COPD-SQ, chronic obstructive pulmonary disease screening questionnaire.


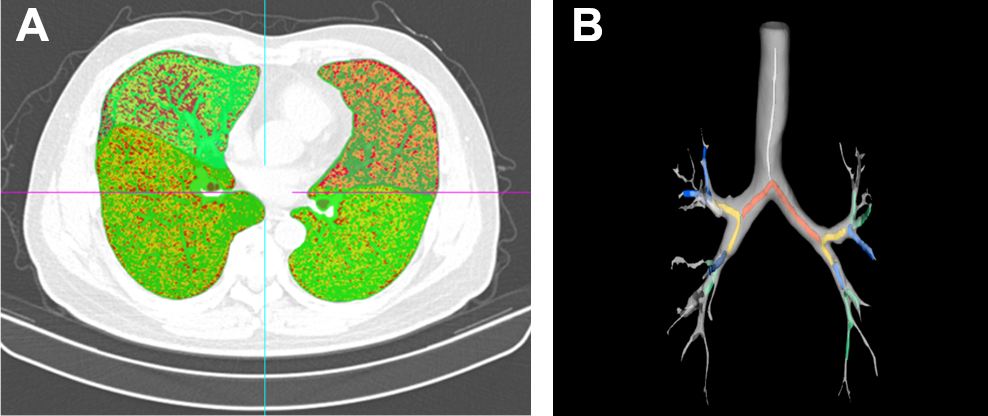


**Figure S2: QCT measurements of emphysema and airway tree. (A)** Lung voxels ≤ -950 HU in CT value are annotated by red color; lung voxels > -950 HU and -910 HU in CT value are annotated by yellow color; lung voxels > -910 HU in CT value are annotated by green color. **(B)** The trachea is assigned to 0th generation labeled white color; main bronchi are assigned to 1st generation labeled red color; lobar bronchi are assigned to 2nd generation labeled yellow color; segmental bronchi are assigned to 3rd generation labeled light blue and sub-segmental bronchi are assigned to 4th generation labeled green.

Abbreviations: QCT, quantitative computed tomography; CT, computed tomographic; HU, Hounsfield Unit.
